# PPP2R2A insufficiency enhances PD-L1 immune checkpoint blockade efficacy in lung cancer through cGAS-STING activation

**DOI:** 10.1172/JCI193354

**Published:** 2025-12-18

**Authors:** Zhaojun Qiu, No-Joon Song, Anqi Li, Deepika Singh, Chandra B. Prasad, Chunhong Yan, David P. Carbone, Qi-en Wang, Xiaoli Zhang, Zihai Li, Junran Zhang

**Affiliations:** 1 The Department of Radiation Oncology, The Ohio State University Comprehensive Cancer Center and College of Medicine, Columbus, Ohio, USA.; 2 College of Medicine, and; 3 The James Comprehensive Cancer Center, Pelotonia Institute for Immuno-Oncology, The Ohio State University, Columbus, Ohio, USA.; 4 Georgia Cancer Center, Augusta University Medical College, Augusta, Georgia, USA.; 5 James Comprehensive Cancer Center, The Ohio State University, Columbus, Ohio, USA.; 6 USF Health, University of South Florida, Tampa, Florida, USA.; 7 The James Comprehensive Cancer Center, Center for Metabolism, Columbus, Ohio, USA.

**Keywords:** Cell biology, Immunology, Cancer immunotherapy

## Abstract

PP2A B55α, a regulatory subunit of protein phosphatase 2 (PP2A), is underexpressed in greater than 40% of non–small cell lung cancer (NSCLC) cases due to loss of heterozygosity of *PPP2R2A*, the gene encoding this protein. Given that low PPP2R2A expression correlates with poor prognosis, treating PPP2R2A-deficient NSCLC represents an unmet medical need. Here, we show that *PPP2R2A* knockdown or its heterozygosity (*PPP2R2A^+/–^*) increases cytosolic DNA, leading to cGAS-STING-type I IFN pathway activation. PPP2R2A deficiency results in elevated expression of immune checkpoint protein PD-L1 via GSK-3β- and STING-dependent mechanisms. *PPP2R2A^+/–^* cancer cells have enhanced sensitivity to PD-L1 blockade in a mouse model of lung cancer due to modulation of the tumor immune microenvironment, resulting in increased NK cells and reduced infiltration and function of Tregs. Consequently, PD-L1 antibody treatment increases CD8^+^ T infiltration and activity, especially in tumors with *PPP2R2A* heterozygosity. Furthermore, systemic or Treg-specific IFNAR1 blockade reduces the efficacy of PD-L1 blockade in *PPP2R2A^+/–^* tumors. Patients with NSCLC with a low PPP2R2A/PD-L1 ratio respond better to immune checkpoint blockade (ICB). These findings underscore the therapeutic potential of ICB in treating PPP2R2A-deficient NSCLC and suggest that PPP2R2A deficiency could serve as a biomarker for guiding ICB-based therapies.

## Abstract 2

PPP2R2A insufficiency in non-small cell lung cancer enhances PD-L1 blockade efficacy via cGAS-STING-IFN activation, suggesting PPP2R2A deficiency as a biomarker for immune checkpoint blockade response

## Introduction

Lung cancer is the leading cause of cancer-related deaths in the United States, with non–small cell lung cancer (NSCLC) representing approximately 80% of cases. Programmed cell death ligand 1 (PD-L1), an immune checkpoint protein, binds to programmed cell death protein 1 (PD-1) on T cells, causing dysfunction; PD-1/PD-L1 antibodies block this interaction to restore antitumor activity. FDA-approved immune checkpoint blockade (ICB) therapies for NSCLC can be used alone or in combination (1). Yet only a subset of patients respond, highlighting the need for biomarker-guided approaches to improve outcomes.

PP2A is a heterotrimeric serine/threonine phosphatase consisting of a catalytic subunit (C), a scaffold subunit (A), and 1 of 18 structurally distinct regulatory B subunits (2, 3). These subunits combine to form more than 180 holoenzymes, each with unique substrate specificity and cellular localization (4), enabling PP2A to regulate diverse processes, including signal transduction, cell cycle progression, DNA replication, gene transcription, protein translation, and DNA repair response (3–7). PP2A B55α, a regulatory B subunit of PP2A, is underexpressed in greater than 40% of cases of NSCLC, due to loss of heterozygosity (LOH) of *PPP2R2A* (8), the gene encoding this protein. Reduced B55α expression correlates with poor prognosis. Thus, targeting *PPP2R2A*-deficient lung cancer is an unmet medical need (8–11). Previous studies, including our own, have shown that *PPP2R2A* knockdown (KD) enhances the sensitivity of NSCLC and ovarian cancer to inhibitors targeting the cell cycle checkpoint proteins CHK1 or ATR, through its impact on the replication stress (RS) induced by oncogenic c-Myc (10, 12). In addition, PPP2R2A deficiency also increases the sensitivity of tumors to PARP inhibitors by phosphorylation-mediated regulation of ataxia-telangiectasia mutated (8). Interestingly, PPP2R2A deficiency has been implicated in resistance to cisplatin (12, 13) and MEK inhibitors (14), highlighting its complex roles in cancer treatment. Despite these findings, the impact of *PPP2R2A* heterozygosity on ICB-based therapies remains unexplored.

The efficacy of ICB can be influenced by numerous factors. Cytosolic DNA sensing through the cyclic GMP-AMP synthase-stimulator of interferon genes (cGAS-STING) pathway plays a crucial role in PD-1– or PD-L1 blockade-based immunotherapy by promoting type I IFN expression (15, 16). Type I IFNs are a family of monomeric cytokines consisting of 14 IFN-α subtypes, including IFN-β, IFN-ε, IFN-κ, and IFN-ω. IFN-α and IFN-β are the 2 major IFNs that have been extensively studied, whereas the functions of IFN-ε, IFN-κ, and IFN-ω remain poorly understood. Beyond bacterial and viral infections, this pathway can also be activated by cytosolic DNA from damaged mitochondria, certain parasites, cancer cells with genomic instability, and self-DNA released from damaged cells (17). Our previous study demonstrated that *PPP2R2A* KD increased RS and DNA double-strand breaks (DSBs) (10, 12). Because RS is a major source of cytosolic DNA-mediated triggering of the cGAS-STING-IFN pathway and improves ICB efficacy in NSCLC (18, 19), it is plausible that *PPP2R2A* deficiency in tumor cells affects ICB outcomes by activation of the cGAS-STING-IFNs axis and the subsequent modulation of the tumor immune microenvironment (TME). However, this hypothesis remains untested.

Here, using both human NSCLC and mouse tumor cell line models, we demonstrate that *PPP2R2A* deficiency, achieved through either KD or heterozygous KO) (*PPP2R2A^+/–^* in NSCLC, results in cytosolic DNA accumulation and elevated PD-L1 expression via inhibitory phosphorylation of GSK-3β and a STING-dependent mechanism. Consistent with our in vitro results, which show that *PPP2R2A* deficiency induces cytosolic DNA and activates the cGAS-STING-IFN pathway, we found *Ppp2r2a^+/–^* enhances the efficacy of PD-L1 antibody treatment in vivo in a syngeneic mouse model of NSCLC. By immune phenotyping, we reveal that *Ppp2r2a^+/–^* tumor cells alter the immune cell composition within the TME, characterized by an increase in NK cells and a reduction in Treg infiltration, compared with tumors with intact *Ppp2r2a*. We further found that after anti–PD-L1 antibody treatment, tumors derived from *Ppp2r2a^+/–^* cells exhibited increased infiltration of CD8^+^ T cells. In line with this observation, the enhanced antitumor effects of PD-L1 blockade in *Ppp2r2a^+/–^* tumors were dependent on CD4^+^ T cells, CD8^+^ T cells, and NK cells. Furthermore, abrogation of type I IFN signaling through IFNAR1 blockade, either by systemic antibody treatment or specific genetic deletion in Treg cells, reversed the antitumor efficacy of PD-L1 blockade in *Ppp2r2a^+/–^* tumors. In summary, our findings reveal that *PPP2R2A*-deficient NSCLC cells, via activation of the cGAS-STING-IFN pathway, are more susceptible to anti–PD-L1–based therapy. Thus, PPP2R2A status in tumors may serve as a novel biomarker to guide patient selection for ICB-based therapies in the treatment of NSCLC.

## Results

### PPP2R2A deficiency leads to the accumulation of cytosolic DNA, activating the cGAS-STING pathway in mouse and human NSCLC cells.

Results from our previous study suggest that *PPP2R2A* KD in the human NSCLC cell lines A549 and H1299 leads to increased RS and DSBs (10). To further determine the potential pathways affected by PPP2R2A deficiency, we conducted bulk RNA-Seq analysis in the A549 cells with or without PPP2R2A KD (12) (Supplemental Figure 1A; supplemental material available online with this article; https://doi.org/10.1172/JCI193354DS1). GSEA analysis of this dataset suggested that IFN-α response, inflammatory response, and IFN-γ response were enriched in *PPP2R2A* KD cells (Supplemental Figure 1B).

Given that the cGAS-STING-IFN pathway is activated by small DNA fragments in the cytoplasm induced by RS (20) and is important for the activation of inflammatory and IFNs signaling, we hypothesized that *PPP2R2A* deficiency activates the cGAS-STING-IFN pathway via cytosolic DNA. To test our hypothesis, we generated *PPP2R2A* heterozygote (*PPP2R2A^+/–^*) cell lines using both mouse and human lung cancer models, because The Cancer Genome Atlas (TCGA) analysis shows that 53.3% of NSCLC cases have shallow PPP2R2A deletion, whereas approximately 4% exhibit deep deletion, and PPP2R2A loss correlates with reduced expression (12). In addition, *PPP2R2A* shallow deletion is associated with an increased tumor mutational burden (TMB) (Supplemental Figure 2, A–C) that is often associated with genomic instability because of damaged DNA and RS. Furthermore, patients with NSCLC with shallow *PPP2R2A* deletions have poorer prognoses (Supplemental Figure 2, D and E).

It is worth noting that the analysis of TCGA-based data suggests TMB is not associated with patient survival (i.e., disease-specific survival and disease-free interval). After we controlled for the level of TMB in the Cox proportional hazard regression model, the association between PPP2R2A shallow deletion and patient survival in Supplemental Figure 2 was slightly changed, but the difference did not reach statistical significance (for disease-free interval and disease-specific survival, *P* = 0.15 and 0.06, respectively, after controlling for TMB level) (Supplemental Table 1). Therefore, PPP2R2A deficiency, instead of TMB, might directly drive poor prognosis.

**Figure 1:**
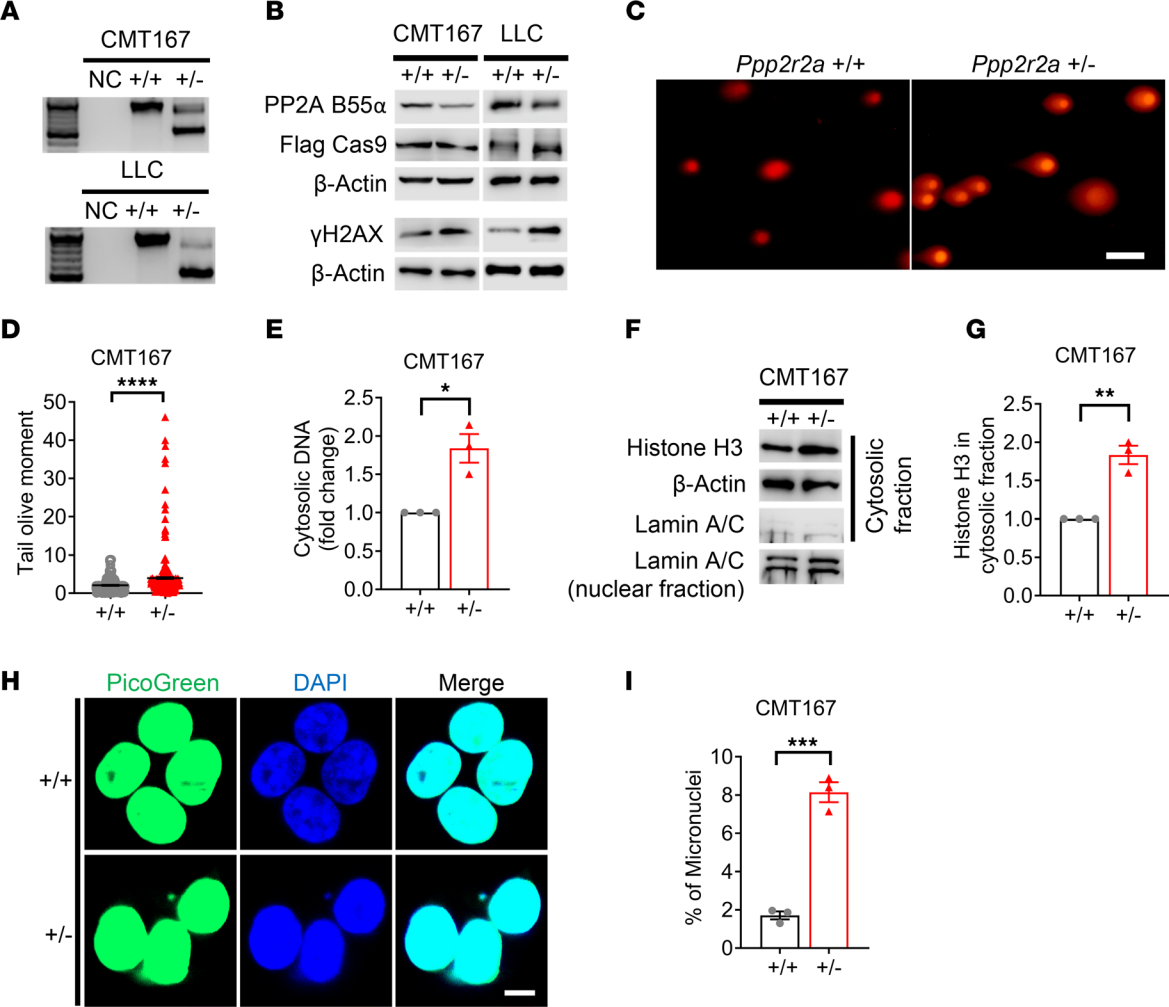
Monoallelic PPP2R2A KO leads to cytosolic DNA accumulation. (**A** and **B**) Genotyping and representative Western blot of *Ppp2r2a*^+/+^ and *Ppp2r2a^+/–^* CMT167 and LLC cells. (**C** and **D**) Neutral comet assays showing DNA DSBs. Data are presented as mean ± SEM from 3 biological repeats (*n =* 300) in **D**. (**E–I**) Quantification of cytosolic DNA, cytosolic histone H3, and MN in CMT167 cells. Data are presented as mean ± SEM (*n =* 3). *P < 0.05, **P < 0.01, ***P < 0.001, ****P < 0.0001 by Student’s *t* test. Scale bars: 200 μm in **C** and 30 μm in **H**.

We next generated *PPP2R2A^+/–^* cells by targeting exon 3 of the gene, using a CRISPR/Cas9 approach. PCR primers flanking exon 3 were used for characterizing the gene editing results in the transduced and selected cells. Deletion of exon 3 of both mouse *Ppp2r2a* and human *PPP2R2A* resulted in the expected frameshifts, leading to the degradation of full-length mRNA by nonsense-mediated mRNA decay (Supplemental Figure 3). For mouse cells, genotyping results showed that single clones of *Ppp2r2a* heterozygous KO were successfully generated in the mouse lung tumor cell lines CMT167 and LLC (Figure 1A). However, no clones with homozygous depletion (*Ppp2r2a^–/–^*) were generated, suggesting that Ppp2r2a is essential for mouse lung cancer cell survival, which is consistent with a previous report that complete loss of *Ppp2r2a* results in embryonic cell death (21). By immunoblotting, we further confirmed the successful heterozygous KO of *Ppp2r2a* and revealed an increase in the levels of the DSB marker γH2AX in the 2 mouse lung cancer cells (Figure 1B). Using neutral comet assays, we further observed that the degree of DSBs was significantly elevated in CMT167 *Ppp2r2a^+/–^* cells. Representative results of the comet assay are shown in Figure 1C, with quantifications of Olive Tail presented in Figure 1D. Furthermore, we measured the levels of cytosolic DNA using a SpectraMax Quant AccuClear Nano dsDNA Assay Kit and found greater accumulation of DNA in the cytoplasm in the heterozygous KO of *Ppp2r2a* compared with wild-type controls (Figure 1E). This finding was further verified by an upregulation of histone H3 protein in the cytoplastic fraction (Figure 1, F and G). We next stained for cytosolic DNA by PicoGreen and DAPI in *Ppp2r2a^+/–^* CMT167 cells and found a greater percentage of cells with micronuclei (MN), which are an important source of cell-intrinsic immunostimulatory DNA via promotion of cGAS-STING-IFN (22), compared with *Ppp2r2a^+/+^* cells (Figure 1, H and I).

We also generated human NSCLC cells with heterozygous (*PPP2R2A^+/–^*) and homogeneous (*PPP2R2A^–/–^*) deletion (Supplemental Figure 4, A and B), which is different than what we observed in mouse cells where only *Ppp2r2a^+/–^* cells were able to be generated. In these *PPP2R2A* KO A549 cells, there was a markedly greater degree of DSBs compared with control cells (Supplemental Figure 4, C and D). Furthermore, we observed enhanced levels of cytosolic DNA and MN in both the heterozygous and homozygous deletion A549 cells compared with the control cells (Supplemental Figure 4, E–G).

To validate the results observed in cells of the *PPP2R2A* KO clones, we conducted additional validations using pooled population cells with *PPP2R2A* KD in both mouse and human cells. In mouse cells, stable KD of *Ppp2r2a* in CMT167 and LLC cells led to increased RS, compared with controls (Supplemental Figure 5A). In CMT167 cells with *Ppp2r2a* KD, we observed a significant increase in DSBs (Supplemental Figure 5, B and C) and cytosolic DNA accumulation, compared with controls (Supplemental Figure 5D). Additionally, histone H3 was notably upregulated in the cytoplasmic fraction (Supplemental Figure 5, E and F), and we observed an increased percentage of cells with MN (Supplemental Figure 5, G and H). Similarly, *PPP2R2A* KD in our 2 human lung cancer cell lines resulted in elevation of RS (Supplemental Figure 6A), as we previously reported (10). In A549 cells with low PPP2R2A expression (10), we noted an increase in DSBs (Supplemental Figure 6, B and C), higher cytosolic DNA levels (Supplemental Figure 6D), and a greater percentage of cells with MN (Supplemental Figure 6, E and F). Additionally, we further examined cytosolic DNA levels in 4 NSCLC cell lines: A549, H838, SK-MES-1, and H1437. Among them, SK-MES-1 and H1437 exhibited higher levels of cytosolic DNA (Supplemental Figure 7, A and B), which correlates with their lower PPP2R2A expression resulting from LOH (8). Therefore, PPP2R2A deficiency causes cytosolic DNA accumulation, activating the cGAS-STING pathway in mouse and human NSCLC cells

### PPP2R2A /Pppp2r2a downregulation activates the cGAS-STING pathway to trigger type I IFN production in lung cancer cells.

**Figure 2:**
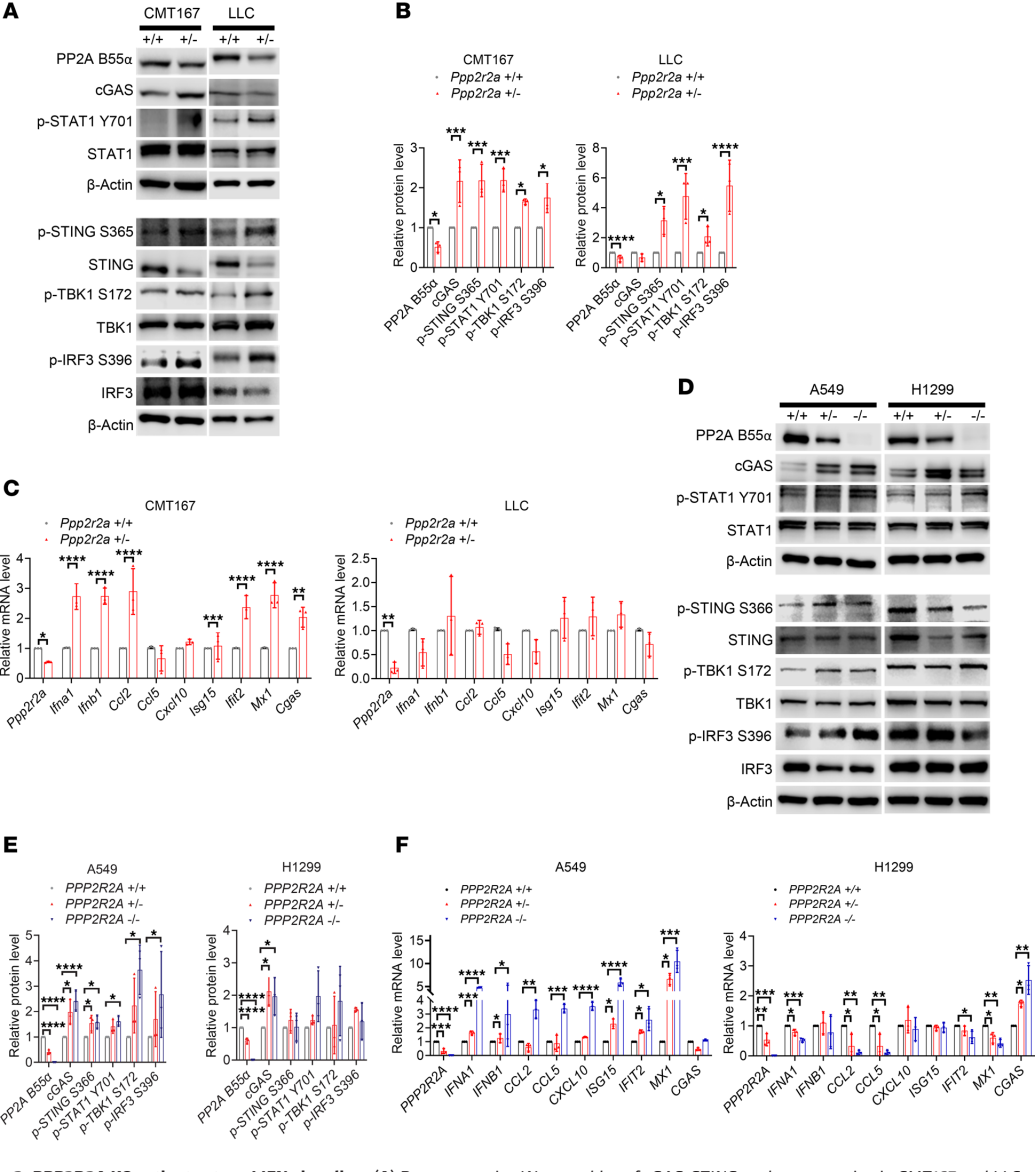
PPP2R2A KO activates type I IFN signaling. (**A**) Representative Western blot of cGAS-STING pathway proteins in CMT167 and LLC cells. (**B**) Quantification of protein levels from **A**. (**C**) qPCR of cGAS-STING– and IFN-related genes in CMT167 and LLC cells. (**D**) Representative Western blot of cGAS-STING pathway proteins in A549 and H1299 cells. (**E**) Quantification of protein levels from **D**. (**F**) qPCR of cGAS-STING– and IFN-related genes in A549 and H1299 cells. Data are presented as mean ± SEM (*n =* 3). *P < 0.05, **P < 0.01, ***P < 0.001, ****P < 0.0001 by Student’s *t* test or 1-way ANOVA with Bonferroni post hoc test.

To investigate whether the accumulation of cytosolic DNA in cells with PPP2R2A deficiency activates the cGAS-STING-IFN pathway, we first measured the phosphorylation (p) status of the key components of the cGAS-STING pathway. We found higher p-STING, p-STAT, p-TBK, and p-IRF3 levels in *Ppp2r2a^+/–^* CMT167 and LLC cells than in *Ppp2r2a^+/+^* cells (Figure 2, A and B). In line with these results, we found significant upregulation of IFN-α, IFN-β, and several other downstream factors of IFNs assessed by quantitative PCR (qPCR) in CMT167 (Figure 2C). In contrast, PPP2R2A deficiency in LLC cells resulted in no obvious increase in type I IFN. Similarly, we observed increased expression levels of key markers of cGAS-STING pathway activation in *PPP2R2A^+/–^* and *PPP2R2A^–/–^* A549 and H1299 cells (Figure 2, D and E). Also, we found an elevation of type I IFN expression in A549 *PPP2R2A^+/–^* and *PPP2R2A^–/–^* but not in H1299 *PPP2R2A^+/–^* and *PPP2R2A^–/–^* cells compared with their respective controls (Figure 2F).

To further confirm these observations in cell clones with *Pppp2r2a* KO, we next investigated the effects of stable *Ppp2r2a* KD on cGAS-STING-IFN pathway activation in CMT167 and LLC cells. We found that lower expression of Ppp2r2a achieved by 2 different shRNAs resulted in higher levels of cGAS-STING pathway proteins in both CMT167 and LLC cells, along with elevated cGAS and STING expression, as compared with control cells (Supplemental Figure 8, A and B). These results align with those of previous reports showing that both STING and cGAS are downstream factors of cGAS-STING (23, 24). Notably, although we observed increased expression of type I IFN in CMT167 *Ppp2r2a* KD cells, almost none of the tested IFNs and downstream factors, except for *Mx1*, were higher in LLC *Ppp2r2a* KD cells compared with control cells (Supplemental Figure 8C). Thus, the activation of the cGAS-STING pathway may not necessarily lead to the production of IFN signaling. Simply detecting the phosphorylation status of downstream cGAS-STING factors without assessing IFN generation may lead to an underestimation of the complete activation of the cGAS-STING-IFN pathway.

Like mouse CMT167 cells, *PPP2R2A* KD in the human cell line A549 resulted in activation of the cGAS-STING (Supplemental Figure 8, D and E) and type I IFN pathways and the latter’s downstream factor expression (Supplemental Figure 8F). Furthermore, by ELISA, we found that both *PPP2R2A/Pppp2r2a* KD and KO significantly increased the secretion of IFN-β and/or IFN-α in human A549 cells (Supplemental Figure 9, A and B) and mouse CMT167 cells (Supplemental Figure 9, C–F), as measured in in vitro culture supernatants. Consistent with these results, we found elevated levels of IFN-β and/or IFN-α in the serum of *Ppp2r2a^+/−^* tumor-bearing mice (Supplemental Figure 9, G and H).

Taken together, our results suggest PPP2R2A deficiency induces RS and cytosolic DNA accumulation, which subsequently activates the cGAS-STING-IFNs pathway, suggesting a potential mechanistic link between genetic loss of *PPP2R2A*, cytosolic DNA accumulation and immune signaling pathway activation. However, it is context dependent: no significant increase in type I IFN was detected in PPP2R2A-deficient LLC and H1299 cells.

### PPP2R2A deficiency results in increased PD-L1 expression via GSK-3β– and cGAS-STING–dependent mechanisms.

PD-L1 (B7-H1) is a 33 kDa transmembrane protein, but in tumor cells it is typically detected at approximately 45 kDa due to glycosylation, which stabilizes PD-L1 by preventing GSK3β-mediated phosphorylation and degradation (25). To assess PD-L1 glycosylation, we treated cells with PNGase F, which reduced the approximately 45 kDa band to 33 kDa, confirming that the predominant form of PD-L1 in our cancer cell lines is glycosylated (Supplemental Figure 10, A–D).

**Figure 3:**
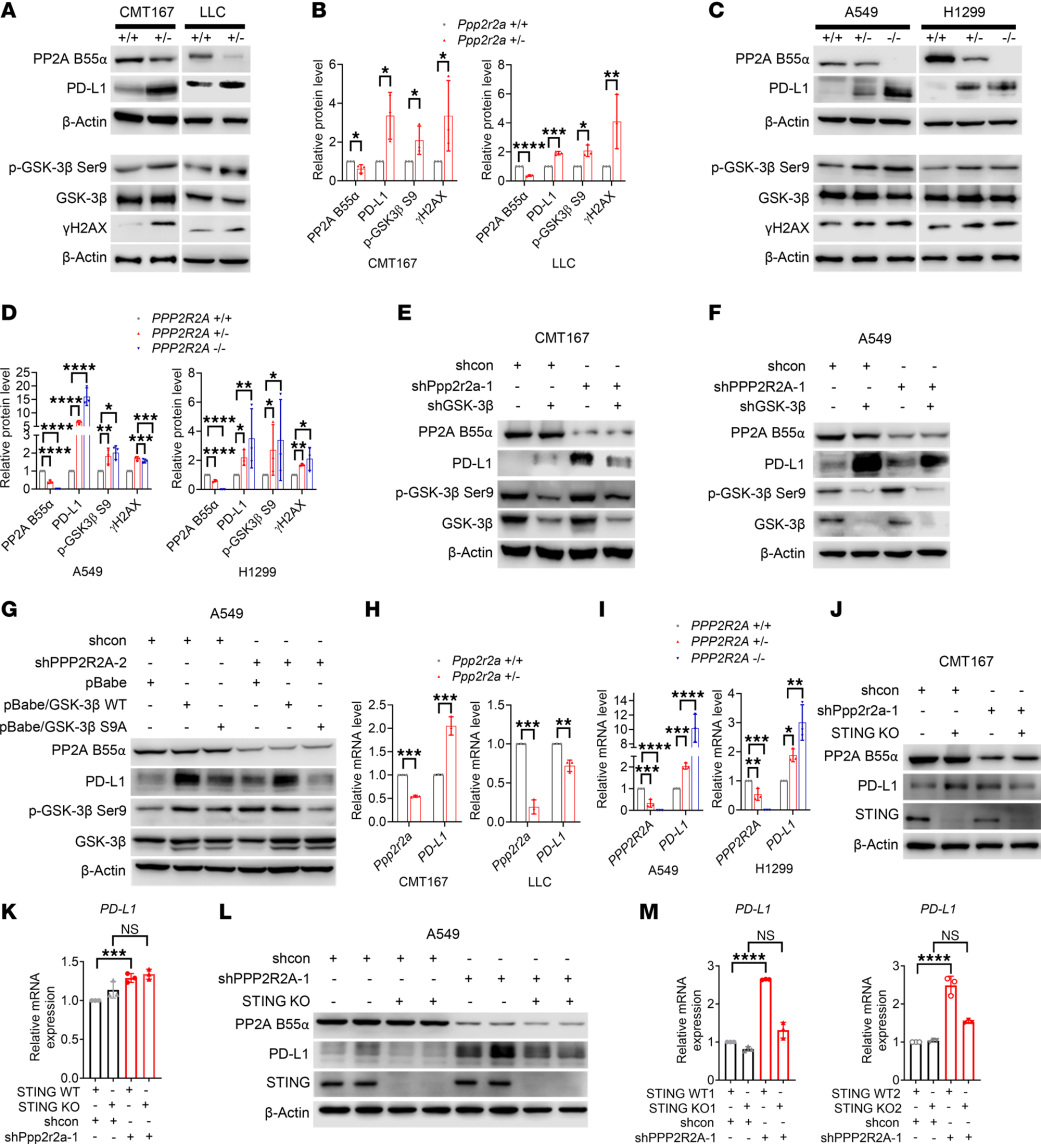
PPP2R2A deficiency increases PD-L1 expression. (**A–D**) Representative Western blot analysis showing increased PD-L1 protein levels in mouse (CMT167, LLC) and human (A549, H1299) cells after PPP2R2A KO, with quantification from biological replicates. (**E–G**) PD-L1 induction by PPP2R2A KD is dependent on GSK-3β phosphorylation. (**H–I**) qPCR of PD-L1 mRNA in human and mouse cells. (**J–M**) PD-L1 upregulation by PPP2R2A KD requires STING in CMT167 and A549 cells. *P < 0.05, **P < 0.01, ***P < 0.001, ****P < 0.0001. Data are reported as mean ± SD (*n =* 3). (**B** and **H**) A Student *t* test was used for the data analysis. (**D**, **I**, **K** and **M**) Statistical analysis was conducted using 1-way ANOVA followed by Bonferroni post hoc test for multiple comparisons. shcon, short hairpin RNA control.

Findings in our previous report suggested that *PPP2R2A* KO leads to an increase in the inhibitory phosphorylation of GSK-3β (p-GSK-3β-ser9) (12), and here we also found that PPP2R2A deficiency leads to cGAS-STING pathway activation (Figure 2). Thus, we next determined the status of PD-L1 expression in *PPP2R2A^+/–^* cells and also investigated whether both mechanisms (elevations of p-GSK-3β-ser9 and activation of cGAS-STING) are involved in regulation of PPP2R2A deficiency-induced PD-L1 expression. In heterozygous CMT167 and LLC cells, we found higher PD-L1 protein expression, along with higher levels of p-GSK-3β-ser9, especially in CMT167 heterozygous cells, compared with wild-type controls (Figure 3, A and B). Additionally, in pooled *Ppp2r2a* KD populations, we found higher PD-L1 and p-GSK-3β-ser9 levels in both CMT167 and LLC mutant cells, compared with controls (Supplemental Figure 11, A and B).

Supporting these mouse data, we found similar results in human cells. In particular, the protein levels of PD-L1 were higher in *PPP2R2A^+/–^* and *PPP2R2A^–/–^* A549 and H1299 cells than in their respective controls (Figure 3, C and D). The levels of p-GSK-3β-ser9 were also higher in the heterozygous and homozygous *PPP2R2A* KO cells compared with the control cells (Figure 3, C and D). We found that PPP2R2A deficiency leads to an upregulation of PD-L1. PD-L1 protein expression, as well as p-GSK-3β-ser9 were elevated at the protein level in *PPP2R2A* KD A549 and H1299 cells (Supplemental Figure 11, D and E). PD-L1 mRNA levels were also increased in *PPP2R2A* KD A549 cells (Supplemental Figure 11F). Together, these results suggest PPP2R2A deficiency leads to increased PD-L1 expression, which is associated with increased p-GSK-3β-ser9 expression. Thus, GSK-3β, especially p-GSK-3β-ser9, may be involved in the PPP2R2A deficiency-induced elevation of PD-L1 expression.

To directly test this hypothesis, we next determined if phosphorylation of GSK-3β is required for PPP2R2A deficiency–induced PD-L1 expression. Stable KD of the gene encoding GSK-3β reduced the Ppp2r2a deficiency–induced upregulation of PD-L1 in CMT167 cells (Figure 3E), with the same effect observed in A549 cells with GSK-3β KD (Figure 3F). In addition, in A549 cells, stable overexpression of the GSK-3β-S9A phosphorylation-defective mutant increased PD-L1 protein levels, whereas *PPP2R2A* KD had no further effect on PD-L1 expression in these cells (Figure 3G). Moreover, the expression of exogenous PPP2R2A in H1437 cells, which have PPP2R2A deficiency due to LOH, led to lower levels of p-GSK-3β-ser9 (Supplemental Figure 11, G and H). Furthermore, transient KD of GSK3β in both A549 *PPP2R2A^+/–^* and *PPP2R2A^–/–^* (Supplemental Figure 12, A and B) and CMT167 *Ppp2r2a^+/–^* (Supplemental Figure 12, C and D) cell lines resulted in a reduction of PPP2R2A deficiency–induced PD-L1 expression, compared with cells with intact GSK-3β. Together, these results support the notion that GSK-3β (p-GSK-3β-ser9) is required for PPP2R2A deficiency–induced PD-L1 upregulation.

Additionally, PPP2R2A deficiency–induced PD-L1 can also be regulated at the transcriptional level by the activation of cGAS-STING-type I IFN pathway (26–28). Activation of the cGAS-STING pathway upregulates PD-L1 expression (28). Given the increased cGAS-STING pathway activation in cells with PPP2R2A deficiency (Figure 2), we first determined how PPP2R2A deficiency affects PD-L1 expression at the mRNA level. We found that *Ppp2r2a* heterozygosity led to the increased expression of PD-L1 mRNA level in CMT 167 cells; however, the increased expression of PD-L1 mRNA level was not observed in LLC cells (Figure 3H and Supplemental Figure 11C), suggesting the upregulation of PD-L1 in cells with PPP2R2A deficiency could be due to protein and/or transcriptional regulation. Similarly, PD-L1 expression was elevated at the mRNA level in *PPP2R2A* KO and KD A549 and H1299 cells, though it was much less significant was observed in H1299 cells than in A549 cells (Figure 3I and Supplemental Figure 11F). In LLC cells, transcriptional regulation may not be involved.

To directly test if the cGAS-STING pathway is involved in PD-L1 expression, we next investigated whether *STING* KO diminishes PD-L1 expression in cells with low PPP2R2A or Ppp2r2a expression. We generated a cell clone with *STING* KO using CRISPR/cas9. We found that PD-L1 protein upregulation triggered by *Ppp2r2a* KD was decreased in *Sting* KO CMT167 cells (Figure 3J and Supplemental Figure 13A). The KD of *Ppp2r2a* failed to upregulate PD-L1 mRNA in *Sting*-KO CMT167 cells (Figure 3K and Supplemental Figure 13B). Similar results were also observed in *STING* KO A549 cells (Figure 3, L and M, and Supplemental Figure 13, C–E). Therefore, it is likely that the cGAS-STING pathway–mediated PD-L1 expression also contributes to the increased PDL-1 expression observed in PPP2R2A-deficient cells. Flow cytometric analysis further revealed that cell surface PD-L1 expression was elevated in *Ppp2r2a^+/−^* CMT167 cells in vitro (Supplemental Figure 14, A–C). Together, these data suggest PPP2R2A deficiency leads to increased PD-L1 expression at the protein and/or mRNA levels. The inactivation of GSK-3β, along with the activation of the cGAS-STING-type I IFN pathway, could contribute to the increase in PD-L1 expression induced by PPP2R2A deficiency.

In summary, PPP2R2A deficiency induces PD-L1 expression through 2 mechanisms: inhibitory phosphorylation of GSK3β (Ser9), which increases PD-L1 at the protein level across all tested cells, and activation of the cGAS–STING pathway, which upregulates PD-L1 at the mRNA level in a cell context–dependent manner. For example, both mechanisms operate in CMT167 and A549 cells, whereas only GSK3β (Ser9) acts in LLC cells. Despite these differences, PPP2R2A deficiency consistently increases PD-L1 expression.

### Ppp2r2a deficiency enhances PD-L1 blockade efficacy.

**Figure 4:**
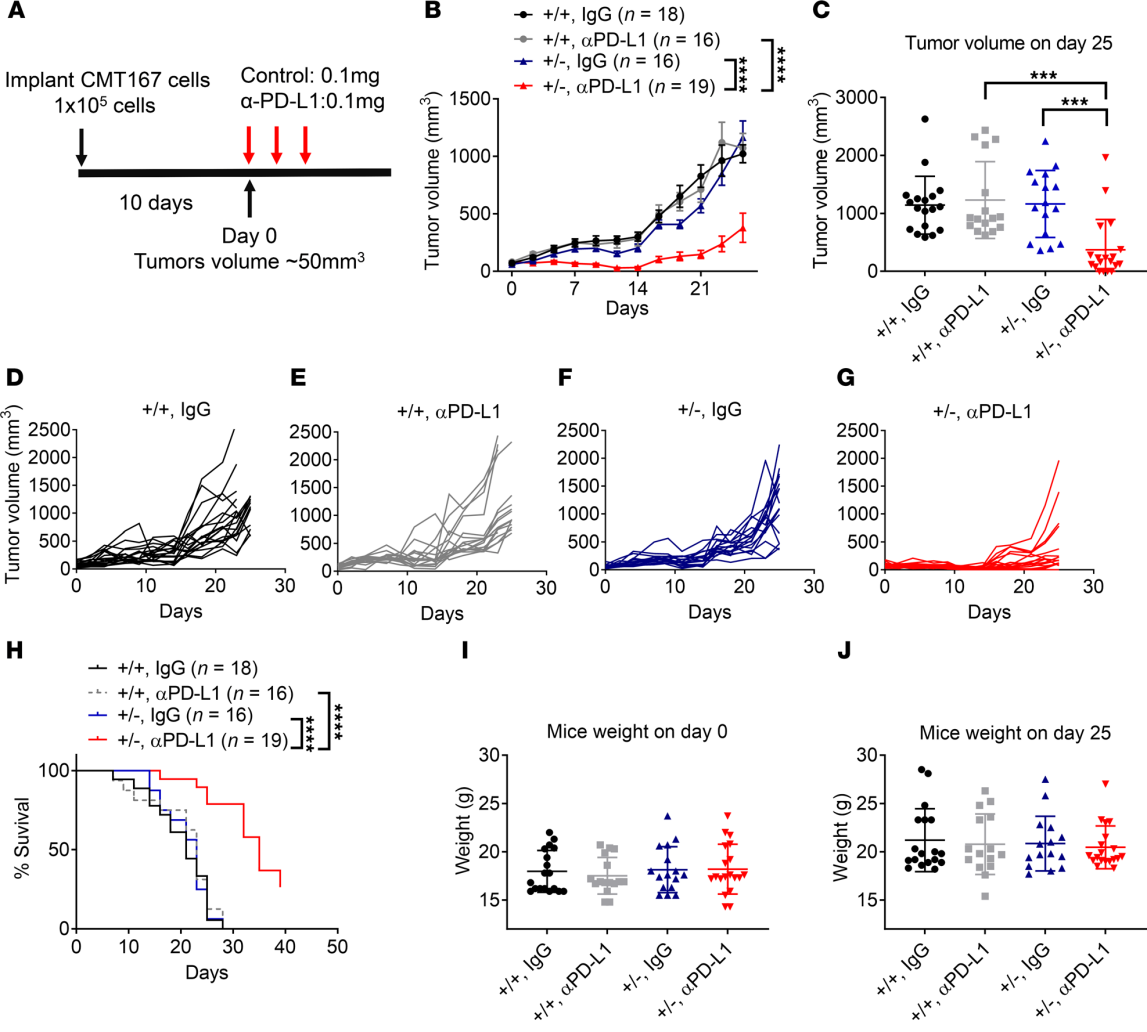
Ppp2r2a heterozygosity sensitizes CMT167 tumors to PD-L1 blockade in vivo. (**A**) Treatment schedule in mice bearing Ppp2r2a^+^/^+^ or *Ppp2r2a^+/–^* tumors. (**B–G**) Tumor growth curves and individual tumor volumes with control or anti–PD-L1 antibody. (**H**) Kaplan–Meier survival analysis. ****, *P* < 0.0001, Kaplan-Meier analysis was used for overall survival. (**I–J**) Mouse body weights on day 0 and day 25. Data are presented as mean ± SEM. ***P < 0.001, ****P < 0.0001 by 1- or 2-way ANOVA with Bonferroni post hoc test.

We next examined the effect of Ppp2r2a heterozygosity on tumor growth and responses to an antibody against PD-L1 in syngeneic host C57BL/6J mice, from which CMT167 and LLC cells originated. We first treated mice bearing *Ppp2r2a^+/+^* and *Ppp2r2a^+/–^* CMT167 tumors with control antibodies or anti–PD-L1 antibodies (100 μg, 3 doses) (Figure 4A). In mice with control tumors (*Ppp2r2a^+/+^*), we found no significant antitumor response following PD-L1 blockade. In addition, *Ppp2r2a* heterozygous KO alone did not affect tumor growth in the presence of control antibodies (Figure 4B). However, within 1 week, we did observe substantial tumor regression in the mice bearing *Ppp2r2a^+/–^* tumors that were treated with PD-L1 blockade. They remained the lowest among the 4 groups (Figure 4, B and C). The growth curves of each group are demonstrated in Figure 4, D–G. Additionally, PD-L1 blockade significantly improved the survival of mice bearing *Ppp2r2a* heterozygous KO CMT167 cells (Figure 4H). The dosage of control and anti–PD-L1 antibodies did not affect the body weight of C57BL/6 mice. Body weights for each group, recorded on day 0 and at the cutoff date, are shown in Figure 4, I and J. Thus, we conclude that *Ppp2r2* heterozygous depletion potentiates the efficacy of PD-L1 blockade in vivo in our CMT167 cell line model of NSCLC.

It is worth noting that, consistent with our observation that PPP2R2A deficiency leads to increased PD-L1 expression in vitro (Figure 3, Supplemental Figure 11, and Supplemental Figure 14, A–C), flow cytometric analysis further revealed that cell surface PD-L1 expression was elevated in *Ppp2r2a^+/−^* CMT167 tumors in vivo (Supplemental Figure 14, D and E).

We next determined the impact of *Ppp2r2a* heterozygosity on PD-L1 antibody treatment in LLC cells, a cell line showing no obvious upregulation of IFNs (Supplemental Figure 15A). *Ppp2r2a* heterozygous KO significantly suppressed LLC tumor growth (Supplemental Figure 15, B and C); however, PD-L1 blockade failed to sensitize *Ppp2r2a^+/–^* cells to the effects of the PD-L1 antibody, which is similar to what we saw in *Ppp2r2a^+/+^* cells (Supplemental Figure 15, B, and D–G). Expectedly, given these results, PD-L1 blockade did not prolong the survival of the mice bearing LLC tumors (Supplemental Figure 15H). Body weight data for each group are presented in Supplemental Figure 15, I and J. This result aligns well with the failure to activate IFN signaling despite increased RS in LLC cells (Figure 2C and Supplemental Figure 8C), further supporting our hypothesis that cGAS-STING-IFN signaling plays a crucial role in the response to ICB.

Thus, our results suggest that cGAS-STING-IFN activation, instead of merely cGAS-STING activation, is important to the antitumor immune response. In LLC cells, although activation of the cGAS-STING pathway was evidenced by increased phosphorylation of key signaling components (Figure 2, A and B, and Supplemental Figure 8, A and B), the expression of the tested type I IFN-related genes did not appear to increase, for reasons that are not yet clear (Figure 2C and Supplemental Figure 8C). Consequently, PPP2R2A deficiency–induced PD-L1 mRNA levels in these cells do not increase to the same extent as observed in CMT167 cells. Therefore, in CMT167 cells, PPP2R2A deficiency induces cGAS-STING-IFN activation and increases sensitivity to PD-L1 antibody, whereas in LLC cells, cGAS-STING activation occurs without a substantial increase in type I IFN and dramatic impact on PD-L1 antibody sensitivity.

Lastly, in support of the result showing that PPP2R2A deficiency or low expression increases PD-L1 expression and enhances the efficacy of anti–PD-L1 ICB (Figures 3 and 4), we found that patients with a low PPP2R2A/PD-L1 ratio in their cancer had improved overall survival when treated with immune checkpoint inhibitors, including antibodies targeting PD-L1 (Supplemental Figure 16A), PD1 (Supplemental Figure 16B), and CTLA4 (Supplemental Figure 16C). Similarly, by progression-free survival analysis, we found that patients with a low PPP2R2A/PD-L1 ratio demonstrated enhanced therapeutic efficacy to PD-L1 antibodies (Supplemental Figure 16D), PD-1 antibodies (Supplemental Figure 14E), and CTLA4 antibodies (Supplemental Figure 16F). Together, these results indicate that PPP2R2A deficiency or low expression, particularly accompanied by increased PD-L1 expression, enhances the response to ICB.

### Deficiency in Ppp2r2a in tumor cells enhances the efficacy of PD-L1 blockade by modifying the TME via increasing NK cell infiltration and reducing Treg infiltration.

**Figure 5:**
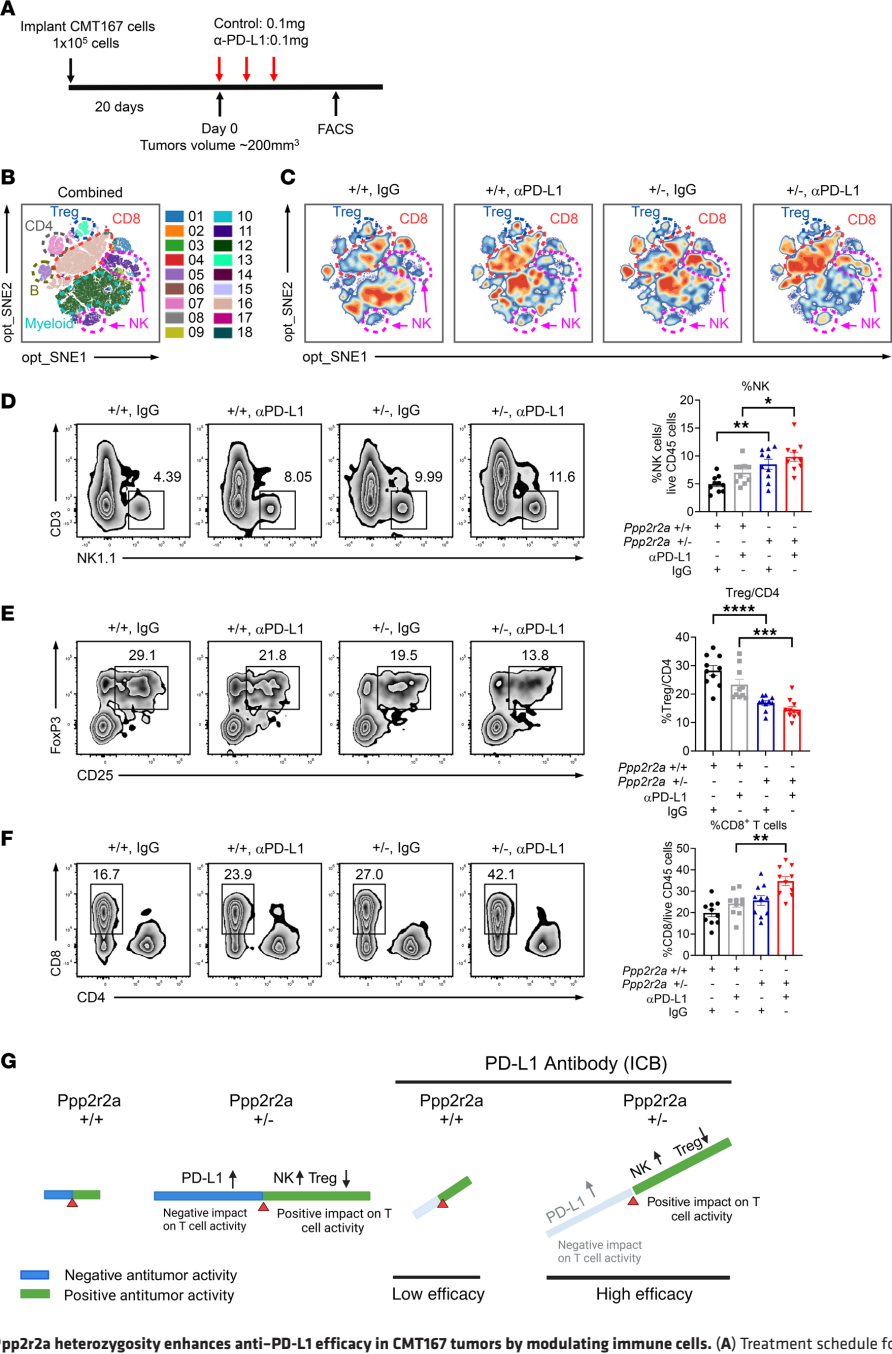
Ppp2r2a heterozygosity enhances anti–PD-L1 efficacy in CMT167 tumors by modulating immune cells. (**A**) Treatment schedule for control antibody or anti–PD-L1 therapy followed by immune profiling. (**B–C**) Flow cytometry of CD45^+^ tumor-infiltrating immune cells with opt-SNE and FlowSOM clustering. (**D–F**) Representative plots and quantification of NK, Treg, CD4^+^, and CD8^+^ T cell populations. Data are presented as mean ± SEM (*n =* 10). *P < 0.05, **P < 0.01, ***P < 0.001, ****P < 0.0001 by 1-way ANOVA with Bonferroni post hoc test. (**G**) Schematic summary of TME reprogramming in PPP2R2A-deficient NSCLC under PD-L1 blockade.

We next determined the cellular mechanisms underlying the enhanced antitumor efficacy for the PD-L1 blockade in *Ppp2r2a^+/–^* CMT167 tumors via multispectral flow cytometry according to the regimen described in Figure 5A. One day after administering the third antibody dose, tumors were harvested and tumor infiltrating lymphocytes were isolated using Percoll gradient separation, followed by extensive phenotypic analyses using multispectral flow cytometry (CyTek) with a comprehensive immune panel comprising 26 markers to define myeloid and lymphoid lineages. These antibodies included markers to identify T cells, B cells, DCs, and macrophages. The panel included antibodies against CD45, CD3, CD4, CD8, B220, CD11b, CD11c, NK1.1, Ly6C, F4/80, MHC class II, PD-1, CD24, CD103, Foxp3, CD64, CD206, Arg1, CD25, PD-L1, TCRb, CD38, CD172, XCR1, Ly6G, and CD19. The gating strategy used in this research is shown in Supplemental Figure 17.

To visualize tumor-infiltrating immune cell populations, we gated live CD45^+^ immune cells, performed opt_SNE dimension reduction and FlowSOM clustering, and we annotated key populations (Figure 5B). To compare population dynamics across the experimental groups, we generated contour plots and observed increased accumulation of NK cells along with decreased Treg accumulation in *Ppp2r2a^+/–^* tumor samples (Figure 5C). To validate our observations, we generated 2D flow cytometry plots to identify NK cells (CD3^-^ NK1.1^+^), Tregs (FoxP3^+^ CD25^+^), and CD8^+^ T cells (CD8^+^ CD4^–^). Statistical analysis and representative figures indicate that *Ppp2r2a^+/–^* tumors were associated with greater NK cell infiltration (Figure 5D) and less Treg infiltration (Figure 5E) even before PD-L1 antibody treatment. These results suggest *Ppp2r2a* heterozygosity in tumors reshapes the TME. PD-L1 antibody treatment caused a significant increase in the proportion of cytotoxic CD8^+^ cells among *Ppp2r2a^+/–^* cells (Figure 5F), compared with tumors with intact *Ppp2r2a*. Given that *PPp2r2a* heterozygosity also leads to increased PD-L1 expression, which has a potential to suppress T cell activity, it is most likely that *Ppp2r2a* heterozygosity causes a double-edged impact on the TME. Namely, on 1 hand, it has a positive impact on antitumor activity by increasing NK cell infiltration and decreasing Treg infiltration, but on the other hand, it has a negative impact on antitumor activity by triggering increased PD-L1 expression. But overall, the impact appears to be positive, because such heterozygosity appears to potentiate anti–PD-L1-based therapy (Figure 5G).

Next, we evaluated the phenotype of CD8^+^ T cells using the same experimental schedule (Supplemental Figure 18A). We gated live CD45^+^CD3^+^CD8^+^ cells from 4 groups and applied Uniform Manifold Approximation and Projection (UMAP) dimension reduction and FlowSOM clustering to identify clusters with distinct marker expression patterns (Supplemental Figure 18B). Naive-like CD8^+^ T cells (CD44^–^ CD62L^+^) is a type of T cell that has not yet been fully activated, and the proportion of this subset was lower in the *Ppp2r2a^+/–^* group than in the *Ppp2r2a^+/+^* group, with a trend that PD-L1 blockade further decreased their proportion by activating this subset (Supplemental Figure 18B).

Further analysis of multiple suppressive markers revealed that the suppressive phenotype of Tregs was diminished in the *Ppp2r2a^+/–^* group, compared with *Ppp2r2a^+/+^* (Supplemental Figure 18, C–G). This result suggests that the Tregs have an impaired function. To further address this point, we co-cultured Tregs derived from *Ppp2r2a^+/−^* or *Ppp2r2a^+/+^* tumors, with wild-type CD8^+^ T cells to assess Treg suppressive capacity. CD8^+^ T cell proliferation, measured by CellTrace Violet–based flow cytometry, was significantly higher in the *Ppp2r2a^+/−^* group compared with the *Ppp2r2a^+/+^* group (Supplemental Figure 18, H and I). Therefore, Treg activity is suppressed in *Ppp2r2a^+/−^* tumors. Altogether, *Ppp2r2a* heterozygosity enhances the efficacy of PD-L1 blockade tumors by reshaping the TME, particularly through stronger suppression of Tregs induced by *Ppp2r2a* deficiency.

### CD8^+^ T cells, CD4^+^ T cells, and NK cells are required for the Ppp2r2a deficiency–induced increase in PD-L1 antibody efficacy.

**Figure 6:**
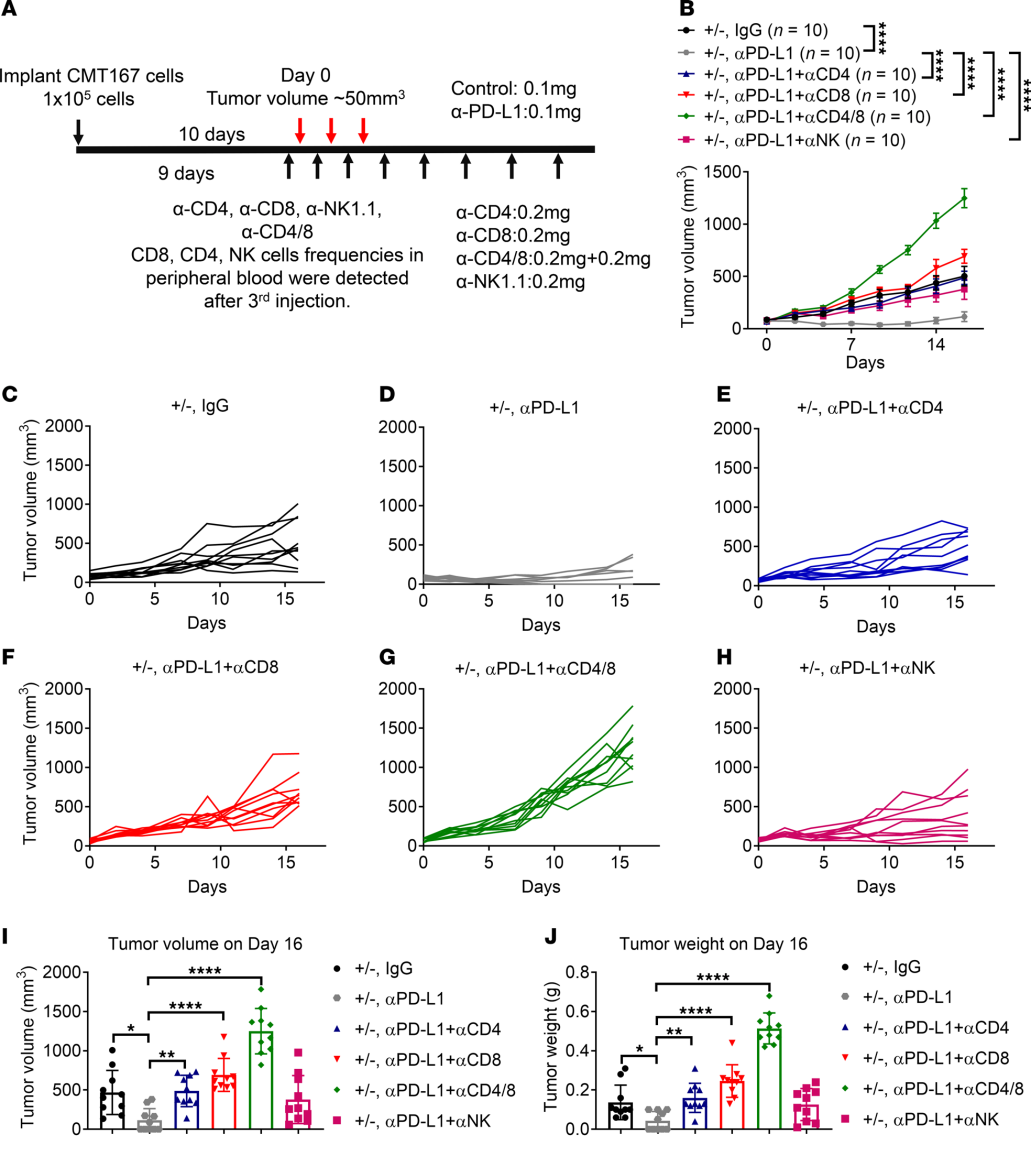
CD8^+^ T cells, CD4^+^ T cells, and NK cells are required for *Ppp2r2a* heterozygosity–mediated synergy with PD-L1 blockade. (**A**) Experimental design for immune cell depletion in CMT167 *Ppp2r2a^+/–^* tumors treated with control or PD-L1 antibodies. (**B–H**) Tumor growth curves showing loss of therapeutic efficacy upon depletion of CD4^+^, CD8^+^, or NK cells. (**I–J**) Quantification of tumor volumes and weights on day 16. Data are presented as mean ± SEM (*n =* 10). *P < 0.05, **P < 0.01, ****P < 0.0001 by 1-way or 2-way ANOVA with Bonferroni post hoc test.

Based on our findings that *Ppp2r2a* heterozygosity in tumors leads to an increased infiltration of NK cells while reducing Treg infiltration and that *Ppp2r2a* heterozygosity combined with PD-L1 blockade results in tumor regression in mice and an increase in the proportion of CD8^+^ T cells, we hypothesized that CD8^+^ T cells, CD4^+^ T cells, and NK cells are necessary for the antitumor efficacy induced by Ppp2r2a deficiency and PD-L1 blockade. To test this hypothesis, *Ppp2r2a^+/–^* tumor-bearing mice were treated with control or PD-L1 antibodies, and NK cells and CD4^+^, CD8^+^, and CD4/8^+^ T cells were depleted using depletion antibodies (Figure 6A). Among all the treatment groups, the PD-L1 antibody-alone group had the slowest tumor growth. However, depletion of these immune cell types abolished the inhibitory effect on tumor growth observed in the PD-L1 blockade monotherapy group (Figure 6B). Tumor growth curves for each group are demonstrated in Figure 6, C–H. The reductions in tumor volume (Figure 6I) and weight (Figure 6J) observed in the PD-L1 blockade group were reversed when CD8^+^ T cells, CD4^+^ T cells, and CD4/8^+^ were depleted. These results demonstrate that CD8^+^ T cells, CD4^+^ T cells, and NK cells are required for the efficacy of PD-L1 blockade in mice bearing *Ppp2r2a^+/–^* tumors. Of note, both helper T cells (CD4^+^ T cells) and Tregs express CD4 on their surface. Thus, CD4 depletion by this antibody approach depletes both types of immune cells.

### Blocking IFN signaling by IFNAR1 neutralization abolishes the antitumor efficacy of a PD-L1 antibody in Ppp2r2a^+/–^ KO CMT167 tumors.

**Figure 7:**
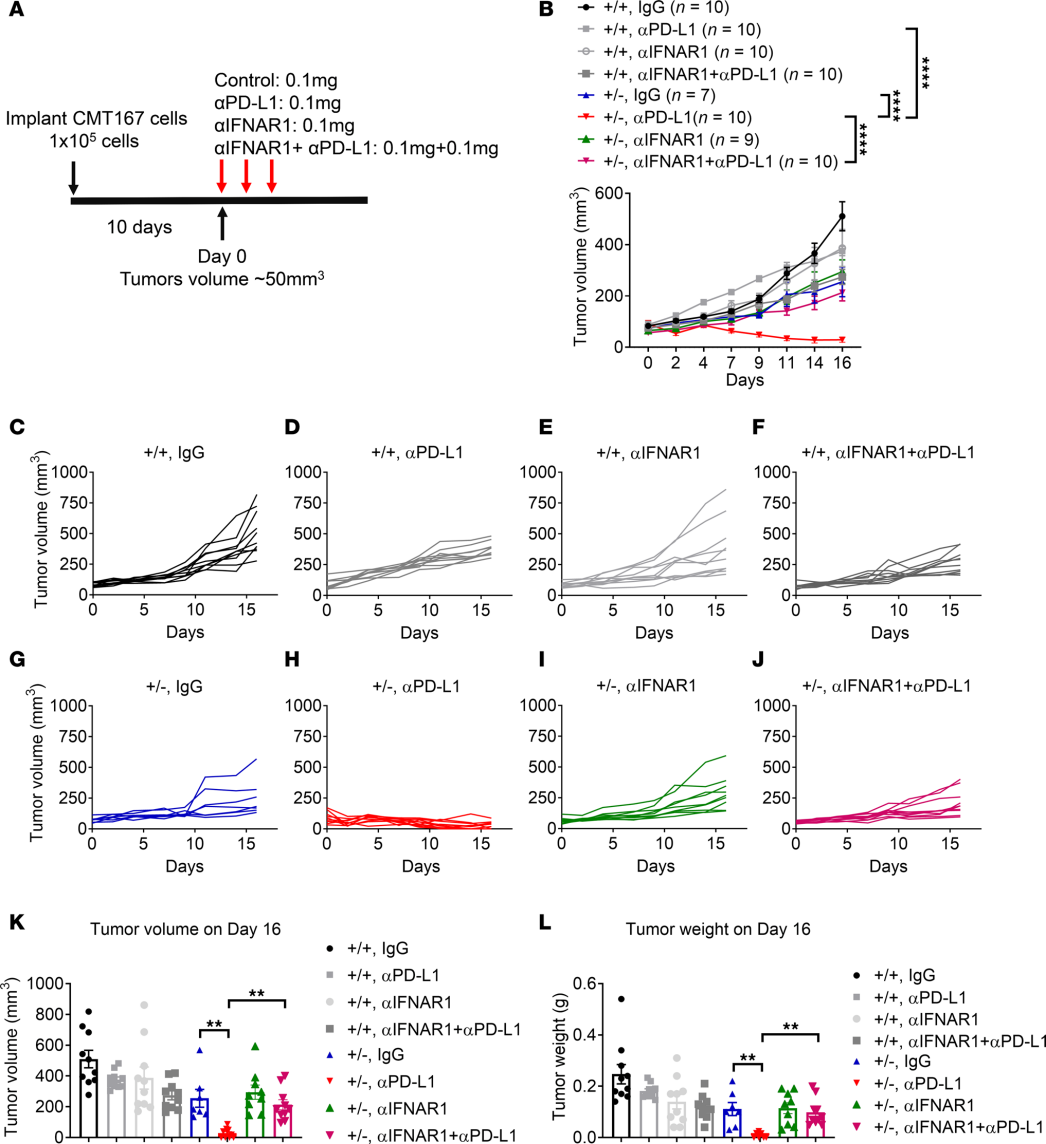
IFNAR1 neutralization abrogates PD-L1 blockade–induced regression in *Ppp2r2a^+/–^* tumors. (**A**) Treatment schedule in mice bearing *Ppp2r2a^+^/^+^* or *Ppp2r2a^+/–^* CMT167 tumors. (**B–J**) Tumor growth curves with control or anti–PD-L1 antibody ± IFNAR1 neutralization. (**K–L**) Quantification of tumor volumes and weights at endpoint. Data are presented as mean ± SEM. (*n =* 10). **P < 0.01, ****P < 0.0001 by 1-way or 2-way ANOVA with Bonferroni post hoc test.

To determine whether type I IFN signaling contributes to Ppp2r2a deficiency–induced sensitivity to PD-L1 antibody treatment, we blocked type I IFN signaling using an anti-IFNAR1 antibody and assessed its impact on PD-L1 antibody efficacy in treating *Ppp2r2a^+/–^* tumors (Figure 7A). The abrogation of type I IFN signaling by the IFNAR antibody reversed the antitumor efficacy of PD-L1 blockade in *Ppp2r2a^+/–^* CMT167 tumors (Figure 7B). The growth curves for individual tumors of each group are presented in Figure 7, C–J. Moreover, the reductions in tumor volume (Figure 7K) and weight (Figure 7L) observed in the PD-L1 blockade group on the cutoff day were also abolished when IFNAR1 was simultaneously blockaded. Thus, in support of the role of type I IFNs in the Ppp2r2a heterozygosity-induced potentiation of PD-L1 efficacy, IFNAR1 neutralization abrogates *Ppp2r2a* heterozygosity-related PD-L1 antibody sensitivity.

To further evaluate the role of type I IFN signaling in Tregs, we adoptively transferred either wild-type or *Ifnar*-deficient Tregs into diphtheria toxin–pretreated Foxp3-DTR mice. Briefly, we modified this Treg replacement protocol in which Foxp3-DTR mice were treated daily with diphtheria toxin to deplete endogenous Tregs and then reconstituted the mice with Tregs from either wild-type or *Ifnar1^–/–^* donors. We then implanted *Ppp2r2a^+/–^* cells into the host, and followed with treatment with the PD-L1 antibody (Supplemental Figure 19A). Compared with the group with wild-type Treg reconstitution, the group with *Ifnar1^–/–^* Treg reconstitution exhibited blunted PD-L1 antibody’s antitumor activity, suggesting that without the IFNAR signal in Tregs, Tregs had more of an inhibitory effect, which promoted the tumor growth (Supplemental Figure 19B, left panel), and, as a feedback loop, there were more Tregs infiltrating into the TME (Supplemental Figure 19B, right panel). This suggests that type I IFN signaling in Tregs contributes to the efficacy of anti–PD-L1 in eliminating Ppp2r2a-deficient tumor cells.

To further illustrate the phenotype of tumor infiltrating Tregs, we performed a high-dimensional flow cytometry analysis on the tumor infiltrating Tregs and displayed the data in 2 dimensions by performing dimension reduction using the UMAP approach (Supplemental Figure 19C). Unsupervised clustering analysis with FlowSOM enabled differential expression analysis between groups, identifying 17 distinct clusters. The percentages of clusters 12 and 13 were significantly enriched in mice reconstituted with *Ifnar1^–/–^* Tregs and bearing *Ppp2r2a^+/–^* tumors (Supplemental Figure 19C, left). These 2 clusters shared the characterization of increased CD39, ICOS, CLTA-4, CD44, and GITR, indicating that this subset of Tregs exhibited more suppressive phenotype (Supplemental Figure 19D).

### The CD8^+^ T cell phenotype of PD-L1 antibody–treated mice bearing Ppp2r2a^+/–^ tumors in the presence and absence of blocking IFNs signaling by IFNAR1 neutralization.

**Figure 8:**
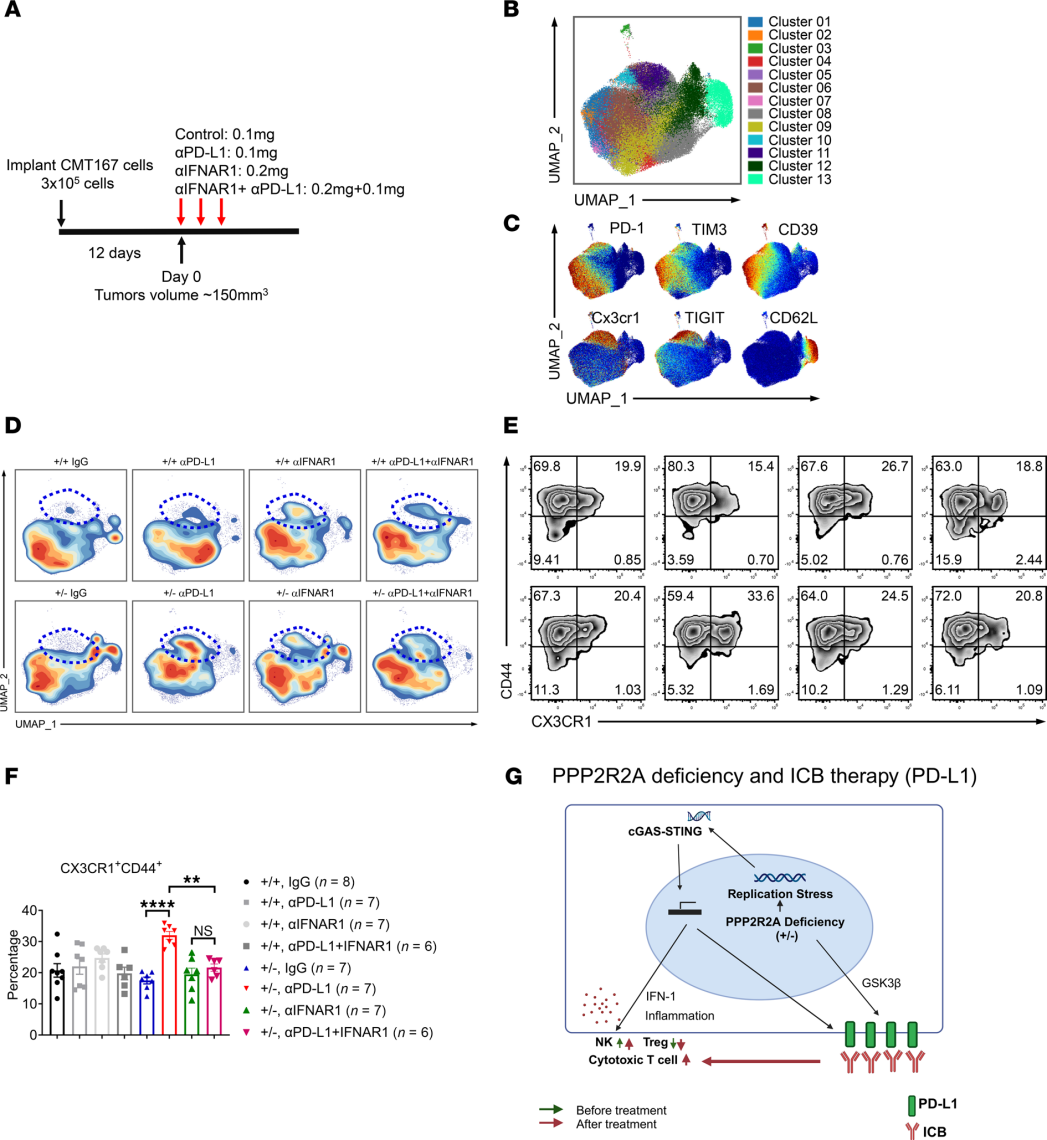
Type I IFN signaling drives cytotoxic CD8^+^ T cell expansion and the antitumor efficacy of PD-L1 blockade in Ppp2r2a*^+/–^* tumors. (**A**) Treatment schedule. (**B**) Visualization of CD8^+^ T cell flow cytometry data obtained from day 7 tumor samples. Tumor samples were collected from indicated groups, and UMAP dimension reduction and FlowSOM clustering were applied to identify clusters with distinctive marker expression patterns. (**C**) Key marker expressions were overlaid onto UMAP space. (**D**) Contour plots were generated to display population dynamics across the groups. (**E**) Representative flow cytometry plot showing CX3CR1 and CD44 expression levels in CD8^+^ T cells. (**F**) Representative quantification of CD44^+^ CX3CR1^+^ CD8^+^ T cells. (**G**) Working model of PPP2R2A deficiency in ICB response. Data are presented as mean ± SEM. **P < 0.01, ****P < 0.0001 by Tukey’s multiple comparison test.

Lastly, we investigated the role of type I IFN signaling in the therapeutic efficacy of PD-L1 blockade in mice bearing *Ppp2r2a^+/–^* tumors, particularly in relation to that of CD8^+^ T cells, because we detected differences in Treg phenotype. To address this, mice were implanted with either *Ppp2r2a^+/–^* or *Ppp2r2a^+/+^* tumors and treated with the anti–PD-L1 antibody (aPD-L1), an anti-IFNAR1 antibody (aIFNAR1), or a combination of both antibodies (aPD-L1 plus aIFNAR1) (Figure 8A). We gated live CD8^+^ T cells, followed by UMAP dimension reduction and FlowSOM clustering, to define distinct subpopulations (Figure 8B). The heatmap of the clusters displayed in Figure 8B is shown in Supplemental Figure 20. Key marker expression patterns were overlaid onto the UMAP space to characterize each cluster (Figure 8C). Contour plots were generated to visualize population dynamics, revealing a notable increase in CD44^+^Cx3cr1^+^CD8^+^ T cells exclusively in the aPD-L1–treated *Ppp2r2a^+/–^* tumor-bearing mice, highlighted by the blue dotted line in Figure 8D. This subset exhibited intermediate PD-1 expression along with no or low expression of other inhibitory markers, suggesting that they are effector/activated cells (29–32). The increase of cytotoxic CD8^+^ T cells (CD44^+^Cx3cr1^+^CD8^+^ T) after aPD-L1 antibody treatment suggests that a highly developed and strongly activated subset of cytotoxic T cells was increased only in PD-L1 antibody–treated *Ppp2r2a^+/–^* tumors. In support of this observation, we measured IFN-γ and TNF-α expression in re-stimulated single-cell suspensions from *Ppp2r2a^+/+^* and *Ppp2r2a^+/–^* tumor samples by intracellular staining (Supplemental Figure 21A). CD8^+^ T cells from *Ppp2r2a^+/–^* tumors produced higher levels of both cytokines. Additionally, IL-2^–^ perforin^+^ T cell numbers were also dramatically increased in the PD-L1 antibody–treated *Ppp2r2a^+/–^* tumors (Supplemental Figure 21, B–D). These results indicate enhanced polyfunctionality of CD8^+^ T cells, especially in the PD-L1 antibody–treated group. Notably, this expansion of cytotoxic CD8^+^ T cells in aPD-L1–treated mice with *Ppp2r2a^+/–^* tumors was abolished upon IFNAR1 blockade, underscoring the critical role of type I IFN signaling in the formation of this subset (Figure 8, E and F).

In summary, our findings support a model where PPP2R2A deficiency in NSCLC enhances the efficacy of PD-L1 ICB through activation of the cGAS-STING-IFNs pathway. Tumor PPP2R2A deficiency reprograms the TME by increasing NK cell infiltration, reducing Treg infiltration, and upregulating PD-L1 expression. These changes in the TME make PPP2R2A-deficient tumors more responsive to PD-L1 blockade therapy (Figure 8G).

## Discussion

Tumor cells frequently harbor unique genetic alterations that influence therapeutic responses. In this study, we report that heterogeneous alterations in *PPP2R2A* reshape the TME by increasing the proportion of NK cells, reducing Tregs, and elevating PD-L1 expression. These changes occur via the activation of the cGAS-STING-IFN pathway. *PPP2R2A*-deficient tumors were more responsive to PD-L1 blockade, indicating that *PPP2R2A*-deficient NSCLC may be effectively targeted with PD-L1–associated ICB. Thus, *PPP2R2A* deficiency holds the potential to be a predictive biomarker for the efficacy of ICB in treating NSCLC.

dsDNA in the cytoplasm of eukaryotic cells serves as a primary trigger for cGAS-STING activation. PPP2R2A is implicated in homologous recombination repair (8). Moreover, PPP2R2A deficiency leads to oncogene-induced RS and DNA damage (10, 11). Consistent with the concept that RS and DNA damage produces cytosolic DNA—a critical driver of cGAS-STING activation—PPP2R2A deficiency increases cytosolic DNA and MN formation, which are associated with activation of the cGAS-STING-IFN pathway.

We acknowledge that 1 limitation of our study is that the precise mechanisms by which RS activates cGAS in PPP2R2A-deficient cells remain unclear. However, previous studies have suggested that RS can promote cGAS activation through cytosolic DNA (20, 33, 34). When replication forks stall under stress, they become fragile and susceptible to degradation. This process can release single DNA and dsDNA fragments into the cytoplasm, activating cGAS. In addition, cytosolic DNA can appear as MN, which form after cell division due to RS (35). When the MN envelope ruptures, cGAS can recognize the escaped DNA and trigger an immune response (22, 36, 37). Thus, MN may represent a source of cytosolic DNA that activates the cGAS-STING pathway in PPP2R2A-deficient cells. However, whether MN is directly linked to cGAS activation remains debated (38, 39). Because MN are only 1 form of cytosolic DNA, RS can also cause nuclear DNA release in other forms that activate cGAS-STING-IFN signaling (40). Therefore, RS-induced cytosolic DNA, whether as MN or other forms, has the potential to trigger immune responses.

Although systemic pharmacologic inhibition of PP2A by inhibitors increases sensitivity to ICB (41, 42), and KD of PPP2R2D (a B regulatory subunit of PP2A) in T cells enhances the cytotoxic function T cells in melanoma models (43), the effects of intrinsic PPP2R2A deficiency on ICB remain unexplored. The cGAS-STING-IFN pathway is important for ICB efficacy. Consistent with the increased cGAS-STING-IFN pathway in PPP2R2A-deficient cells, we found that heterozygosity of *PPP2R2A* in tumors is associated with increased sensitivity to PD-L1 blockade. This activation influences immune cell recruitment and their activity, including NK and Tregs, even before PD-L1 antibody treatment. However, the cGAS-STING-IFN activation may extend beyond tumor cells; cytosolic DNA from tumor cells can be taken up by neighboring immune cells, thereby triggering cGAS-STING activation in immune cells (44). Consequently, the cGAS-STING and IFN signaling in immune cells may also enhance the efficacy of PD-L1 blockade in PPP2R2A-deficient tumors.

Supporting the role of type I IFNs in the antitumor activity of PD-L1 antibodies in tumors with PPP2R2A deficiency, the blockade of type I IFNs signaling via blocking IFNAR negated the increased sensitivity to PD-L1 antibodies observed in mice bearing *Ppp2r2a^+/–^* tumors. However, our study also highlights that although cytosolic DNA is essential for cGAS-STING activation, type I IFN expression may not always be evident. For example, despite RS, cytosolic DNA accumulation, and cGAS-STING activation in LLC cells with PPP2R2A deficiency, type I IFN expression was not upregulated for reasons that remain unclear. This finding underscores the importance of assessing the integrity of the entire cGAS-STING-IFN axis rather than relying solely on cGAS-STING activation as a biomarker.

Although systemic pharmacological PP2A inhibition may not fully replicate the effects of PPP2R2A inactivation, preclinical models show significant synergy between PP2A inhibitors and PD-1 therapy (41, 42, 45). For example, LB-100, a direct PP2A inhibitor, enhances ICB sensitivity in mouse colon and melanoma models by activating mTORC1 signaling, which reduces the differentiation of naive CD4 cells into Tregs. Additionally, PP2A inhibition also increases neoantigen expression by altering mRNA splicing (46) and promotes microsatellite instability through epigenetic silencing (47). Tumors with high genomic instability often exhibit elevated neoantigen expression. Our study suggests that *PPP2R2A* heterozygosity induces RS, DNA damage, and genomic instability in tumor cells, which may explain the increased TMB observed in PPP2R2A-deficient NSCLC based on analyses of TCGA cohorts. Therefore, it is plausible that PPP2R2A deficiency also influences neoantigen expression and perhaps TMB, with multiple potential impacts on ICB outcomes.

It is important to note that NSCLC is a heterogeneous disease often driven by specific oncogenic mutations, which distinguish its subtypes. In clinical practice, analyzing multiple tumor mutations is recommended owing to the therapeutic implications of drugs targeting EGFR, KRAS, ALK, ROS1, BRAF, NTRK1/2/3, MET, or RET (48). Tumors with these driver mutations exhibit distinct clinical characteristics compared with other tumors, and immunotherapy is typically not the first-line treatment (49). Therefore, it would be intriguing to investigate the impact of PPP2R2A deficiency in specific NSCLC subtypes, both with and without oncogene driver mutations. Further studies are needed to evaluate the outcomes of combining PD-L1 blockade with standard therapies, such as chemotherapy, radiation therapy, or targeted therapies, in treating PPP2R2A-deficient NSCLC. Such investigations could provide valuable insights into optimizing therapeutic strategies for this subset of patients with lung cancer.

Type I IFNs respond to viral infection and regulate antitumor activity by modulating innate immunity, adaptive responses, and Tregs (50, 51). In PPP2R2A-deficient tumors, the most notable TME change is reduced Treg abundance and activity. Type I IFNs can affect Tregs directly or indirectly; our data show that intrinsic *Ifnar1* KO in Tregs reverses PD-L1 antibody–mediated antitumor activity in *Ppp2r2a^+/–^* tumors, indicating a Treg-intrinsic type I IFN pathway, though indirect effects cannot be excluded. Although PPP2R2A deficiency has not been directly linked to immune antitumor activity, PP2A is essential for Treg function, and its loss disrupts metabolism and cytokine profiles, preventing suppression of effector responses (52). Pharmacologic PP2A inhibition (LB-100) combined with anti–PD-1 or CAR-T ICB induces durable antitumor responses in preclinical colon cancer, melanoma, and glioblastoma models (42, 53, 54). Moreover, inactivation of Ppp2r1a, the PP2A scaffolding subunit, promotes microsatellite instability, neoantigen production, and tumor immunogenicity in colorectal, breast, pancreatic cancers and ovarian clear cell carcinoma (47, 55). Thus, immune effects may differ among PP2A subunits, and the role of PPP2R2A deficiency in neoantigen production and ICB therapy warrants further study.

Although the roles of type I IFNs in regulating Tregs remain unclear and sometimes controversial, evidence links their antitumor activity to Treg modulation (51). IFN-α therapy has shown antitumor and immunomodulatory effects in cancers such as melanoma, partly by increasing tumor-infiltrating cells and reducing the number of circulating Tregs (56). Tregs lacking IFNAR are less sensitive to type I IFNs (57). In human Tregs, IFN-α disrupts TCR signaling and suppresses cAMP induction, impairing Treg function (58). Recent studies also suggest that the functional stability and activity of Foxp3^+^ Tregs are tightly regulated by cytokines (59). Other cytokines also shape Treg activity; for example, IL-6 downregulates Foxp3 and induces IL-17, whereas cytosolic dsDNA in tumor cells promotes CCL22-mediated Treg recruitment, despite reports that IFN-α inhibits CCL22 (60). These opposing effects likely reflect Treg-extrinsic interferon activity (51). Furthermore, PD-L1/PD-1 signaling negatively regulates Tregs during infection (61, 62). Thus, PPP2R2A deficiency may suppress Treg activity through type I IFN– and PD-L1–mediated mechanisms.

In addition to Tregs, type I IFNs activate NK cells during antitumor responses, both directly and indirectly through other immune cells (50, 63). Investigating the effect of IFNAR-deficient NK cells on Ppp2r2a deficiency–induced ICB efficacy enhancement would be an interesting direction for future studies. Additionally, type I IFNs act in a complex manner. Type I IFN activity can either support or impair host defense; this dual effect is strongly influenced by when and how much IFN is produced, as well as the cellular context in which signaling occurs (64). Identifying specific cytokines involved in promoting NK cell infiltration and suppressing Treg infiltration and activity will be a critical area of research.

PD-L1 is the first approved biomarker for anti–PD-1 therapy, but its predictive value is limited, because only approximately 45% of patients with PD-L1-high NSCLC respond, and some develop hyperprogression (65, 66). We found PPP2R2A deficiency increases PD-L1 via GSK3β and cGAS-STING, with the PPP2R2A/PD-L1 ratio correlating strongly with ICB outcomes, suggesting improved predictive accuracy. PPP2R2A deficiency also induces DNA damage and genomic instability; TCGA data confirm higher TMB in PPP2R2A-deleted NSCLC, though this is not clearly linked to poor prognosis. Because combining biomarkers may enhance prediction (67), PPP2R2A deficiency—through effects on PD-L1 and perhaps also TMB—represents a promising candidate. Supporting evidence includes PPP2R1A mutations associated with exceptional ICB response in ovarian cancer (68) and PP2A inhibition sensitizing tumors to PD-1/PD-L1, CTLA4, and CAR-T therapies (41).Thus, PPP2R2A deficiency warrants investigation as a predictive biomarker for ICB.

### Conclusion.

This study shows that cGAS-STING-IFN activation occurs in PPP2R2A-deficient cells without exogenous DNA damage. We reveal a link between PPP2R2A loss and PD-L1 expression and demonstrate its impact on Treg and NK cells in the TME. PPP2R2A deficiency both promotes and restrains antitumor immunity, and PD-L1–targeted ICB amplifies its positive effects, enhancing therapeutic efficacy. These results suggest PD-L1 ICB as a promising strategy for PPP2R2A-deficient NSCLC and support PPP2R2A deficiency as a predictive biomarker for anti–PD-L1 therapy.

## Methods

### Sex as a biological variable.

Our study examined male and female animals, and similar findings are reported for both sexes.

### Cell lines.

H1299, HEK-293T, A549, CMT167, and LLC cells were cultured in DMEM medium (Hyclone); H1437 cells were cultured in 1640 medium (Hyclone) supplemented with 10% FBS (Gibco) in a humidified atmosphere containing 5% CO_2_ at 37°C. Cells that had been passaged 10 or fewer times were used for experiments. CMT167 and LLC cells were gifts from Terrence Williams (City of Hope National Medical Center, Duarte, CA). All other cells were obtained from ATCC in 2016. Authentication was performed via short tandem repeat profiling by the MCIC Genomics Core at Ohio State University in 2020. *Mycoplasma* contamination was ruled out in all cell lines, using the LookOut Mycoplasma PCR Detection Kit (MP0035, Sigma) in 2020.

### Generation of KO cells.

*Ppp2r2a* KO cells were generated by using CRISPR/Cas9 technology. The CRISPR design site (https://crispor.gi.ucsc.edu/) was used to identify guide RNA target sites flanking ENSMUSE00000267666 (exon 3) of the *Ppp2r2a* gene (NM_028032.3).

The following guide RNAs were used for mouse *Ppp2r2a* KO: –5′ GAACATCCTTAGTGAGTTGAGGG 3′ for the 5′ end of exon 3, and – 5′ CCTCAGTAATAAGTTGACCTCTC 3′ for the 3′ end of exon 3. Oligonucleotides were phosphorylated and annealed and then cloned into *BsmBI* (ER0451, Fermentas) digested plasmid lentiCRISPR v2 (plasmid 52961, Addgene). The lentivirus production and transduction were conducted according to the protocol from Addgene. Forward (5′ TCCCAGGCTACAAGAGACCAC 3′) and reverse (5′ GCACAACACAGACATTTAAGGC 3′) primers flanking the *Ppp2r2a* exon 3 and amplifying a product of 961 bp from the parental cells were used to characterize *Ppp2r2a* gene editing results in the single clones.

The following plasmids were used to generate the *STING* KO human and mouse cells: pLentiCRISPRv2-STING_gRNA3 (plasmid 127640, Addgene) for human cells and pLentiCRISPRv2_mSTING_gRNA_1 (plasmid 196625, Addgene) for mouse cells. The generation of *PPP2R2A* KO cells was as previously described (12).

### Comet assay.

Neutral comet assays were performed using the Comet Assay Kit (Trevigen, 4250-050-K) according to the manufacturer’s protocol. Comet tail analysis was conducted using TriTek CometScore software (version 2.0.0.38).

### Plasmids.

All shRNAs were obtained from Sigma Aldrich (Supplemental Table 2). pBabe GSK3β and pbabe GSK3β S9A were as previously described (12).

### RNA extraction and real-time quantitative reverse transcription PCR.

Total RNA was isolated using the RNeasy Mini Kit (74016, Qiagen) and subsequently treated with RNase-free DNase to eliminate genomic DNA contamination. cDNA was synthesized from 1 μg of total RNA using the Transcriptor First Strand cDNA Synthesis Kit (4897030001, Roche) with oligo(dT) primers. Real-time quantitative reverse transcription PCR (qRT-PCR) was performed as previously described (10–12). Primer sequences used for qRT-PCR are provided in Supplemental Table 3.

### Immunoblotting.

Immunoblotting was conducted as previously described (10, 11). The primary antibodies used for Western blotting are listed in the Supplemental Table 4. Band intensities from Western blot images were quantified using ImageJ software (National Institutes of Health). Bands were selected using the Rectangle tool, and regions of interest were defined for each band. Pixel intensity was measured using the gel analysis function. Protein density values were normalized to the loading control, β-actin. The dilution for each antibody is shown in Supplemental Table 4.

### Immunofluorescence and cytosolic DNA staining.

For cytosolic DNA staining, cells seeded on cover slips were incubated with Pico488 (NC1878927, Lumiprobe) for 1 hour. After staining, the cover slips were mounted and sealed using Antifade Mounting Medium with DAPI (H-1500-10, Vector Laboratories). Representative images were taken from a Zeiss LSM510 META confocal microscope.

### Cytosolic DNA extraction and quantification.

Cell fractionation was performed using NE-PER Nuclear and Cytoplasmic Extraction Reagents (78833, ThermoFisher). A SpectraMax Quant AccuClear Nano dsDNA Assay Kit (R8357, Molecular Devices) and a BioTek Synergy H1 microplate reader were used to determine the concentration of cytosolic DNA.

### Tumor models.

Both male and female C57/BL6 mice (strain 000664, The Jackson Laboratory), 6–8 weeks of age, were used for this study. The mice were bred at The Ohio State University. Xenografts were established by subcutaneous injections of CMT167 cells or LLC cells (1 × 10^5^ cells) with Matrigel (356235, Corning) into the flank of the mice. Tumor diameters were measured with digital calipers, and volumes of the tumors were calculated using the following formula: volume = (width)^2^ × length/2. Once tumor volume reached 50 mm^3^, the mice were randomized into 1 of the following treatment groups: CMT167 *Ppp2r2a^+/+^* cells with control (rat IgG2b in vivo isotype control, 100 μg/dose; ICH2243, clone 1-2, Ichorbio); *Ppp2r2a^+/+^* cells with α-PD-L1 (anti-mouse PD-L1 in vivo antibody, 100 μg/dose; ICH1086, clone 10F.9G2, Ichorbio); *Ppp2r2a^+/–^* with control; or *Ppp2r2a^+/–^* with α-PD-L1. Treatments were given every 2 days every 2 days for 3 doses. All mice were maintained under barrier conditions, and the experiments were conducted using protocols and conditions approved by the IACUC of The Ohio State University.

### Tissue digestion, cell isolation, and flow cytometry.

To isolate tumor, tissues were dissected and incubated for 20 minutes at 37°C with collagenase D (1 mg/mL; 45-11088882001, Roche). Digested tissue was then filtered through a 40 μm nylon strainer (MSPP-15-1040, VWR). Blood cells were removed with RBC lysis buffer (420301, BioLegend). Cell suspension was washed with PBS. For flow cytometry staining, cells were washed twice in FACS buffer and FcR blocking was applied for 10 minutes at 4°C. Live/dead staining was performed for 10 minutes at 4°C with LIVE/DEAD Blue before staining with the surface antibody mix (described in Supplemental Table 5) for 30 minutes at 4°C in FACS buffer. For intracellular staining, a Foxp3/Transcription Factor Staining Buffer Set (00-5523-00, eBioscience) was used according to the manufacturer’s protocol. Cells were then incubated with antibodies for 1–3 hours in permeabilization buffer. Samples were analyzed immediately on a Cytek Aurora system, and data analysis was performed using FlowJo (Tree Star) or OMIQ software (https://www.omiq.ai/).

### Immune cell depletion assay.

At age 7 weeks, C57/BL6 mice were implanted with CMT167 *PPP2R2A^+/–^* cells as described in Tumor Models. After 9 days, mice were randomized to 1 of the following treatment groups: control (rat IgG2b in vivo isotype control, 100 μg/dose; ICH2243, clone 1-2, Ichorbio); α-PD-L1 alone (anti-mouse PD-L1 in vivo antibody, 100 μg/dose; ICH1086, clone 10F.9G2, Ichorbio), α-PD-L1 + α-CD4 (anti mouse CD4, 200 μg/dose, BP0003, Clone:GK1.5, BioXcell); α-PD-L1 plus α-CD8 (anti-mouse CD8, 200 μg/dose; BP0004, clone 53-6.7, BioXcell); α-PD-L1 plus α-CD4/8 (anti-CD4 plus anti-CD8 antibodies, 200 μg/dose for each antibody); and α-PD-L1 plus α-NK (anti-mouse NK1.1, 200 μg/dose; BP0036, clone PK136, BioXcell). Depletion antibodies were given on day 9 and day 11 after inoculation, then twice weekly until endpoints were reached. Control and PD-L1 antibodies were given on days 10, 12, and 14 after the inoculation of tumors.

### IFNAR1 neutralization assay.

CMT167 *Ppp2r2a^+/+^* and *Ppp2r2a^+/–^* cells were implanted in 7-week-old C57/BL6 mice as described in the previous paragraph. After 9 days, mice were randomized to control alone (rat IgG2b in vivo isotype control, 100 μg/dose; ICH2243, clone 1-2, Ichorbio); α-PD-L1 alone (anti-mouse PD-L1 in vivo antibody, 100 μg/dose; ICH1086, clone 10F.9G2, Ichorbio); control plus α-IFNAR1 (anti-mouse IFNAR1, 200 μg/dose; BP0241, clone MAR1-5A3, BioXcell); and α-PD-L1 plus α-IFNAR1. Antibodies were given on days 10, 12, and 14 after the inoculation of tumors.

### Statistics.

Statistical significance was assessed using Prism 10.3 (GraphPad Software). Tumor growth curves were analyzed with repeated-measures 2-way ANOVA. Survival analysis was conducted using the log-rank (Mantel-Cox) test. A 2-tailed Student’s *t* test was used for comparisons between 2 groups. For multiple-group comparisons, 1-way ANOVA followed by Bonferroni post hoc analysis was applied when variance was not significantly different across groups. A *P* value of less than 0.05 was considered statistically significant.

### Data availability.

The bulk RNA-Seq data have been deposited in the NCBI Gene Expression Omnibus under accession number GSE311238. All values underlying the data presented in the graphs and as means are available in the Supporting Data Values file.

### Study approval.

All mouse studies were conducted under protocols approved by the IACUC of The Ohio State University (no. 2018A00000049).

## Author contributions

JRZ and ZL designed the study. JRZ wrote the manuscript with critical input from all the coauthors. ZQ, NJS, AL, CBP, and XZ conducted experiments and acquired data. ZQ, NJS, AL, DPC, CBP, CY, QEW, XZ, ZL, and JRZ analyzed data. All authors reviewed and approved the manuscript.

## Funding support

This work is the result of NIH funding, in whole or in part, and is subject to the NIH Public Access Policy. Through acceptance of this federal funding, the NIH has been given a right to make the work publicly available in PubMed Central.

- National Cancer Institute grants R01 CA R01 CA240374, R01 CA249198, and R21 CA226317 to JRZ.
- The American Lung Association Lung Cancer Discovery Award to JRZ.
- Department of Defense Lung Cancer Research Program, W81XWH2010868 (supporting JRZ).
- The Ohio State University James Comprehensive Cancer Intramural Research Program Pelotonia Idea Award to JRZ.
- The Ohio State University Comprehensive Cancer Center (OSUCCC).
- NIH grants R01CA282501 and R01CA262069 (to ZL) and P30CA016058.
- This research was made possible through resources, expertise, and support provided by the Pelotonia Institute for Immuno-Oncology, which is funded by the Pelotonia community and the OSUCCC.

## Supplementary Material

Supplemental data

Unedited blot and gel images

Supplemental table 1

Supplemental table 2

Supplemental table 3

Supplemental table 4

Supplemental table 5

Supplemental table 6

Supporting data values

## Version 1 12/18/2025

In-Press Preview

## Version 2 02/16/2026

Electronic publication
