# Supplementary material for: PPP2R2A insufficiency enhances PD-L1 immune checkpoint blockade efficacy in lung cancer through cGAS-STING activation
Source: J Clin Invest. 2025 Dec 18;136(4):e193354. doi: 10.1172/JCI193354 (PMC12904717; doi:10.1172/JCI193354)
Supplement: Supplemental data [file jci-136-193354-s040.pdf]

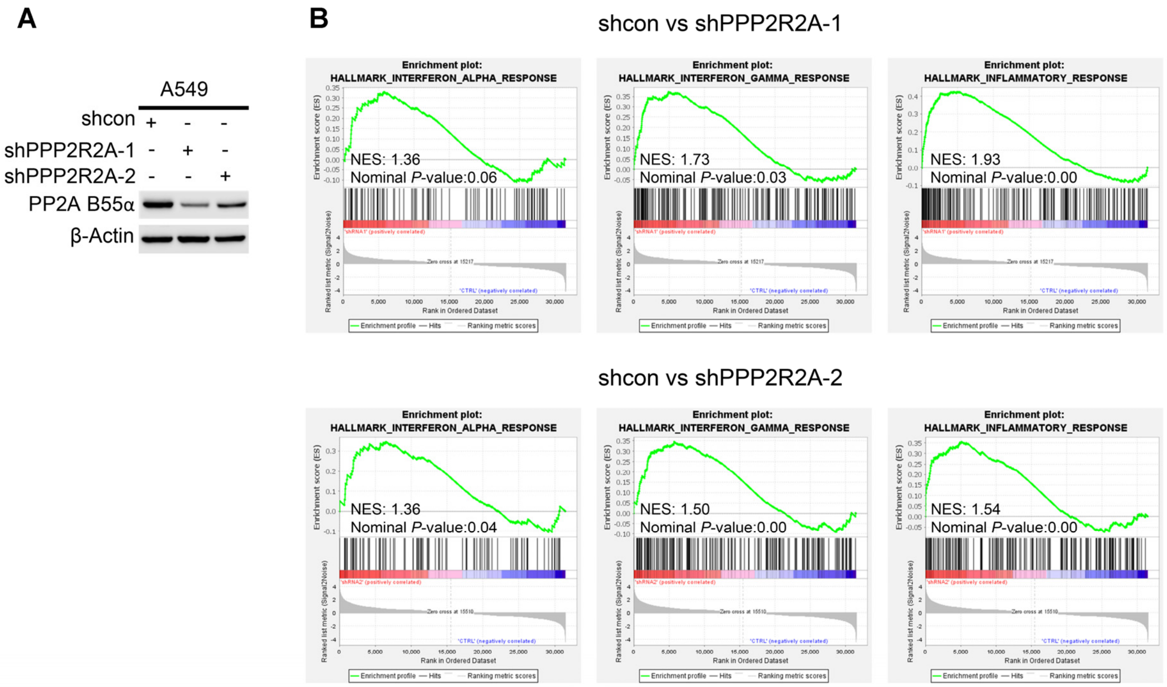

Supplementary Figure 1. *PPP2R2A* knockdown in A549 cells leads to the inflammatory response. (**A**) Western blot of PPP2R2A in A549 cells. (**B**) Bulk RNA-seq was conducted in the cells with or without *PPP2R2A* knockdown. Interferon-alpha response, interferon-gamma response and inflammatory response pathways are enriched based on the gene expression data from the comparison between control and two knockdown conditions. Gene Set Enrichment Analysis (GSEA) and hallmark gene sets from the Molecular Signatures Database (MSigDB) were utilized to identify enriched pathways in **B**.

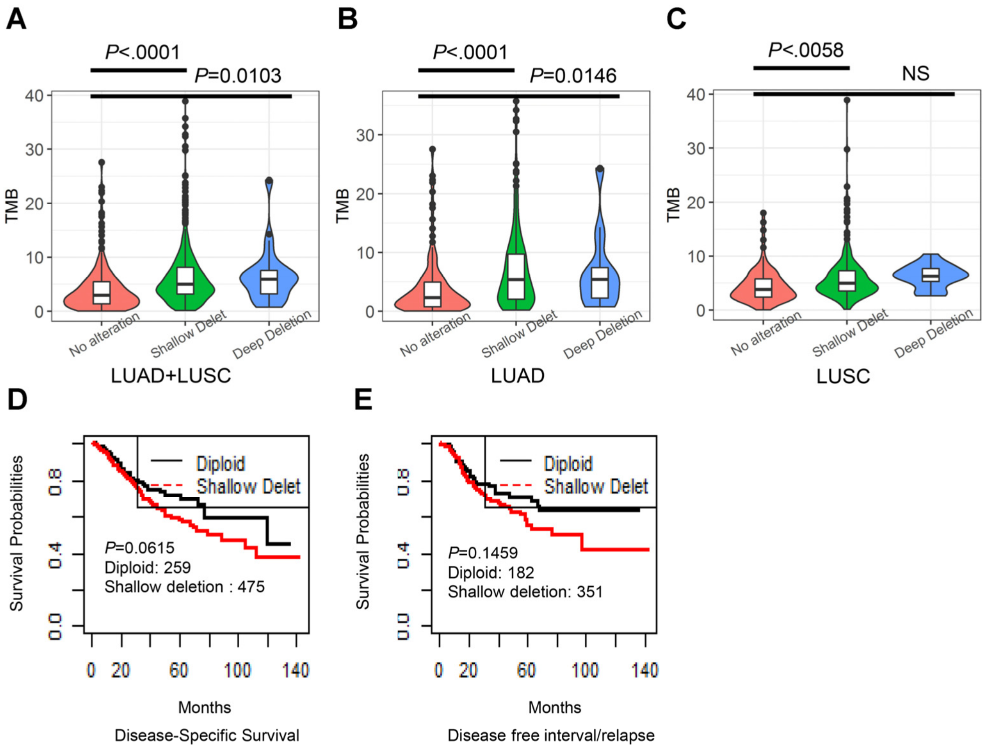

Supplementary Figure 2. *PPP2R2A* deletion is associated with reduced PPP2R2A expression. (**A-C**) Tumor mutational burden (TMB) in NSCLC samples stratified by PPP2R2A status. TMB was calculated using the TCGA somatic mutation data of lung cancer obtained from the GDC somatic MAF file. Lung adenocarcinomas (LUAD) and squamous cell carcinoma (LUSC), the most common two subtypes of NSCLC, were included. (*n*=965 for total NSCLC with both gene expression and copy-number alteration data, *n*=40 with deep deletion, *n*=532 with shallow deletion; *n*=111 with gain, *n*=282 with no alteration; ANOVA was used for analysis. (**D, E**) Kaplan–Meier survival curves for NSCLC patients stratified by PPP2R2A status. TCGA pan-cancer RNA-Seq data with patient survival information was downloaded from Xena browser: https://xenabrowser.net/datapages/) and then combined with PPP2R2A copy-number variation data downloaded from Cbioportal).

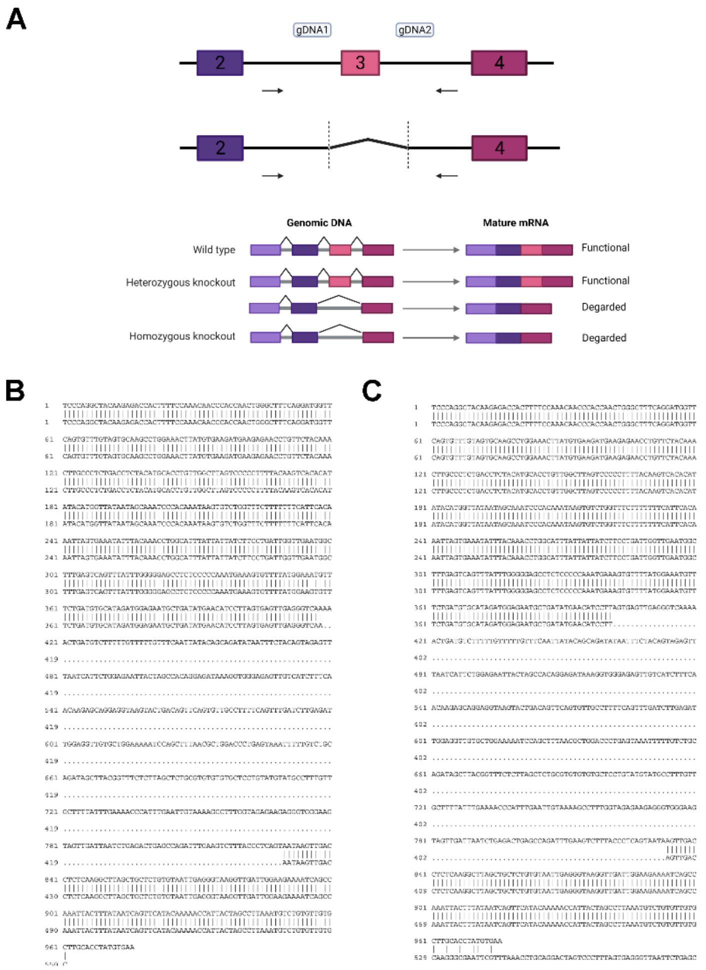

Supplementary Figure 3. CRISPR/Cas9 targeting strategy to knockout *PPP2R2A* in mouse and human cells. (**A**) Location of sgRNAs used to target mouse and human PPP2R2A exon 3. Schematic of the genomic DNA and mRNA of *PPP2R2A* wild-type (+/+), *PPP2R2A* heterozygous (+/-) allele and *PPP2R2A* homozygous (-/-) allele. The deletion of exon 3 of *Ppp2r2a* are shown in two sequence alignment of the *Ppp2r2a* wide-type and *Ppp2r2a* heterozygous in CMT167 cells (**B**) and LLC cells (**C**).

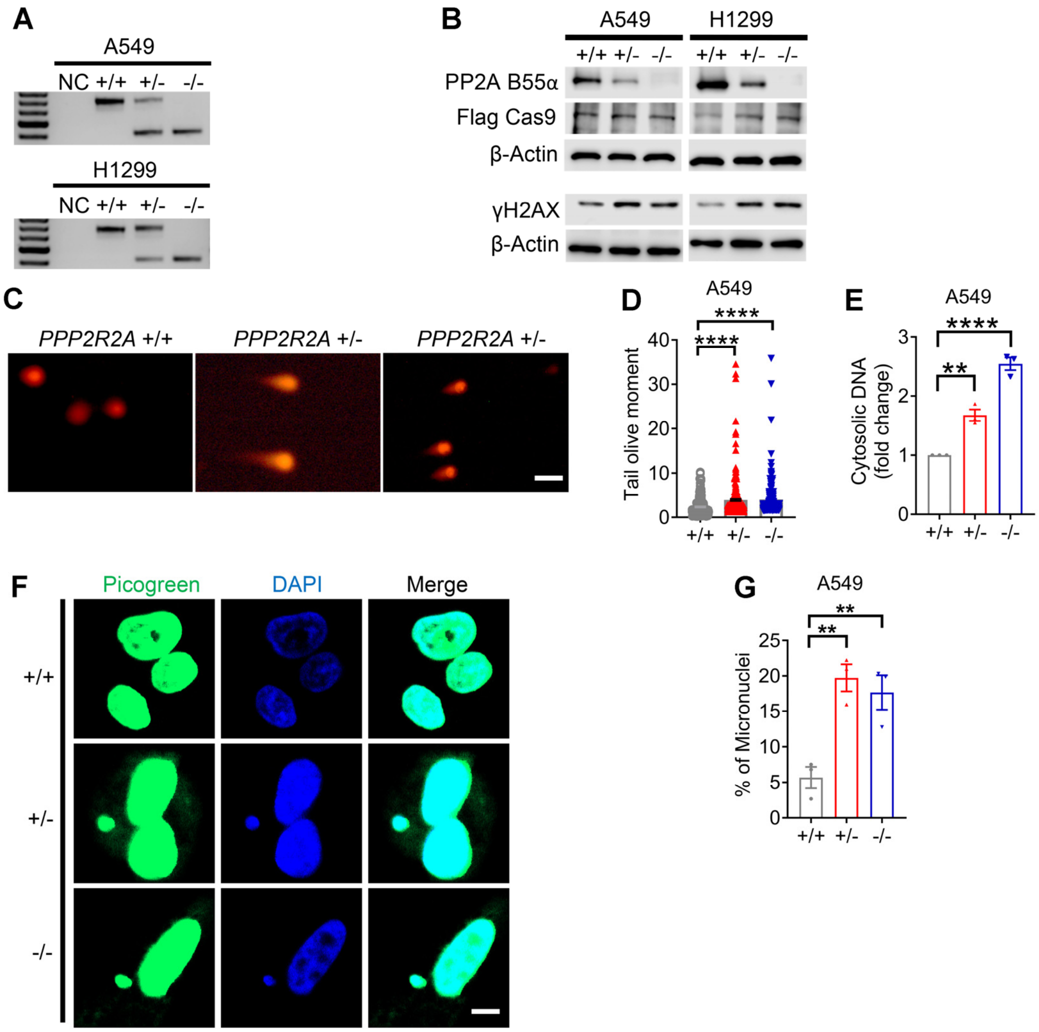

Supplementary Figure 4. *PPP2R2A* knockout leads to the accumulation of cytosolic DNA in A549 cells. (**A**) Genotyping of *PPP2R2A* in A549 and H1299 cells with *PPP2R2A* knockout. (**B**) Western blot analysis of PP2A B55α in human lung cancer cells. (**C-D**) Neutral comet assay results in *PPP2R2A*$^{+/-}$ and *PPP2R2A*$^{-/-}$ A549 cells. Representative images are displayed in **C**, statistical analysis results are presented in **D**. Scale bar is 200 μm in **C**. Data are represented as mean ± SEM from three biological repeats. (**E-G**) Assessment of cytosolic DNA accumulation in A549 cells with reduced PPP2R2A expression. Panel (**E**) quantifies cytosolic DNA levels in *PPP2R2A*$^{+/-}$ and *PPP2R2A*$^{-/-}$ cells; panel (**F**) displays representative images of micronuclei formation; and panel (**G**) presents the quantification of cells with micronuclei. Scale bar = 30 μm in **F**. Data are demonstrated as mean ± SEM (n= 3) (**E, G**); **, $P < 0.01$, ****, $P < 0.0001$, statistical significance in **D**, **E** and **G** were determined by one-way ANOVA, followed by Bonferroni post hoc analysis for multiple comparisons.

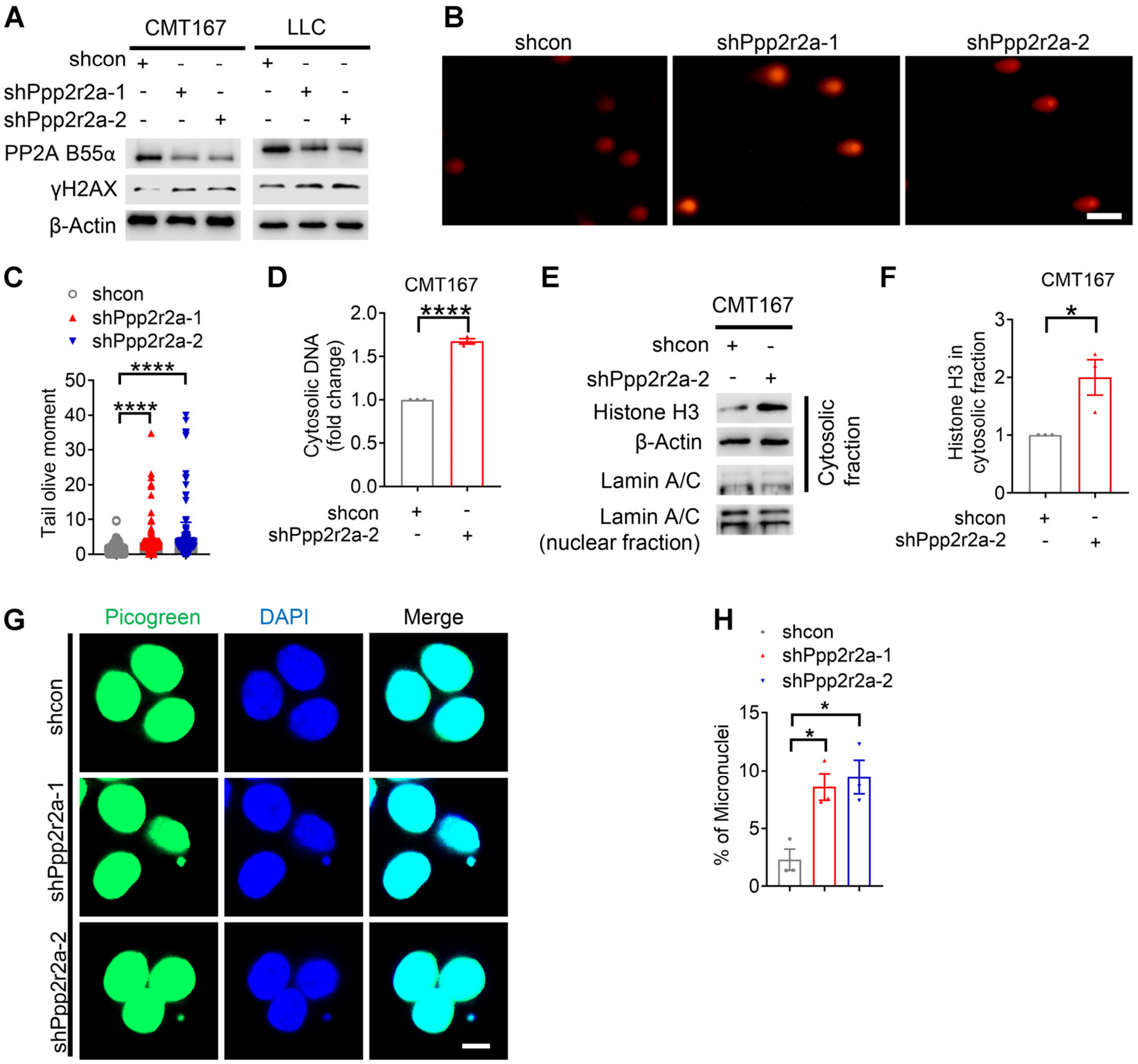

Supplementary Figure 5. Cytosolic DNA is increased due to replication stress in *Ppp2r2a* knockdown CMT167 cells. (**A**) Western blot analysis displaying PP2A B55α protein levels in CMT167 and LLC cells after *Ppp2r2a* knockdown. (**B, C**) Neutral comet assays in *Ppp2r2a* knockdown CMT167 cells. Panel (**B**) shows representative comet assay images (scale bar = 200 µm), and panel (**C**) presents quantification of comet tail moments based on the analysis of 300 cells across three biological replicates. (**D-H**) Assessment of cytosolic DNA accumulation in *Ppp2r2a* knockdown CMT167 cells. Panel (**D**) quantifies cytosolic DNA levels; panels (**E**, **F**) display Western blot analysis of histone H3 in the cytosolic fraction; panel (**G**) shows representative immunofluorescence images of micronuclei; and panel (**H**) provides quantification of the percentage of cells exhibiting micronuclei in **G**. Scale bar = 30 µm in **G.** Data are presented as mean ± SEM (*n*=3) (**D, F, H**); *, $P < 0.05$, ****, $P < 0.0001$, statistical significance in **C**, **D**, **F**, **H** were determined by one-way ANOVA, followed by Bonferroni post hoc analysis for multiple comparisons.

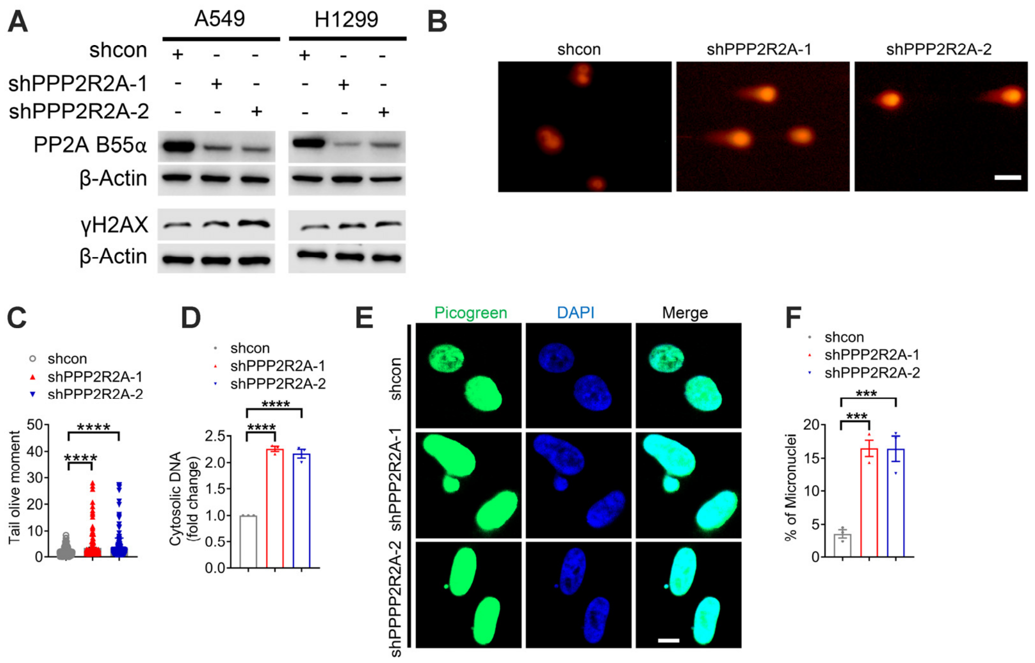

Supplementary Figure 6. *PPP2R2A* knockdown results in the accumulation of cytosolic DNA in A549 cells. (**A**) Immunoblots of PP2A B55α in human lung cancer cells. (**B, C**) Neutral comet assays in PPP2R2A knockdown A549 cells. Representative images are shown in **B**, statistical analysis results are displayed in **C**. Scale bar = 200 μm in **B.** Data are represented as mean ± SEM from three biological repeats. (**D-F**) Evaluation of cytosolic DNA accumulation in A549 cells with reduced PPP2R2A expression. Panel (**D**) provides quantification of cytosolic DNA levels; panel (**E**) shows representative images of micronuclei formation; and panel (**F**) presents the corresponding quantification of the percentage of cells with micronuclei. Scale bar = 30 μm in **E.** Data are shown as mean ± SEM (n=3) (**D, F**); ***, $P < 0.001$, ****, $P < 0.0001$, statistical significance in **C**, **D** and **F** were determined by one-way ANOVA, followed by Bonferroni post hoc analysis for multiple comparisons.

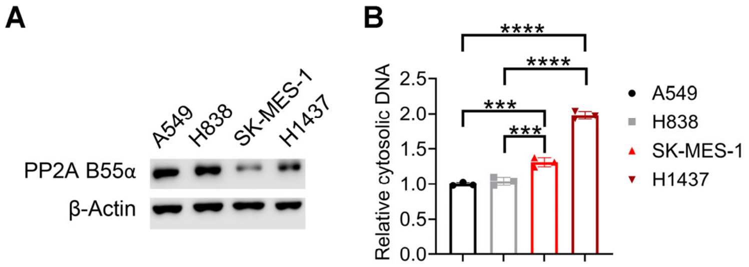

Supplementary Figure 7. SK-MES-1 and H1437 cells with low expression of PPP2R2A exhibited higher levels of cytosolic DNA. (**A**) Immunoblot of PPP2R2A in four NSCLC cell lines. (**B**) Cytosolic DNA quantification in the same cell lines. Data are presented as mean ± SEM (*n* = 3). Statistical analysis was conducted using one-way ANOVA followed by Bonferroni post hoc test for multiple comparisons ***, $P < 0.001$, ****, $P < 0.0001$.

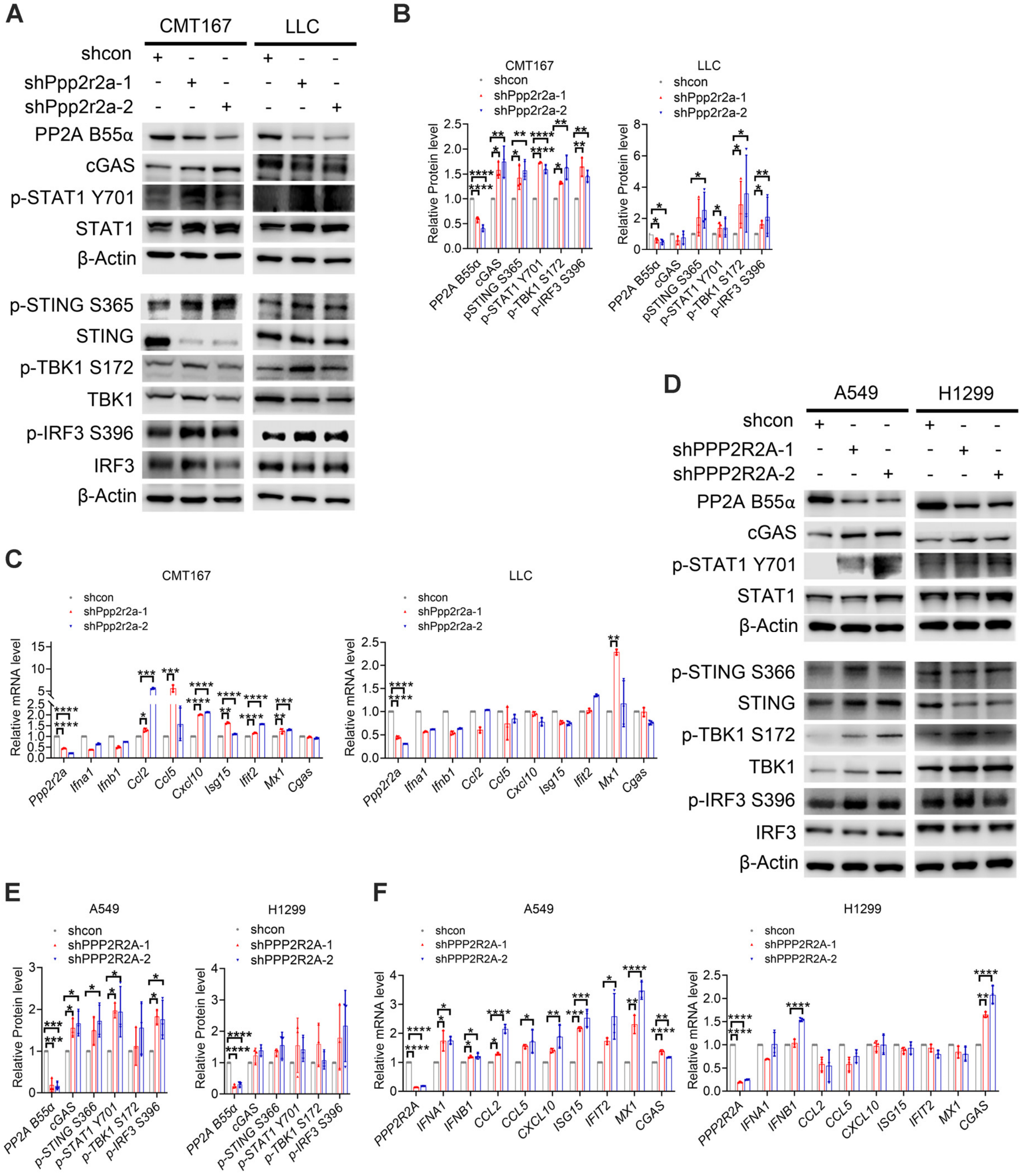

Supplementary Figure 8. Knockdown of *PPP2R2A* activates cGAS-STING and type I IFN pathways in CMT167 and A549 cells. (**A**) Immunoblots analysis of cGAS-STING pathway markers in CMT167 and LLC cells. Quantitative analysis of band intensities from **A** is presented in **B**. (**C**) qPCR measurement of cGAS-STING and IFN pathway genes in CMT167 and LLC cells. (**D**) Western blot of cGAS-STING pathway proteins in A549 and H1299 cells. The quantification results of **D** are shown in **E**. (**F**) cGAS-STING and IFN related genes are

measured in two human lung cancer cells via qPCR. Data are shown as mean ± SEM (n=3) in **B, C, E, F**; Statistical significance was determined using one-way ANOVA followed by Bonferroni post hoc analysis for multiple comparisons: *, $P < 0.05$, **, $P < 0.01$, ***, $P < 0.001$, ****, $P < 0.0001$.

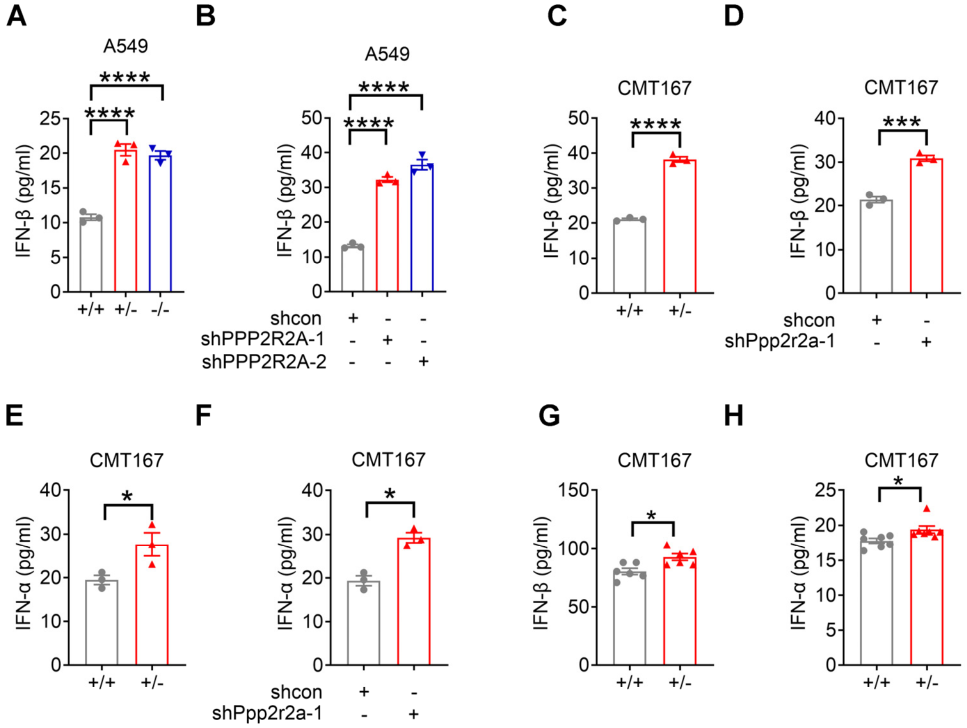

Supplementary Figure 9. *PPP2R2A* loss enhances IFN-β and/or IFN-α secretion in human and mouse cells both *in vitro* and *in vivo.* (**A**, **B**) ELISA analysis of IFN-β secretion in A549 cells. (**C**, **D**) Quantification of IFN-β secretion in CMT167 cells. (**E, F**) ELISA analysis of IFN-α secretion in CMT167 cells. (**G**, **H**) Quantification of IFN-β (**G**) and IFN-α (**H**) levels in serum of CMT167 tumor bearing mice *in vivo*. Data are presented as mean ± SEM (n = 3) in **A**-**F**. Data are presented as mean ± SEM (n = 7) in **G**, **H**. Statistical analysis in **A**, **B** was conducted using one-way ANOVA followed by Bonferroni post hoc test for multiple comparisons. Statistical significance in **C**-**H** was determined by Student's t-test. *, $P < 0.05$, ***, $P < 0.001$, ****, $P < 0.0001$.

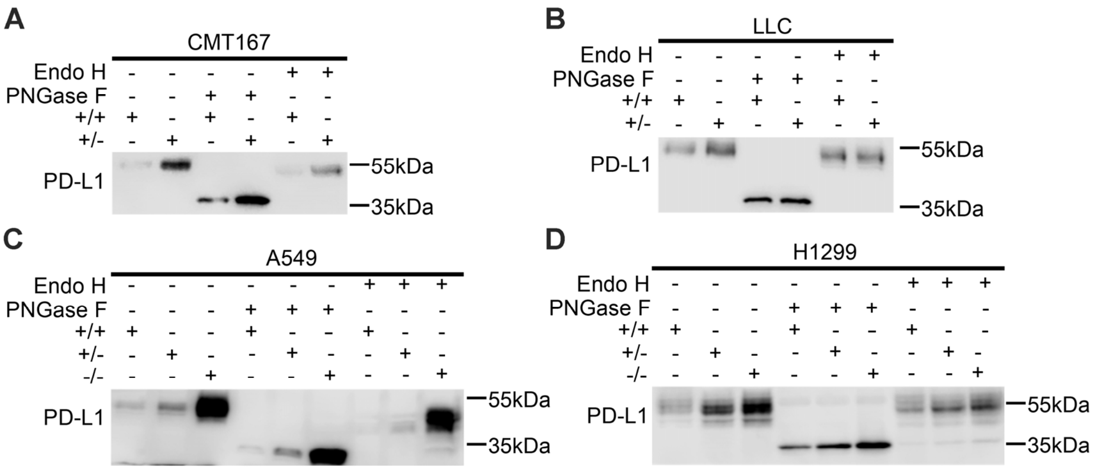

Supplementary Figure 10. Increased expression of PD-L1 triggered by *PPP2R2A* knockout is glycosylated in CMT167 (A), LLC (B), A549 (C) and H1299 (D) cells.

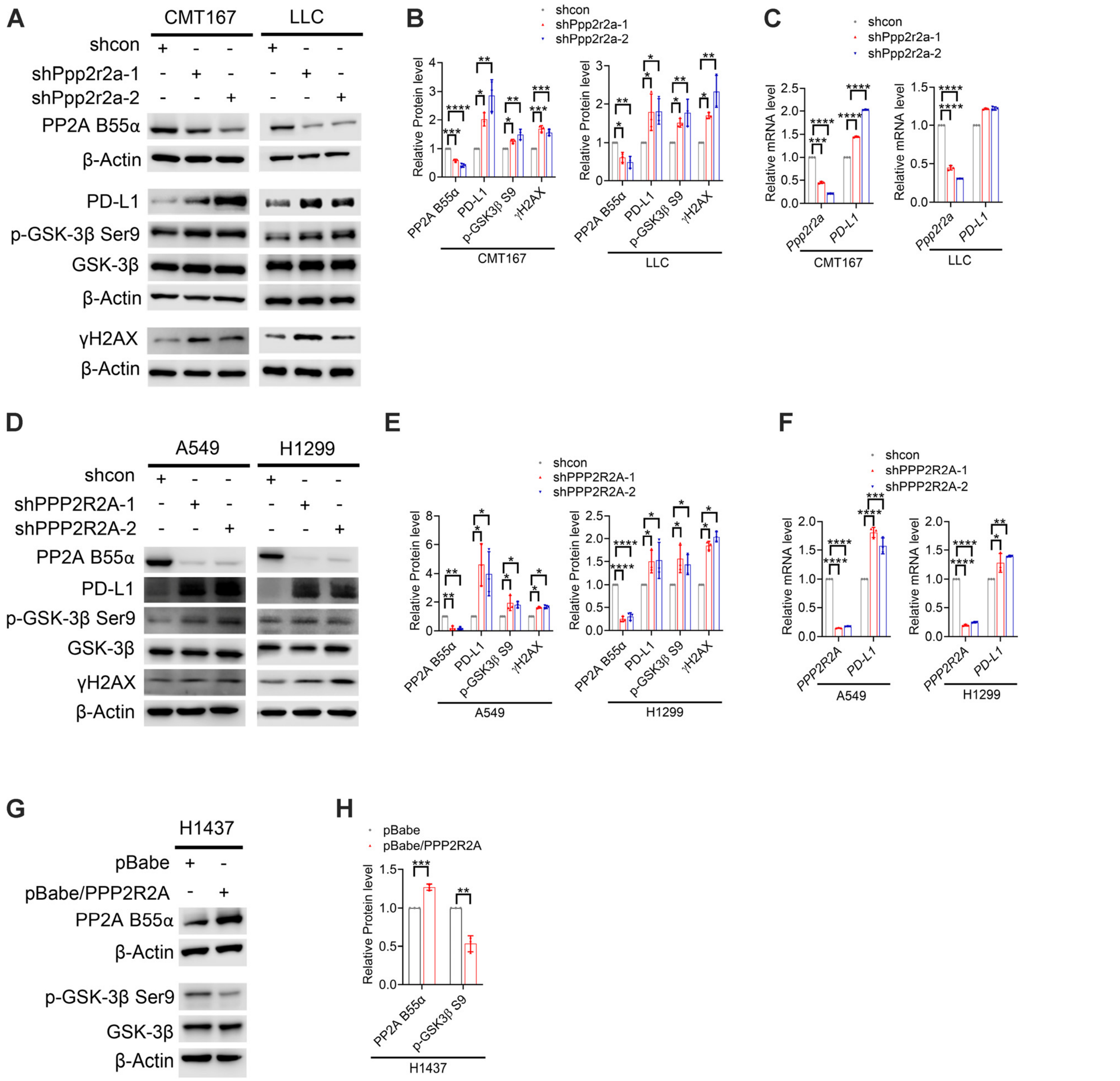

Supplementary Figure 11. PD-L1 expression is upregulated in *PPP2R2A* knockdown lung cancer cells. (**A**, **B**) Western blot analysis of PD-L1 protein levels in CMT167 and LLC cells following *Ppp2r2a* knockdown. Representative Western blot images are presented in **A**, and quantification results are shown in **B**. (**C**) Quantitative PCR analysis of PD-L1 mRNA levels in CMT167 cells with *Ppp2r2a* knockdown. (**D, E**) Western blot analysis of PD-L1 protein levels in A549 and H1299 cells after *PPP2R2A* knockdown. Western blot images in A549 and H1299 cells are shown in **D**, with quantitative data presented in **E**. (**F**) qPCR analysis showing PD-L1 mRNA levels in A549 cells following *PPP2R2A* knockdown. (**G**, **H**) Western blot analysis of GSK-3β phosphorylation in H1437 cells stably overexpressing PPP2R2A. Representative images are shown in **E**, with statistical analysis in **F**. Data in **B**, **C**, **E**, **F** and **H** are presented as mean ± SEM (n=3). Statistical analysis was conducted using one-way ANOVA followed by

Bonferroni post hoc test for multiple comparisons; *, $P < 0.05$, **, $P < 0.01$, ***, $P < 0.001$, ****, $P < 0.0001$.

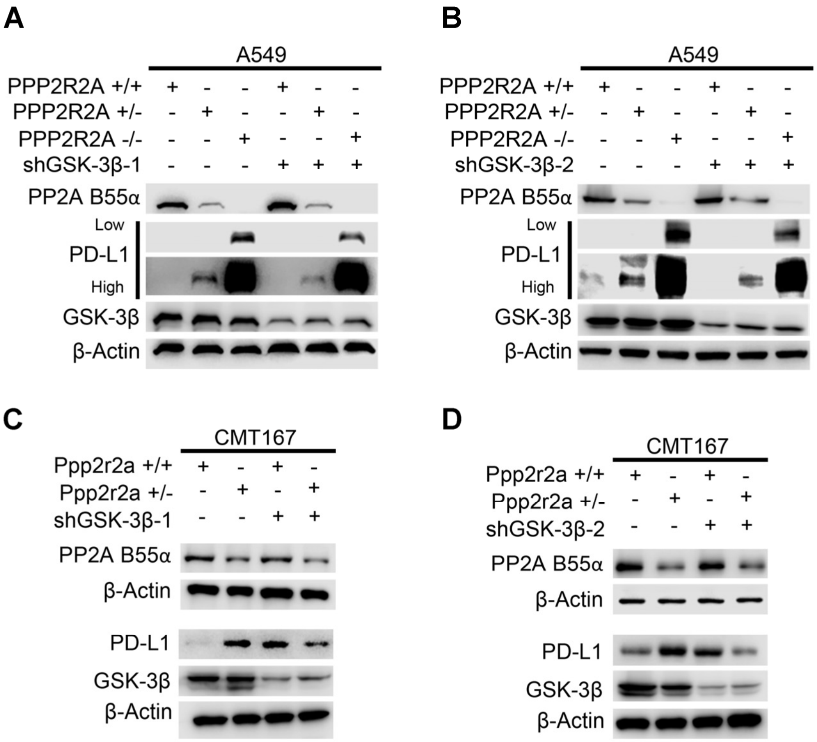

Supplementary Figure 12. Knockdown of GSK-3β partially reduces PD-L1 expression induced by *PPP2R2A* heterozygous **deletion.** (**A**, **B**) Immunoblot analysis of PD-L1 protein levels in *PPP2R2A* knockout A549 cells following GSK-3β knockdown. (**C**, **D**) Western blot of PD-L1 in *Ppp2r2a* heterozygous knockout CMT167 cells with or without GSK-3β knockdown.

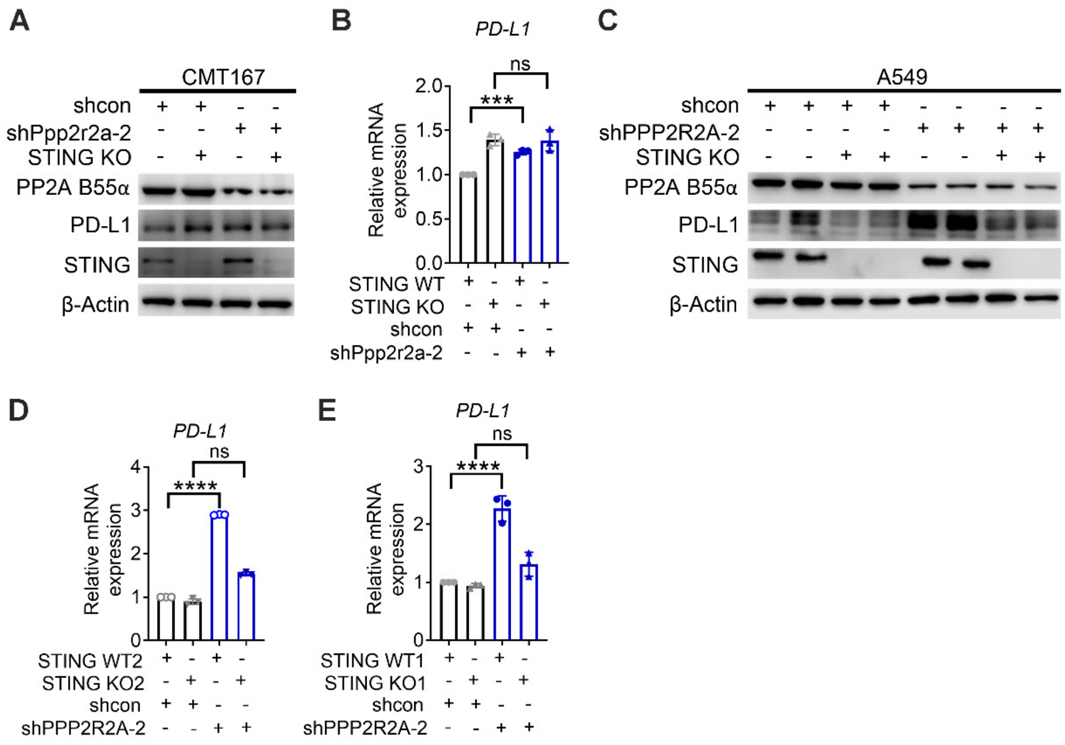

Supplementary Figure 13. STING is important for PPP2R2A deficiency - induced PD-L1 expression in CMT167 and A549 cells. (**A**) Immunoblot analysis of PD-L1 protein levels in *Sting* wild-type or knockout CMT167 cells after *Ppp2r2a* knockdown. (**B**) qPCR analysis of PD-L1 mRNA levels in CMT167 cells after *Ppp2r2a* knockdown, comparing *Sting* wild-type and knockout conditions. (**C**) Immunoblot analysis of PD-L1 protein levels in A549 cells with *PPP2R2A* knockdown, comparing *STING* wild-type and knockout cells. (**D**, **E**) qPCR analysis of PD-L1 mRNA levels in STING wild-type or knockout A549 cells following PPP2R2A knockdown. Data in **B**, **D**, **E** are presented as mean ± SD (n=3). Statistical analysis was conducted using one-way ANOVA followed by Bonferroni post hoc test for multiple comparisons; ns, non-significant; ***, $P < 0.001$, ****, $P < 0.0001$.

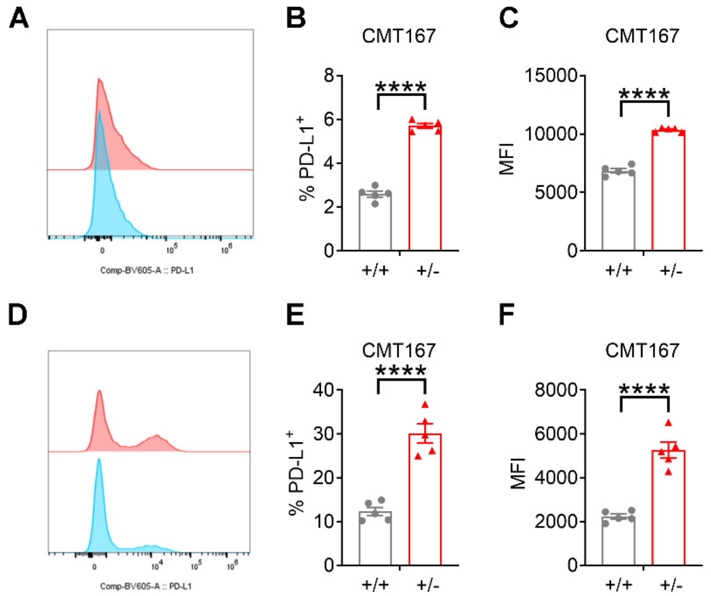

Supplementary Figure 14. Cell surface PD-L1 expression is increased in *Ppp2r2a*$^{+/-}$ cells both *in vitro* and *in vivo*. (**A**) Flow cytometric analysis of PD-L1 expression in CMT167 cells during *in vitro* cell culture. (**B**, **C**) Quantification of PD-L1-positive cell percentage (**B**) and mean fluorescence intensity (MFI) (**C**) in CMT167 cells. (**D**) Flow cytometric analysis of PD-L1 expression in CMT167 *in vivo* tumor samples. (**E**, **F**) Quantification of PD-L1-positive cell percentage (**E**) and MFI (**F**) in CMT167 tumors. Data are presented as mean ± SEM (n = 5 for **B**, **C**, **E**, and **F**). Statistical significance was determined by Student's t-test; ****, $P < 0.0001$.

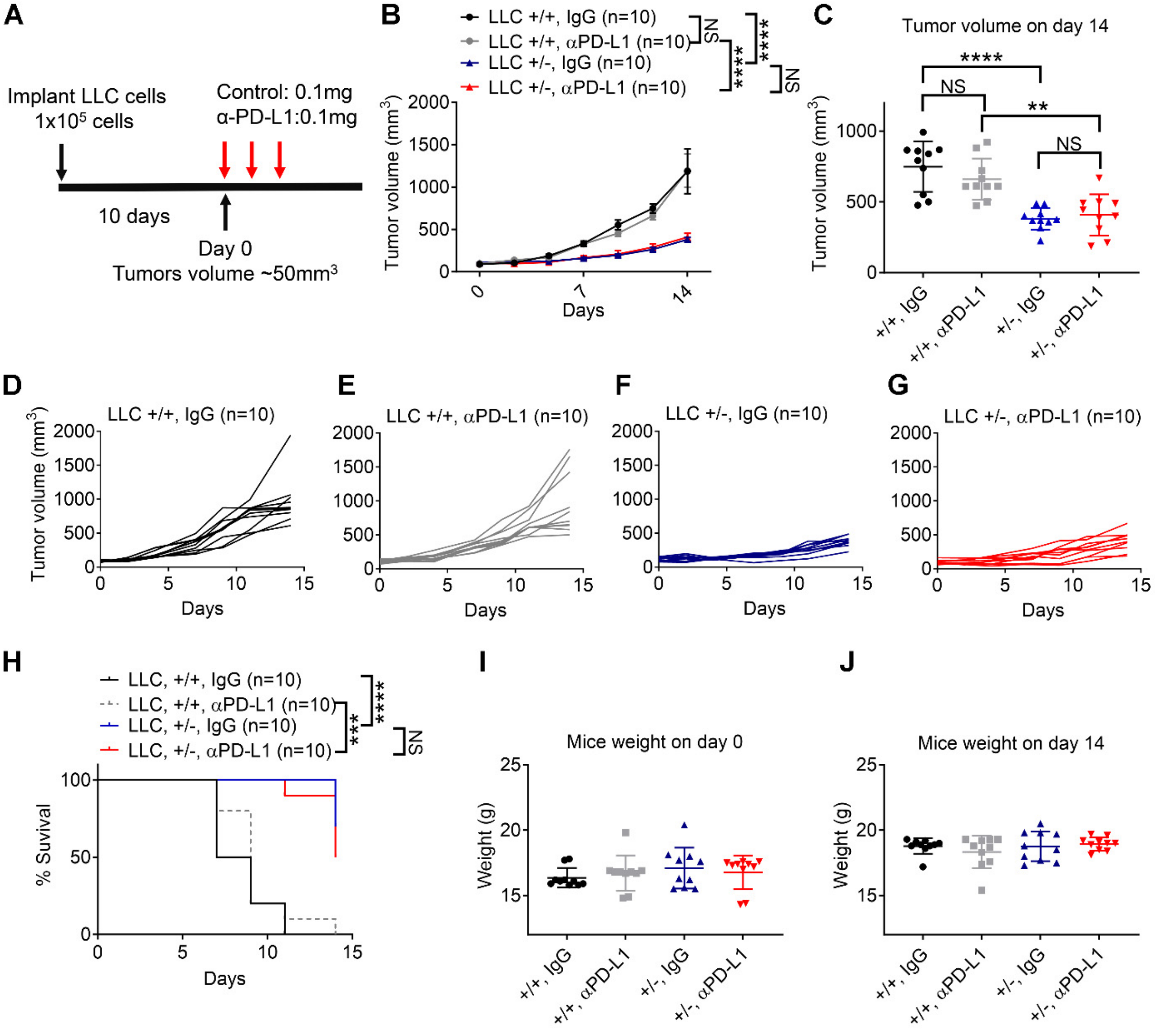

Supplementary Figure 15. Ppp2r2a deficiency does not sensitize LLC cells to PD-L1 antibody treatment *in vivo*. (**A**) Schematic showing schedule of combination of PPP2R2A heterozygous deletion and anti-PD-L1. C57BL/6J mice were implanted with $1x10^5$ LLC cells subcutaneously. Ten days after inoculation, mice were then randomized to two cohorts and treated with control or anti-PD-L1 antibodies for three doses. (**B**) Tumor volume measurements over time in *Ppp2r2a*$^{+/+}$ or *Ppp2r2a*$^{+/-}$ LLC tumors treated with PD-L1 blockade compared to control treatment. NS, not significant; ****, $P < 0.0001$, two-way ANOVA, followed by Bonferroni post hoc analysis for multiple comparisons was used to determine statistical significance. Quantification of tumors volume obtained on day 14 after first dose (**C**). **, $P < 0.01$, ****, $P < 0.0001$, Statistical significance was determined by one-way ANOVA, followed by Bonferroni post hoc analysis for multiple comparisons. Individual tumor growth curves for each group were shown in **D-G**. (**H**) Kaplan–Meier survival curves comparing the survival of mice bearing *Ppp2r2a*$^{+/+}$ or *Ppp2r2a*$^{+/-}$ LLC tumors treated with PD-L1 antibody versus control antibody. ***, $P < 0.001$, ****, $P < 0.0001$, Kaplan–Meier analysis was used to determine significance (**H**). Weight measurements for each group on day 0 (**I**) and day 14 (**J**).

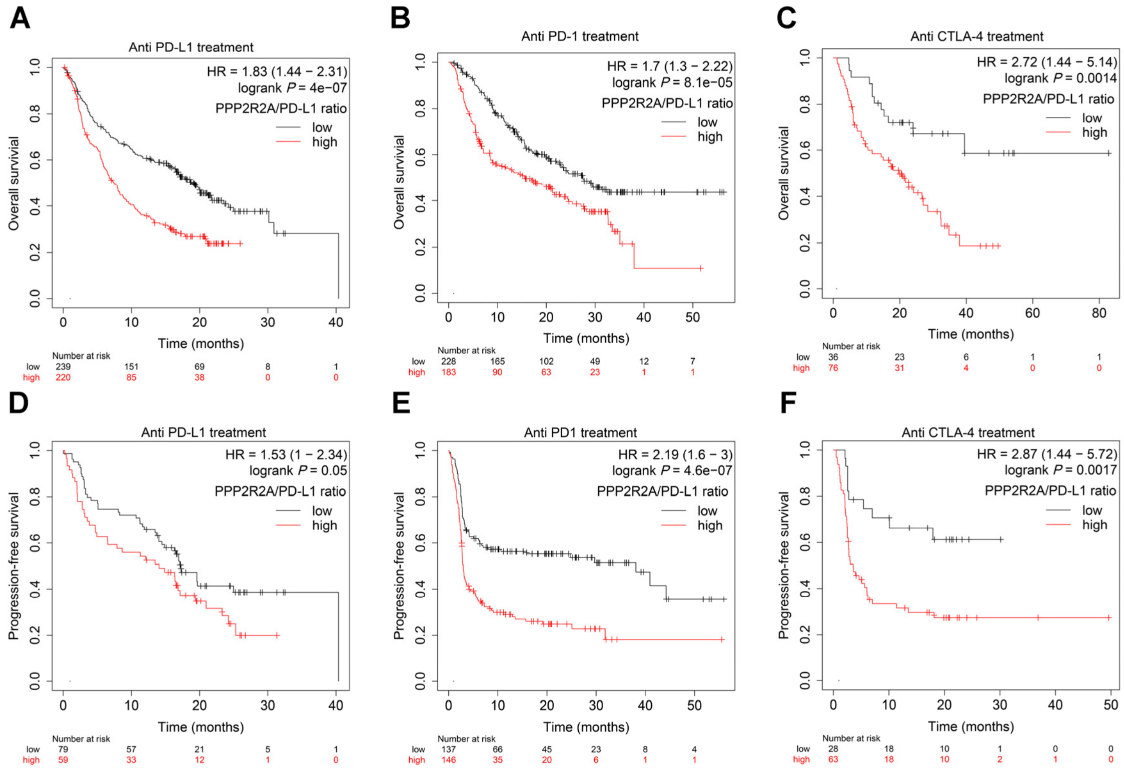

Supplementary Figure 16. Patients with cancer with a low PPP2R2A/PD-L1 ratio have a better response to immune checkpoint inhibitors. (**A**-**C**) Kaplan–Meier survival curves illustrating overall survival in cancer patients stratified by PPP2R2A/PD-L1 expression ratio: (**A**) Individuals with a low PPP2R2A/PD-L1 ratio receiving PD-L1 immunotherapy. (**B**) Subjects with a low PPP2R2A/PD-L1 ratio undergoing PD-1 immunotherapy. (**C**) Cases with a low PPP2R2A/PD-L1 ratio treated with CTLA-4 immunotherapy. (**B**-**D**) Kaplan–Meier survival curves depicting progression-free survival in cancer patients based on PPP2R2A/PD-L1 expression ratio: (**D**) Individuals with a low PPP2R2A/PD-L1 ratio receiving PD-L1 immunotherapy. (**E**) Subjects with a low PPP2R2A/PD-L1 ratio undergoing PD-1 immunotherapy. (**F**) Cases with a low PPP2R2A/PD-L1 ratio treated with CTLA-4 immunotherapy. All the data in this figure are sourced from kmplot.com. The *P*-values were calculated using the Log-rank test.

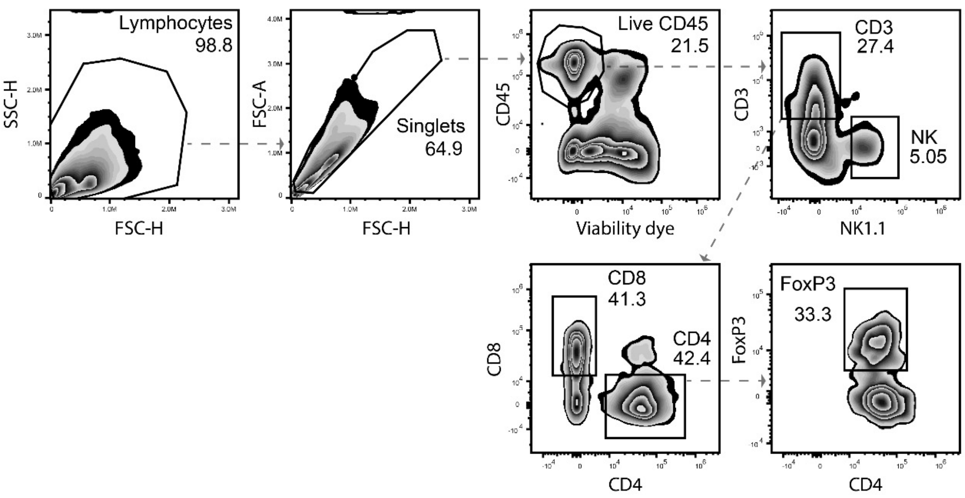

Supplementary Figure 17. Gating strategy for flow cytometry analysis.

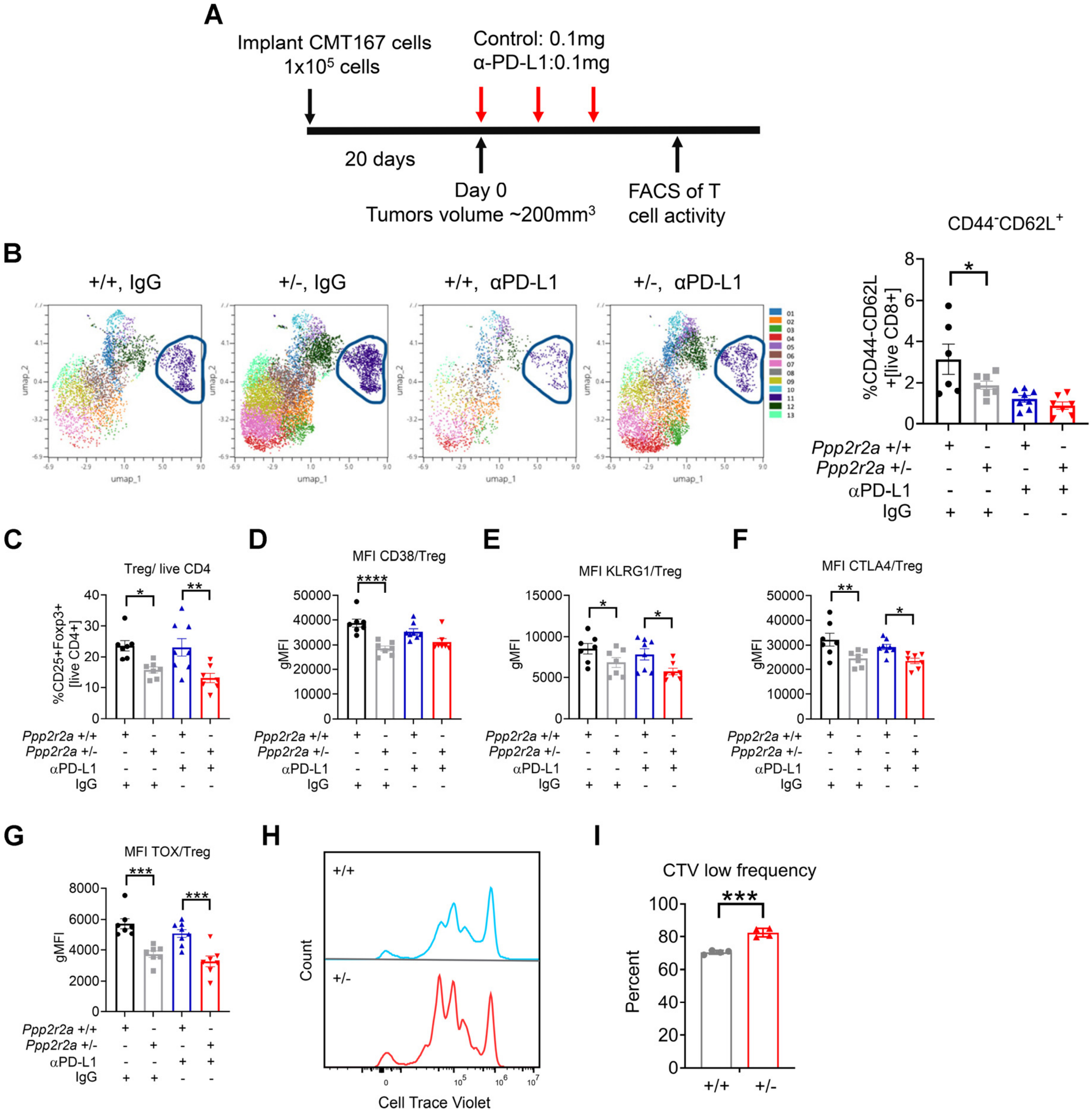

Supplementary Figure 18. ***Ppp2r2a*** **heterozygosity leads to the decreased the activity of Tregs.** (**A**) Schematic showing the schedules of control antibody and anti-PD-L1 treatment for T cell activity.1X10^5 of *Ppp2r2a*$^{+/+}$ or *Ppp2r2a*$^{+/-}$ CMT167cells into C57/B6 mice subcutaneously on the right flank. anti-PD-L1 or control antibodies every 2 days for 3 doses. Antibody started on day 20 after tumor injection. Tumor collections were performed 1day after the 3rd dose. (**B**) UMAP dimension reduction of tumor-infiltrating CD8$^+$ T cells after staining with 28 markers and spectral flow cytometry analysis. Shown is the data gated on live CD45$^+$CD3$^+$CD8$^+$ cells, subsampled on 5000 cells per sample. Unsupervised clustering analysis was done using FlowSOM algorithm with an elbow method approach for cluster number determination. The frequency of naïve T cells among groups (right). (**C-G**) Treg percentage and suppressive markers on Treg cells are different among groups. Data are shown as mean ± SEM (n = 7-8). *, $P < 0.05$, **, $P < 0.01$, ***, $P < 0.001$, ****, $P < 0.0001$, statistical significance was determined by

one-way ANOVA, followed by Bonferroni post hoc analysis for multiple comparisons (**B-G**). (**H**, **I**) Tregs co-cultured with *Ppp2r2a*$^{+/-}$ tumor cells display reduced suppressive capacity**.** (**H**) Representative histogram depicting the frequency of CTV (cell tracer violet) low $CD8^+$ T cells. $CD8^+$ T cells were activated in plates containing both Tregs and tumor cells. (I) Quantification of CTV low $CD8^+$ T cells. Data are shown as mean ± SEM (n = 4). Statistical significance was determined by Student's *t*-test; ***, $P < 0.001$.

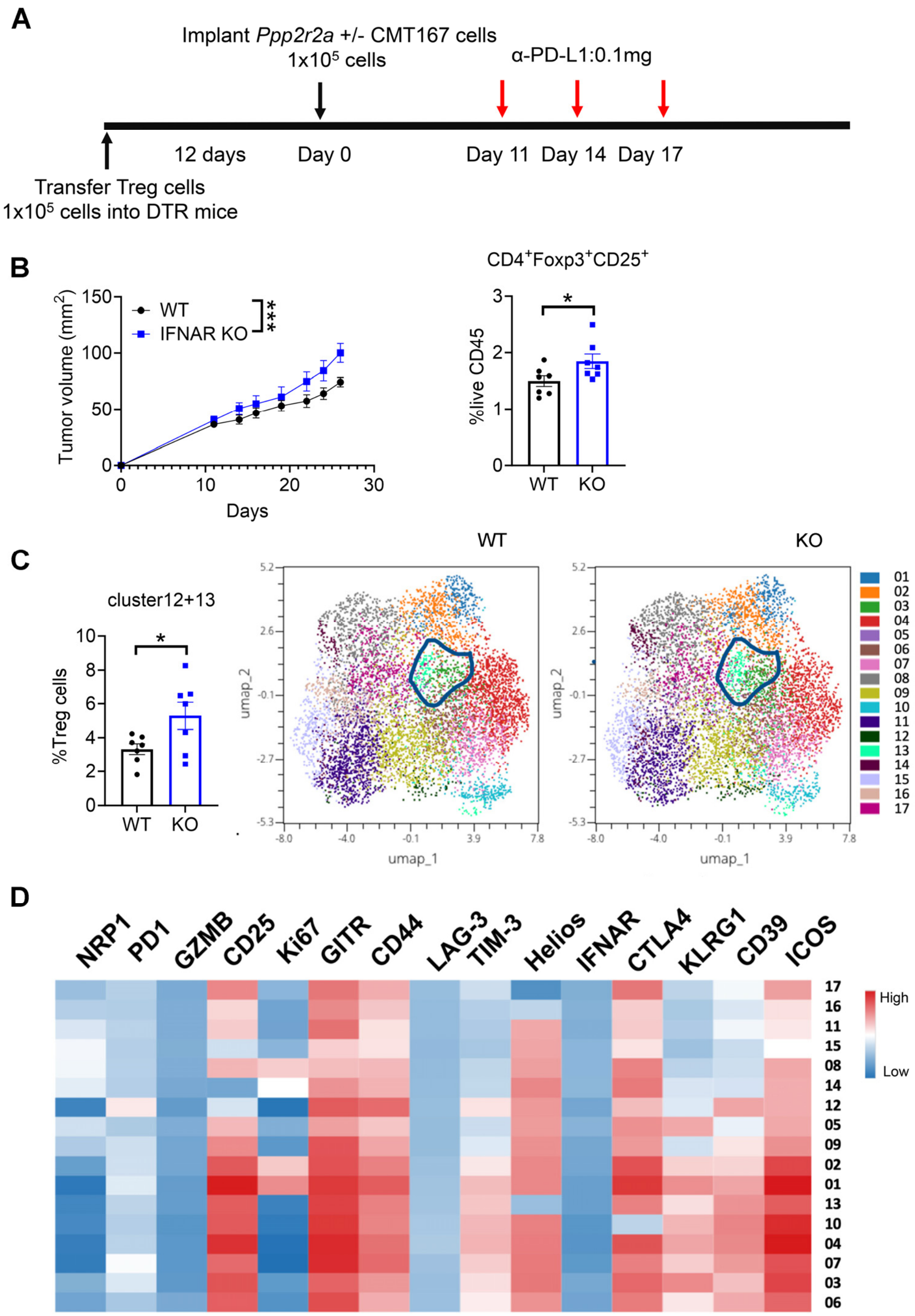

Supplementary Figure 19. *Ppp2r2a* heterozygosity enhances the efficacy of antiPD-L1 therapy in CMT-167 tumors through type I IFN signaling effects on Treg cells. (**A**) Schematic showing schedules of treatment plan. $1X10^5$ Treg cells were adoptively transferred into DTR mice on day -12. $1X10^5$ CMT167 *Ppp2r2a*$^{+/-}$ cells into C57B6 mice subcutaneously on the right flank on day 0. anti-PD-L1 antibodies were injected every 3 days for 3 doses. Antibody

started on day 11 after tumor injection. Tumor collections were performed on day 27. (**B**) The tumor growth curves of the *Ppp2r2a*$^{+/-}$ CMT167 cells with or without *Infna* knockout in Tregs (left panel). ***, *P* < 0.001, two-way ANOVA, followed by Bonferroni post hoc analysis for multiple comparisons was used to determine statistical significance. Tumor growth Frequency of $CD4^+Foxp3^+CD25^+$ cells in live $CD45^+$ cells were increased in the KO group (right panel). *P < 0.05， Student's *t*-test was used for the data analysis. (**C**) UMAP dimension reduction of tumor-infiltrating $CD4^+Foxp3^+CD25^+$ cells after staining with 33 markers and spectral flow cytometry analysis. Shown is the data gated on live $CD4^+Foxp3^+CD25^+$ cells, subsampled on 1000 cells per sample (left panel). Student's *t*-test was used for the data analysis. *P < 0.05. Unsupervised clustering analysis was done using FlowSOM algorithm with an elbow method approach for cluster number determination (right panel). The frequency of cluster 12 and 13 is increased in KO group (left). (**D**) Heatmap of **C** showing expression levels of indicated markers by each cluster.

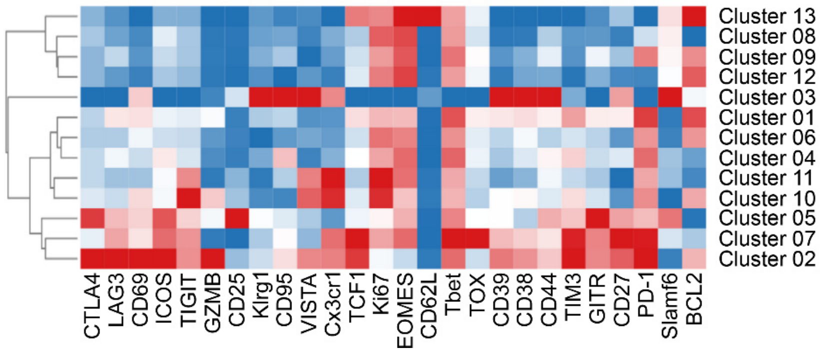

Supplementary Figure 20. Heatmap of clusters in **Figure 8B**.

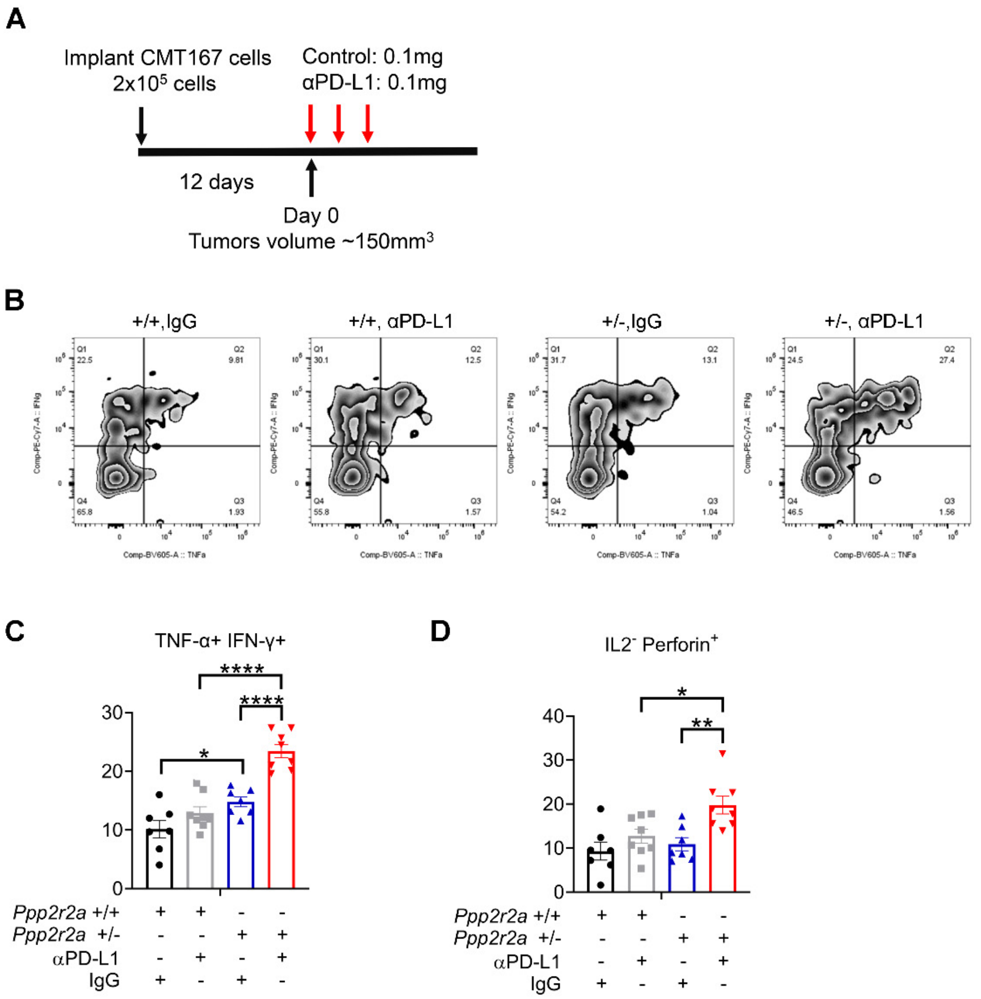

Supplementary Figure 21. CD8$^+$ T cells isolated from *Ppp2r2a*$^{+/-}$ tumors displayed increased production of IFN-γ and TNF-α. (**A**) Schematic of the treatment schedule combining *Ppp2r2a* heterozygous deletion with anti–PD-L1 therapy. (**B**) Representative flow cytometry plots of IFN-γ and TNF-α expression in CD8$^+$ T cells from the indicated groups after *ex vivo* re-stimulation. (**C**, **D**) Quantification of TNF-α$^+$ IFN-γ$^+$ (**C**) and IL-2$^-$ Perforin$^+$ (**D**) CD8$^+$ T cells. Data are mean ± SEM (n = 7–8). Statistical significance was determined by one-way ANOVA with Bonferroni's post hoc test. *, $P < 0.05$; **, $P < 0.01$; ****, $P < 0.0001$.

A

DFI										
**Parameter**		**DF**	**Parameter Estimate**	**Standard Error**	**Chi-Square**	**p-value**	**Hazard Ratio**	**95% Hazard Ratio Confidence Limits**		**Label**
**PPP2R2A_del**	**No alteration**	1	-0.29038	0.19971	2.1141	0.1459	0.748	0.506	1.106	PPP2R2A_del No alteration
**TMB**		1	0.00614	0.01179	0.2717	0.6022	1.006	0.983	1.030	

B

DSS										
**Parameter**		**DF**	**Parameter Estimate**	**Standard Error**	**Chi-Square**	**p-value**	**Hazard Ratio**	**95% Hazard Ratio Confidence Limits**		**Label**
**PPP2R2A_del**	**No alteration**	1	-0.33489	0.17912	3.4957	0.0615	0.715	0.504	1.016	PPP2R2A_del No alteration
**TMB**		1	-0.01199	0.01220	0.9655	0.3258	0.988	0.965	1.012	

Supplementary Table 1: Cox proportional hazard regression test between PPP2R2A shallow deletion (vs. No alteration) and patient disease free interval (DFI) with controlling TMB level. After adjusting for TMB, PPP2R2A shallow deletion remained associated with worse DFI, consistent with the univariate log-rank test shown in SFig2F, although the p-value shifted from 0.10 to 0.15. (**B**) Cox proportional hazard regression test between PP2R2A shallow deletion (vs. No alteration) and patient disease specific survival (DSS) with controlling TMB level. After adjusting for TMB, PPP2R2A shallow deletion remained associated with worse DSS, consistent with the univariate log-rank test shown in SFig2E, and the p-value slightly changed from 0.077 to 0.06.

Materials and methods for supplementary data:

Bioinformatics analysis

Data on *PPP2R2A* deletion and expression in patients with NSCLC were sourced from Pan-Lung cancer *PPP2R2A* putative copy-number alteration data from Cbioportal (https://www.cbioportal.org/). TCGA pan-cancer RNA-Seq data, along with patient survival information, were downloaded from the Xena browser (https://xenabrowser.net/datapages/). Overall survival and progression-free survival data based on the PPP2R2A:PD-L1 ratio were obtained from Kmplot (https://kmplot.com/) (1). All the bioinformatics analysis were completed by December 1, 2023.

RNA-sequencing and Gene Set Enrichment Analysis (GSEA)

The details of RNA-sequencing experiments in A549 cells were provided in our previous publication (2). Bulking RNA data has been deposited in NCBI GEO with the accession number GSE311238. Pathway enrichment was determined using Gene Set Enrichment Analysis (GSEA) and hallmark gene sets from the Molecular Signatures Database (MSigDB).

Glycosylation assays

To check the glycosylation of PD-L1 protein, the cell lysates were treated with PNGase F (P0704L, New England Biolabs) and Endo H (P0702L, New England Biolabs) according to the protocol provided by the manufacturer.

Treg adoptive transfer experiment

1×10^5 Treg cells (IFNAR WT or IFNAR KO Tregs) were adoptively transferred into CD11b-DTR mice (Jackson Lab, strain: 006000) on day -12. 1×10^5 CMT167 *PPP2R2A* heterozygous knockout cells were subcutaneously injected into C57BL6 mice on the right flank on day 0. 100 µg of anti-PD-L1 antibodies (Anti-Mouse PD-L1 In Vivo Antibody, Cat: ICH1086, Clone:10F.9G2, Ichorbio) were injected every 3 days for 3 doses starting from day 11. Tumor growth was monitored. Mice were euthanized and tumors were collected on day 27. T cell exhaustion panel (**Supplementary Table 6**) was then analyzed. Tissue digestion, cell isolation and flow cytometry were as previously described (3).

Treg suppression assay with CellTrace Violet

To evaluate the suppressive activity of Tregs on $CD8^+$ T cells, splenic Tregs were isolated using the Treg Isolation Kit (STEMCELL Technologies, Cat. #19863) and cultured in a transwell plate pre-seeded with tumor cells one day prior. After 24 hours, $CD8^+$ T cells were purified from spleens using the $CD8^+$ T Cell Isolation Kit (STEMCELL Technologies, Cat. #19853), labeled with CellTrace Violet (CTV; Invitrogen, Cat. #C34557) and added to the upper transwell insert containing Tregs. $CD8^+$ T cells were stimulated with soluble anti-CD3 (3 µg/ml; BioLegend, Cat. #317326), anti-CD28 (1 µg/ml; BioLegend, Cat. #302914), Perforin (BioLegend Cat #154310) and IL-2 (50 U/ml; BioLegend, Cat. #503824) when they were added. Following 3 days of co-culture, cells were harvested and analyzed by flow cytometry.

Detection of TNF-α and IFN-γ

To quantify the TNF-α and IFN-γ, the single-cell suspensions were prepared from the indicated tumor samples and plated in U-bottom 96-well plates. Cells were stimulated with Cell Stimulation Cocktail (eBioscience, Cat. #00-4970-93) for 3 hours, followed by staining with a Fixable Viability Dye (BioLegend, Cat. #423105) and surface markers. After fixation and permeabilization for 1 hour using the Fixation/Permeabilization Solution Kit (BioLegend, Cat. #421002), cells were stained with an antibody cocktail containing anti-mouse TNF-α (BioLegend, Cat. #506329) and anti-mouse IFN-γ (BioLegend, Cat. #505829) for 2 hours in permeabilization buffer and analyzed by flow cytometry.

ELISA

Cytokine concentrations of IFN-α and IFN-β were measured using commercially available ELISA kits according to the manufacturers' instructions: Human IFN-β ELISA Kit (R&D Systems, DY814), Mouse IFN-α ELISA Kit (PBL Assay Science, 42115), and Mouse IFN-β ELISA Kit (R&D Systems, DY8234).

1. Gyorffy B. Integrated analysis of public datasets for the discovery and validation of survival-associated genes in solid tumors. *Innovation (Camb)*. 2024;5(3):100625.
2. Qiu Z, Fa P, Liu T, Prasad CB, Ma S, Hong Z, et al. A genome-wide pooled shRNA screen identifies PPP2R2A as a predictive biomarker for the response to ATR and CHK1 inhibitors. *Cancer research*. 2020.
3. Zhou L, Velegraki M, Wang Y, Mandula JK, Chang Y, Liu W, et al. Spatial and functional targeting of intratumoral Tregs reverses CD8+ T cell exhaustion and promotes cancer immunotherapy. *J Clin Invest*. 2024;134(14).
