# Supplementary material for: PPP2R2A insufficiency enhances PD-L1 immune checkpoint blockade efficacy in lung cancer through cGAS-STING activation
Source: J Clin Invest. 2025 Dec 18;136(4):e193354. doi: 10.1172/JCI193354 (PMC12904717; doi:10.1172/JCI193354)

Full unedited gel for Figure 1A

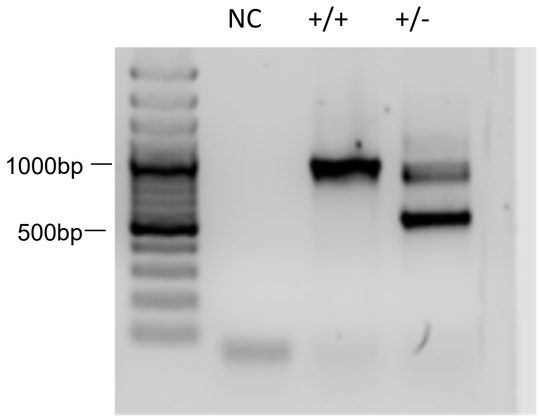

CMT167 PCR

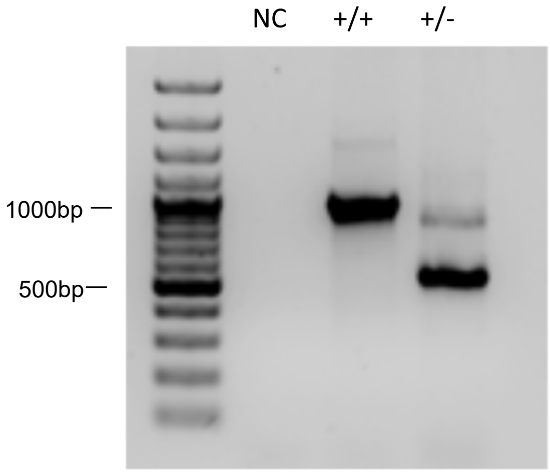

LLC PCR

Full unedited blot for Figure 1B CMT167

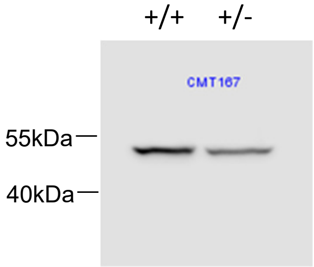

PP2A B55α

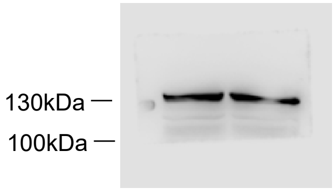

Flag Cas9

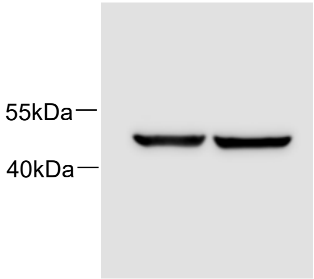

β-Actin

(loading control of PP2A B55α and Flag Cas9)

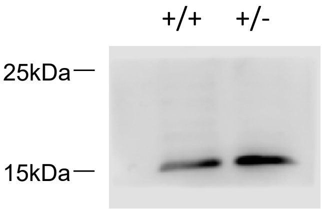

γ-H2AX

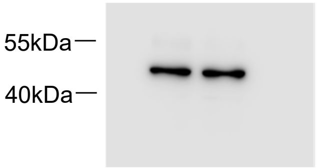

β-Actin

(loading control of γ-H2AX)

Full unedited blot for Figure 1B LLC

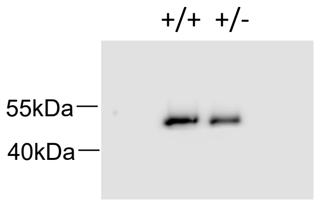

PP2A B55α

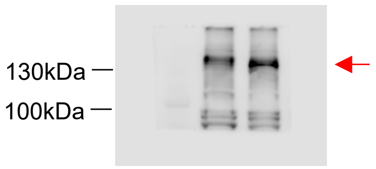

Flag Cas9

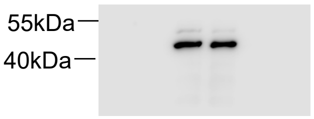

β-Actin
(loading control of PP2A B55α and Flag Cas9)

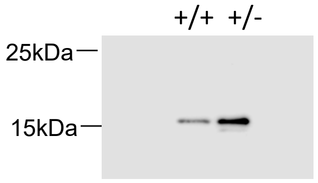

γ-H2AX

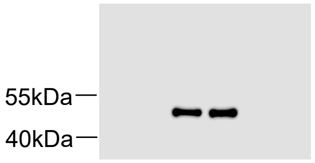

β-Actin
(loading control of γ-H2AX)

Full unedited blot for Figure 1F CMT167

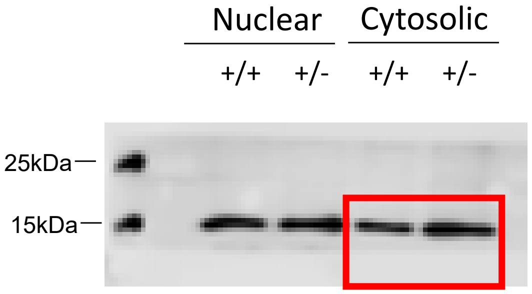

Histone H3 (Cytosolic fraction)

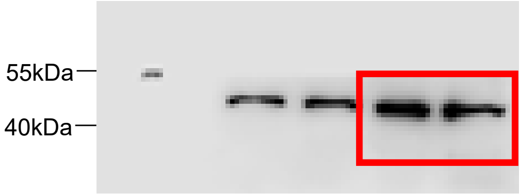

β-Actin (Cytosolic fraction)

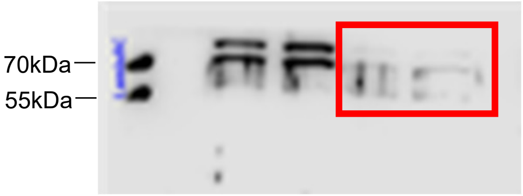

Lamin A/C (Cytosolic fraction)

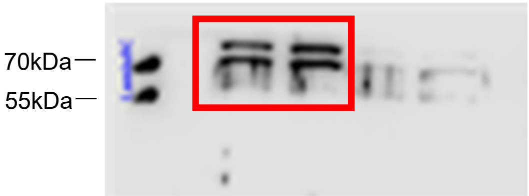

Lamin A/C (Nuclear fraction)

Full unedited blot for Figure 2A CMT167

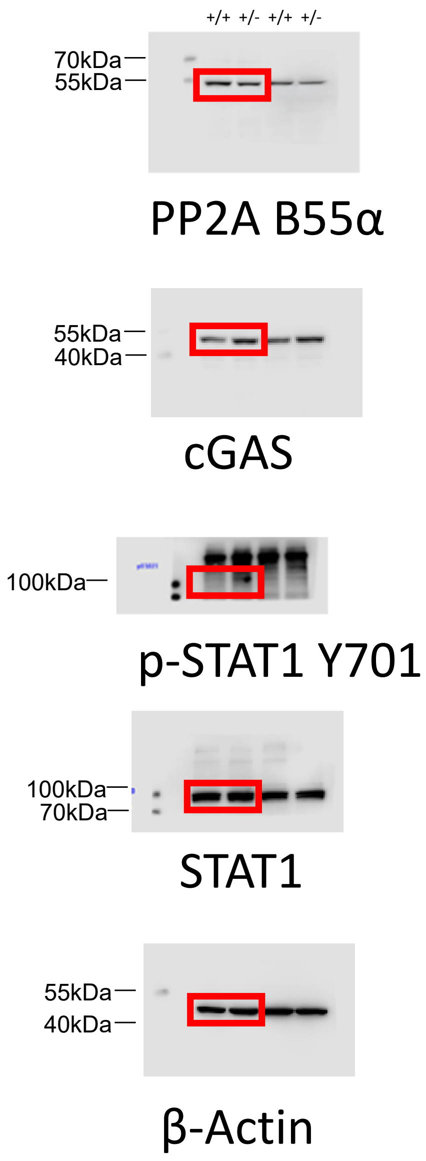

(loading control of PP2A B55α
, cGAS, pSTAT1 and STAT1)

Full unedited blot for Figure 2A CMT167

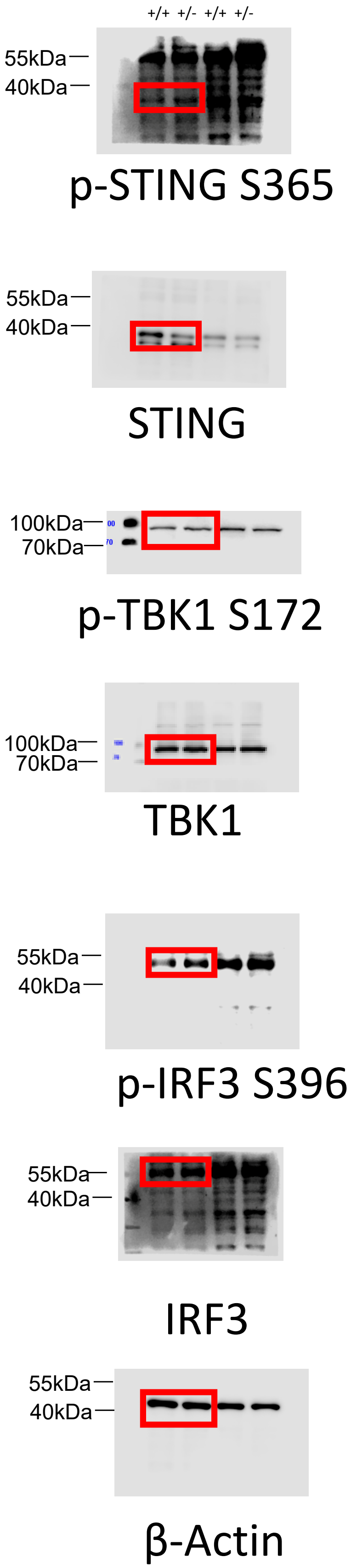

(loading control of pSTING, STING, pTBK1, TBK1, pIRF3 and IRF3)

Full unedited blot for Figure 2A LLC

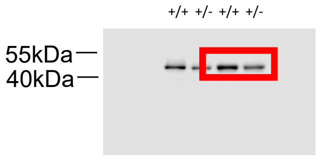

PP2A B55α

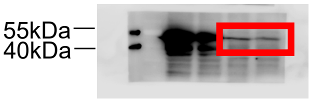

cGAS

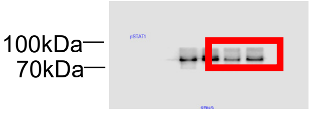

p-STAT1 Y701

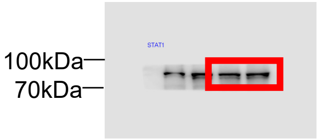

STAT1

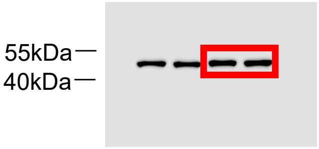

β-Actin

(loading control of PP2A B55α , cGAS, pSTAT1 and STAT1)

Full unedited blot for Figure 2A LLC

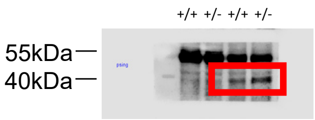

p-STING S365

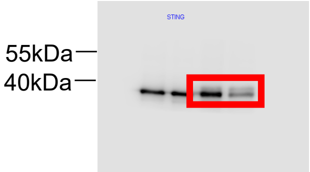

STING

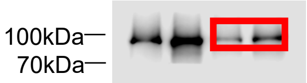

p-TBK1 S172

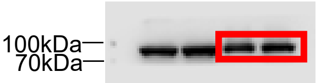

TBK1

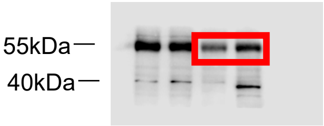

p-IRF3 S396

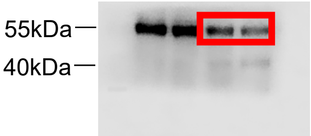

IRF3

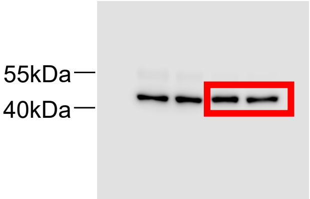

β-Actin
(loading control of pSTING, STING, pTBK1, TBK1, pIRF3 and IRF3)

Full unedited blot for Figure 2D A549

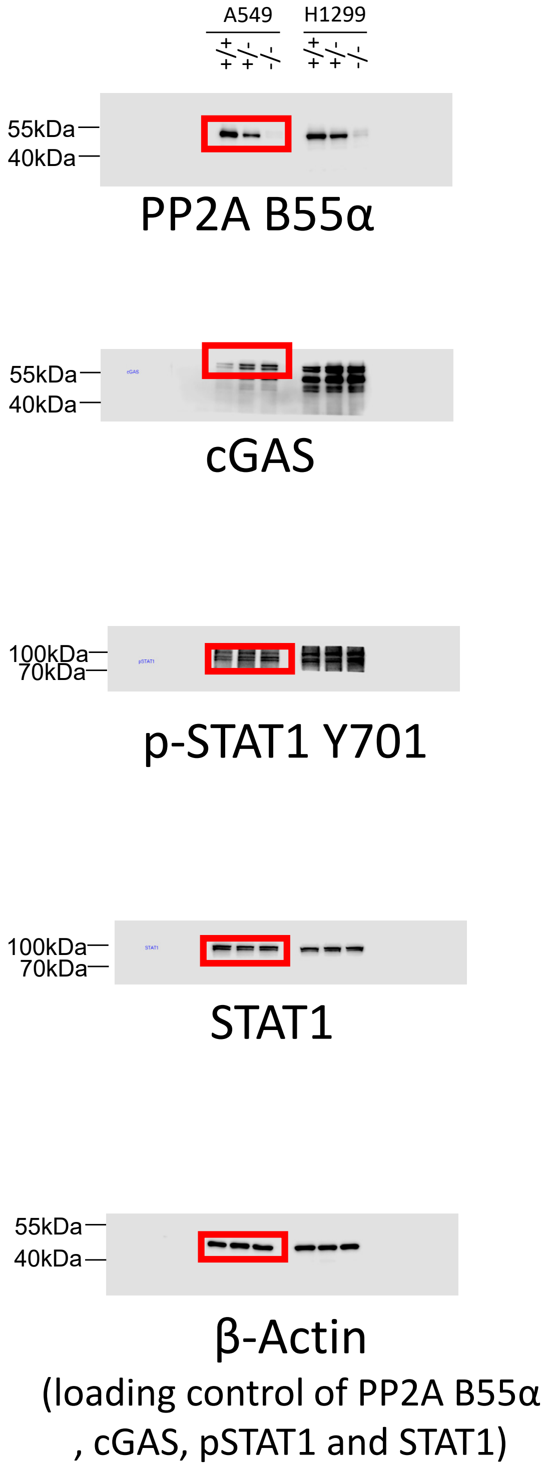

Full unedited blot for Figure 2D A549

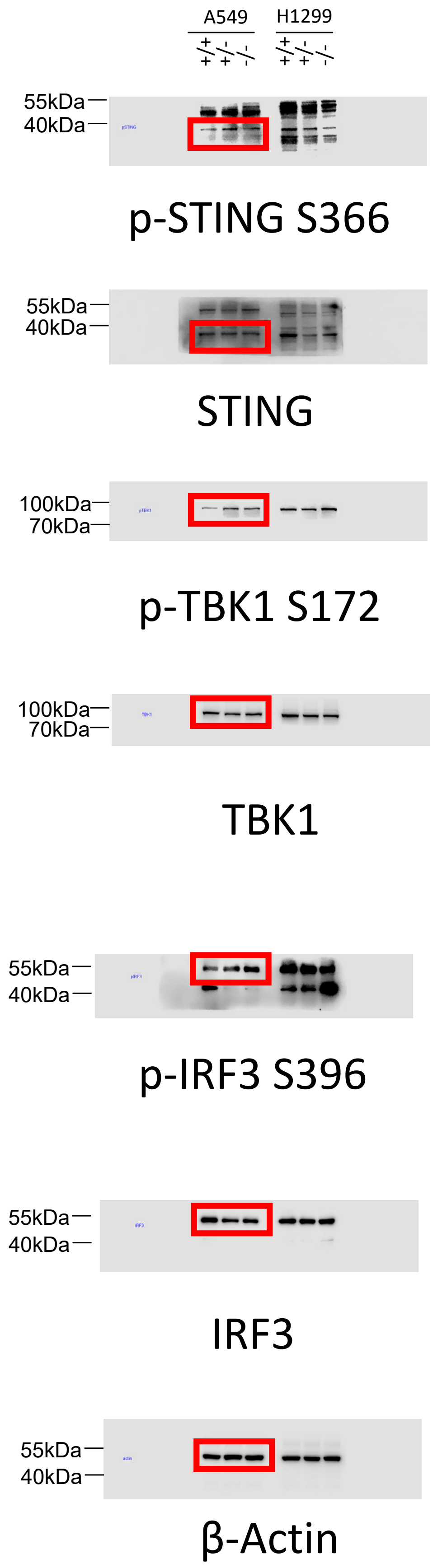

(loading control of pSTING, STING, pTBK1, TBK1, pIRF3 and IRF3)

Full unedited blot for Figure 2D H1299

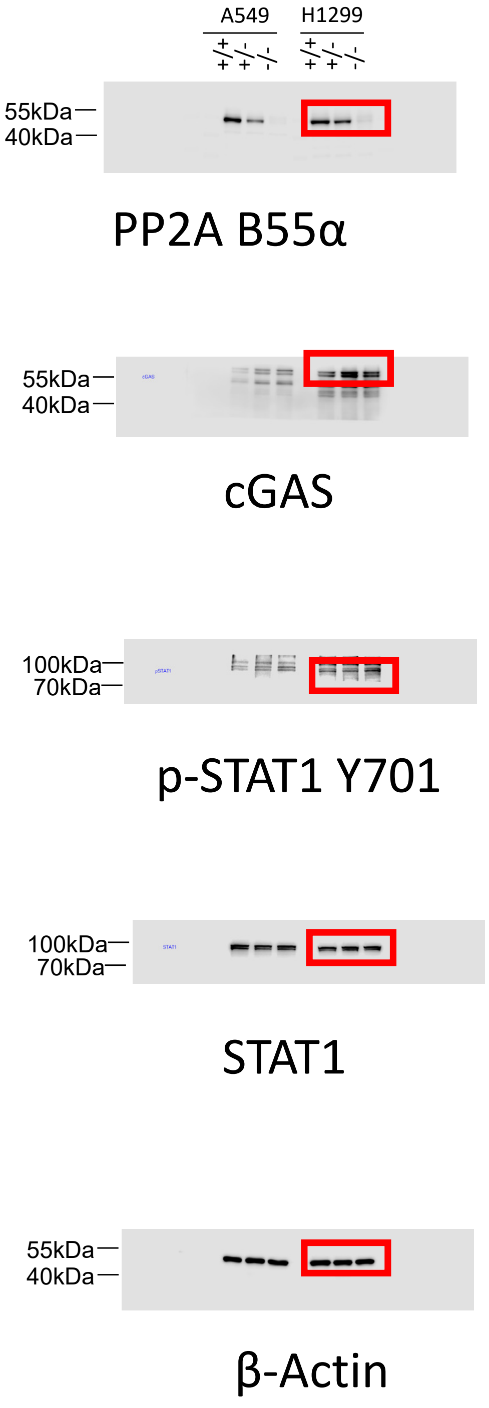

(loading control of PP2A B55α , cGAS, pSTAT1 and STAT1)

Full unedited blot for Figure 2D H1299

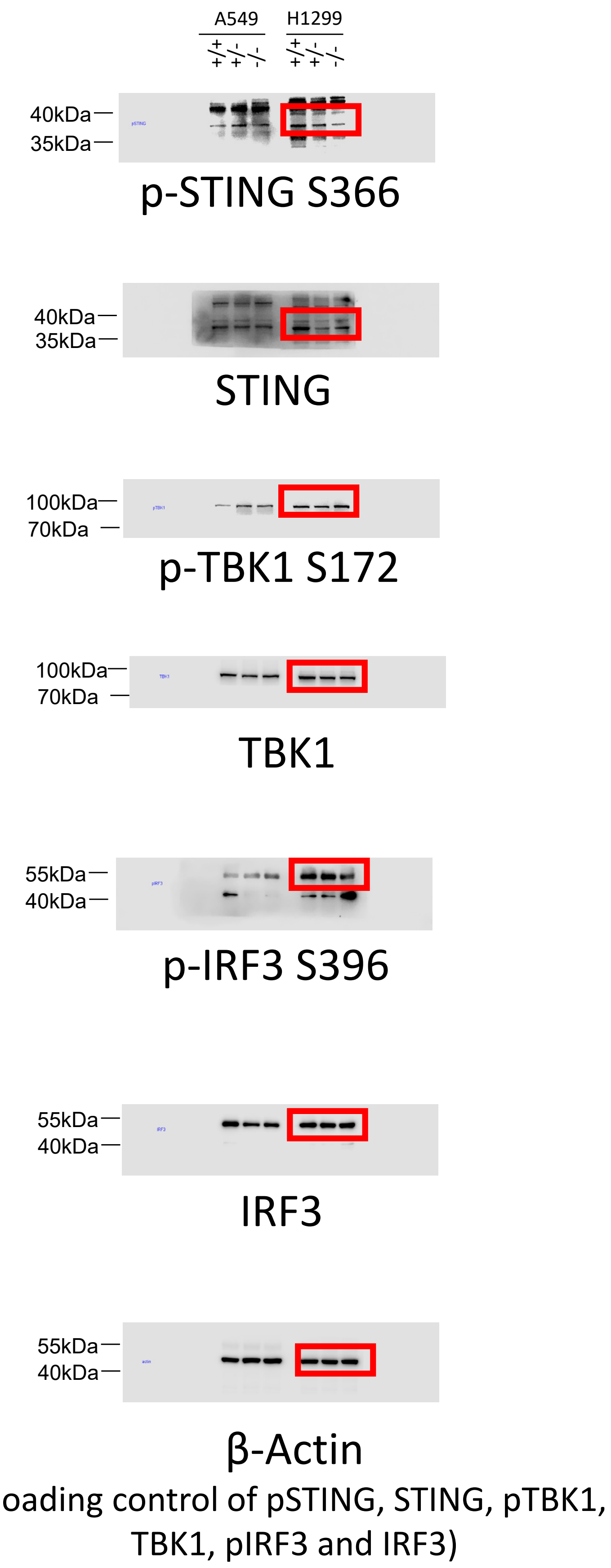

Full unedited blot for Figure 3A CMT167

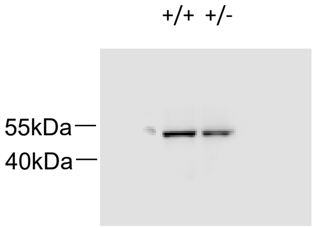

PP2A B55α

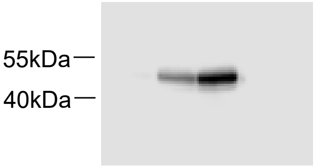

PD-L1

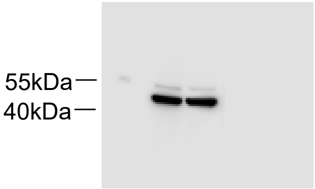

β-Actin

(loading control of PP2A B55α and PD-L1)

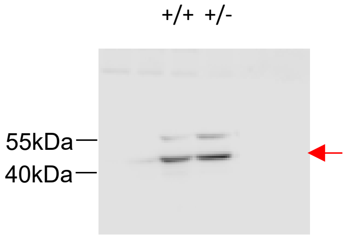

p-GSK-3β Ser9

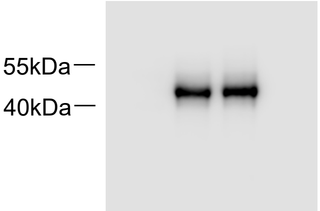

GSK-3β

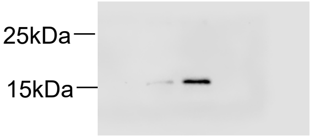

γ-H2AX

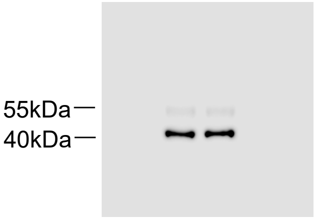

β-Actin

(loading control of p-GSK-3β, GSK-3β and γ-H2AX)

Full unedited blot for Figure 3A LLC

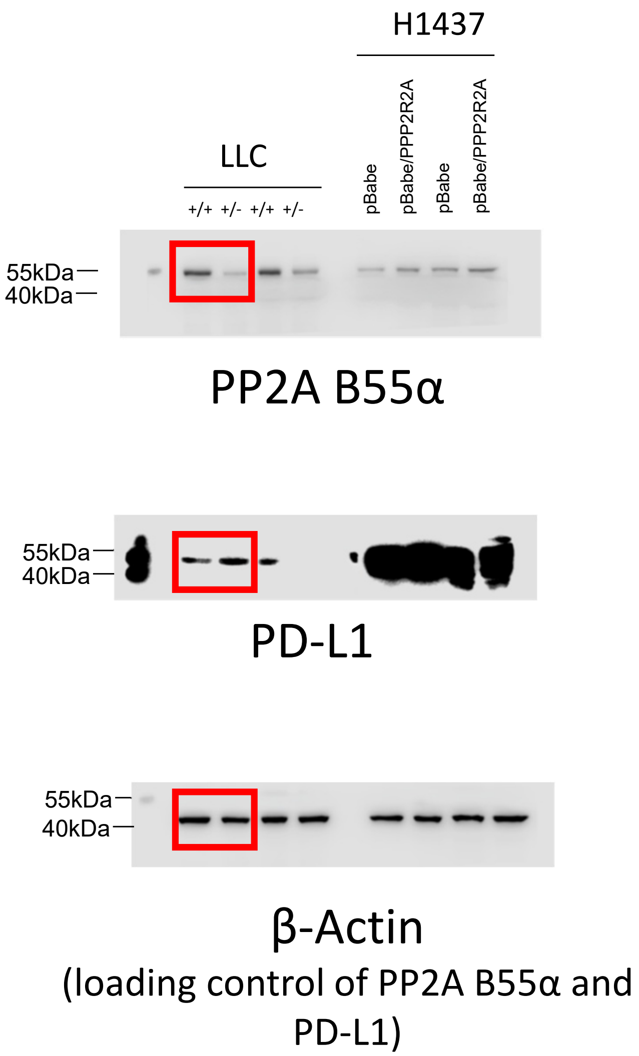

Full unedited blot for Figure 3A LLC

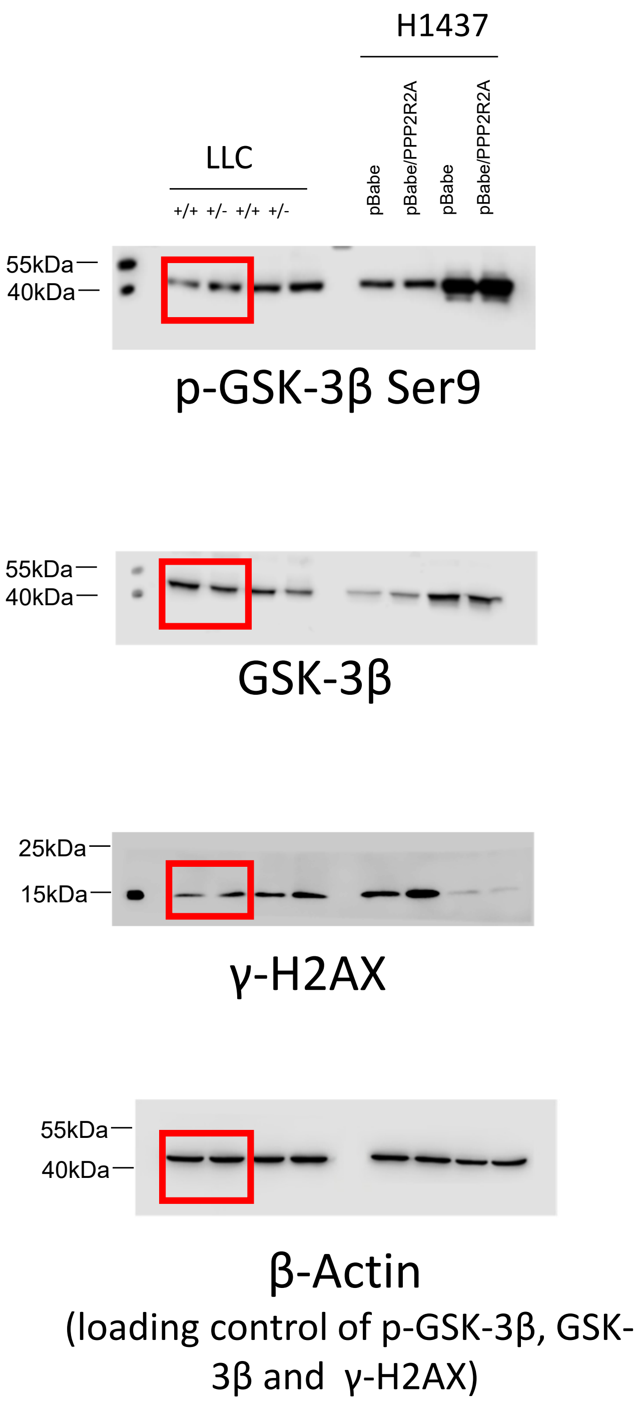

Full unedited blot for Figure 3C A549

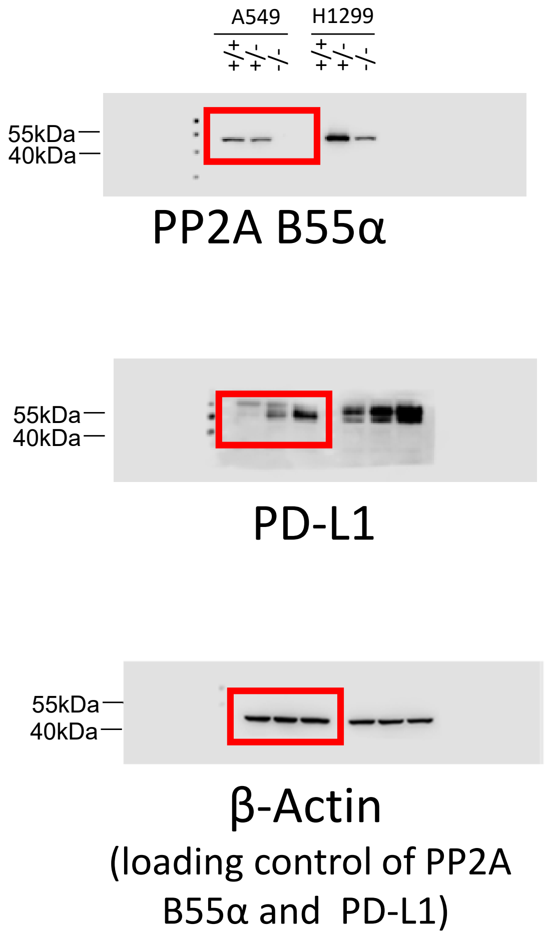

Full unedited blot for Figure 3C A549

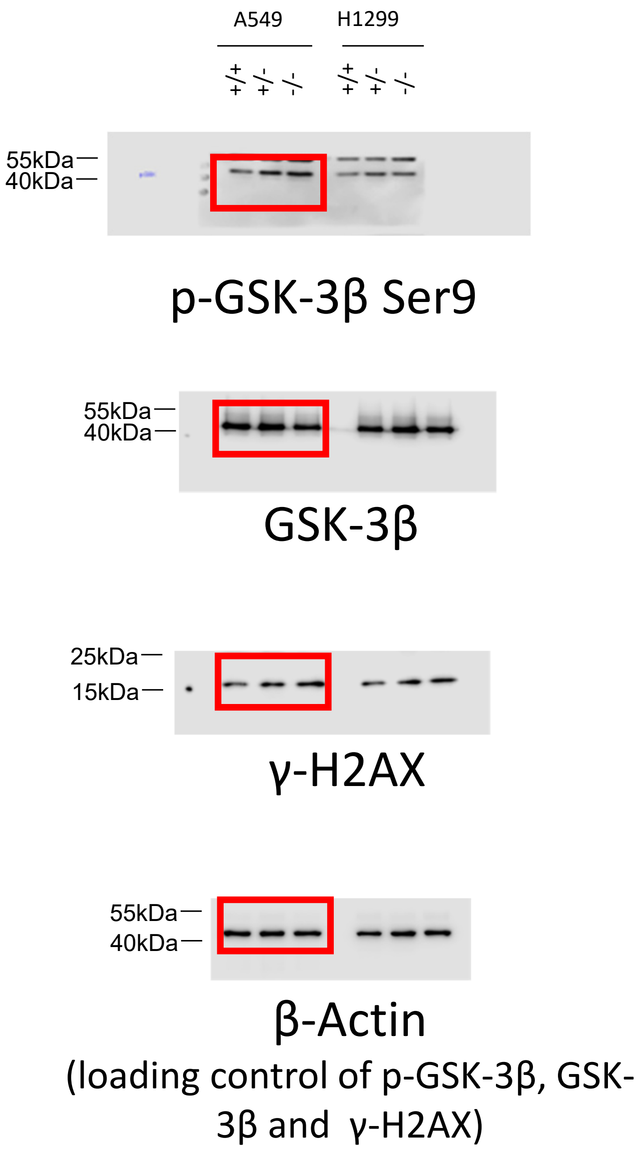

Full unedited blot for Figure 3C H1299

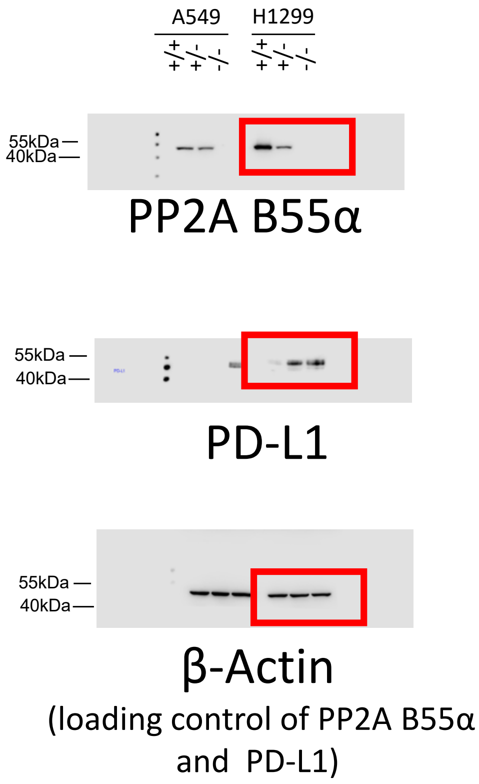

Full unedited blot for Figure 3C H1299

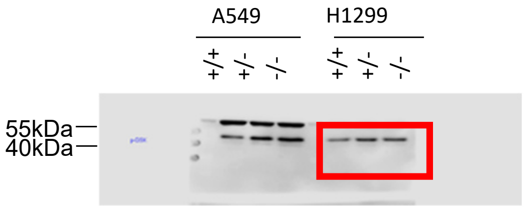

p-GSK-3β Ser9

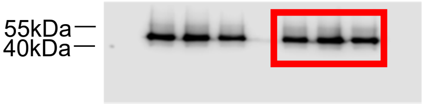

GSK-3β

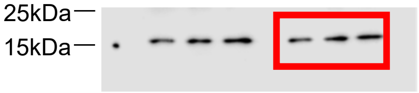

γ-H2AX

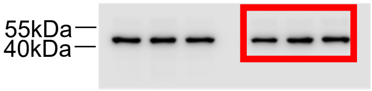

β-Actin (loading control of p-GSK-3β, GSK-3β and γ-H2AX)

Full unedited blot for Fig 3E CMT167

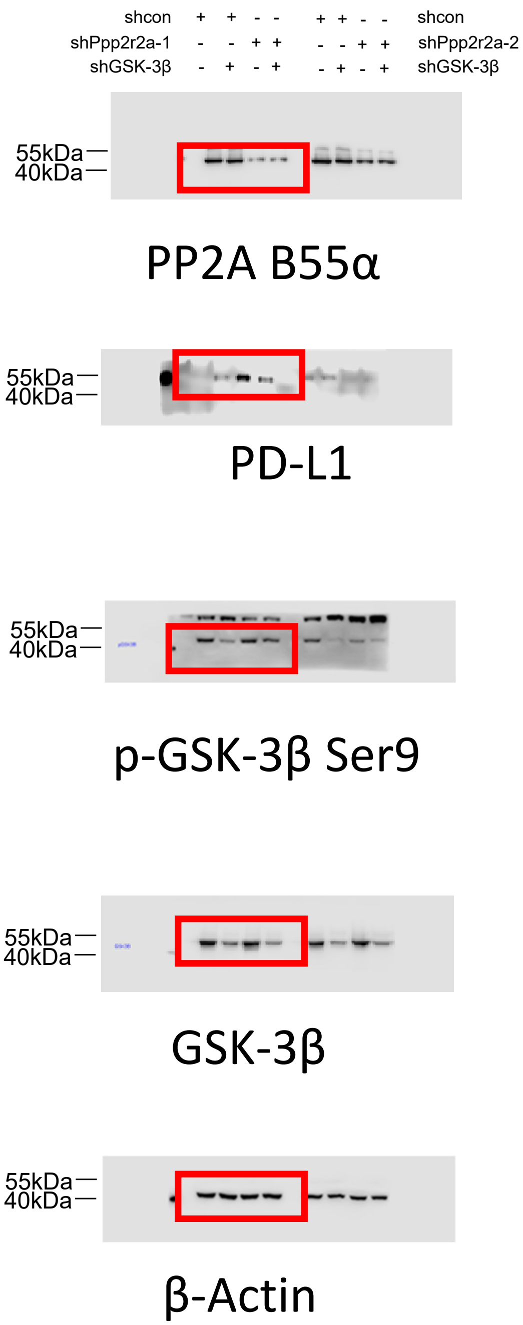

Full unedited blot for Fig 3F A549

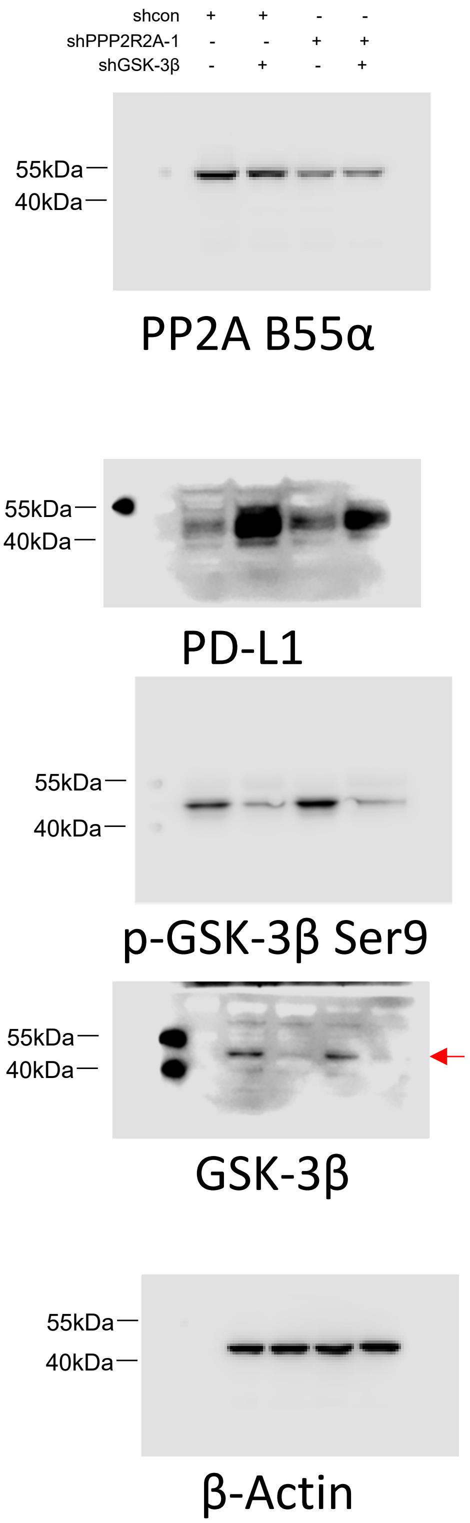

Full unedited blot for Fig3G A549

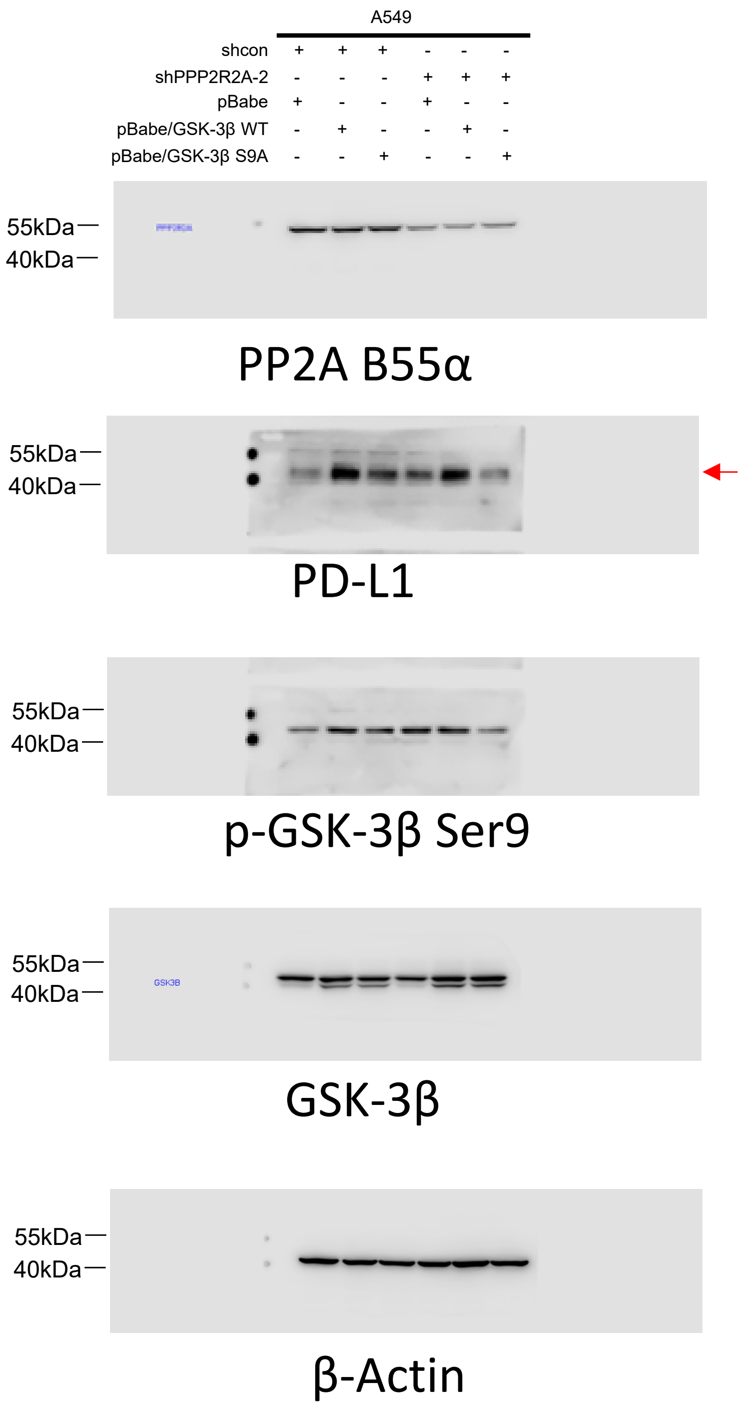

Full unedited blot for Fig3J CMT167

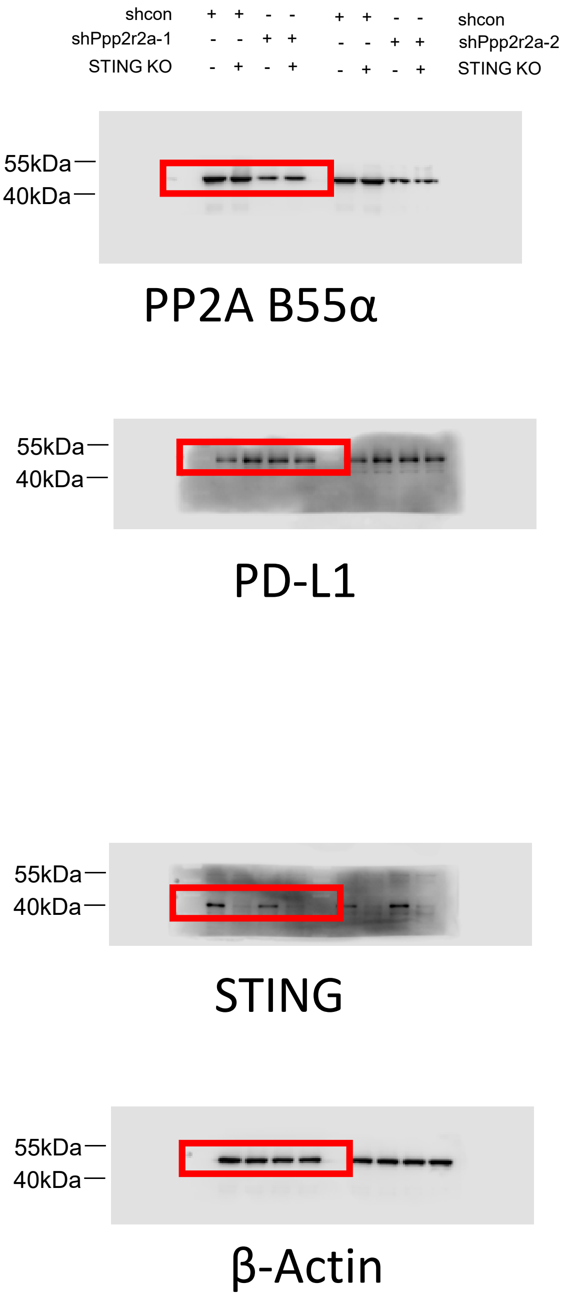

Full unedited blot for Fig3L A549

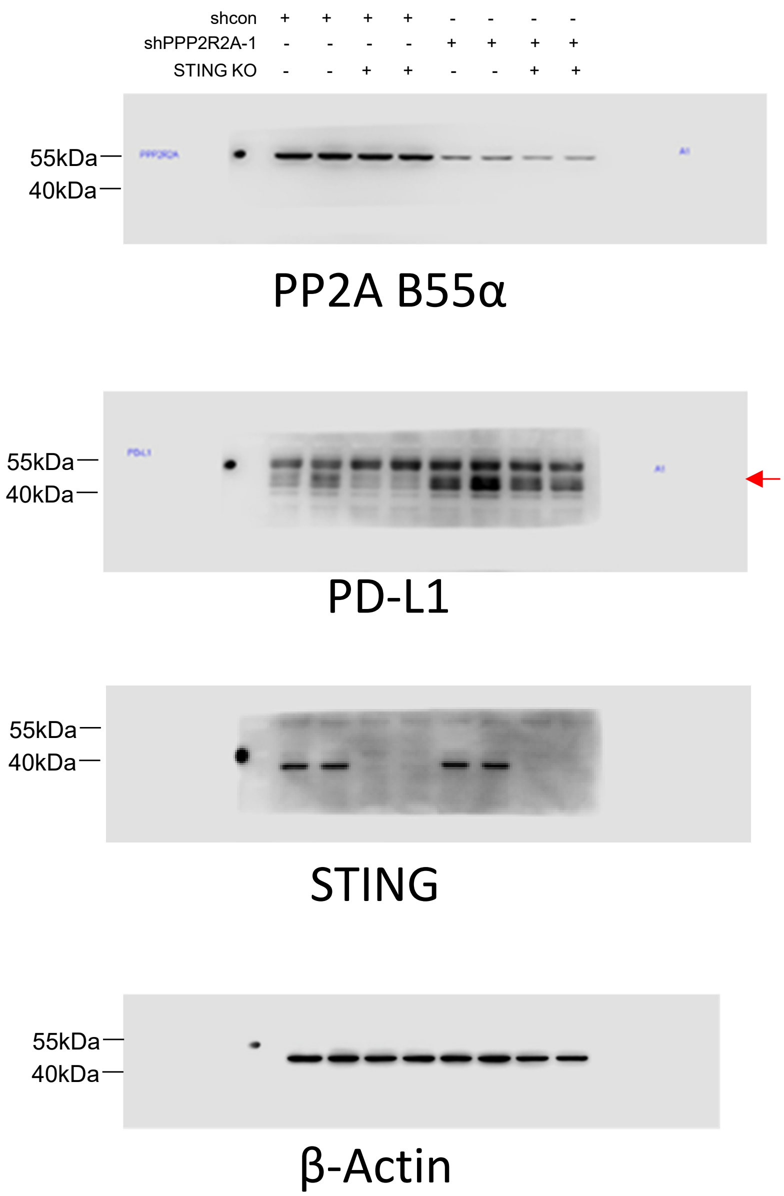

Full unedited blot for Sup Fig 1A A549

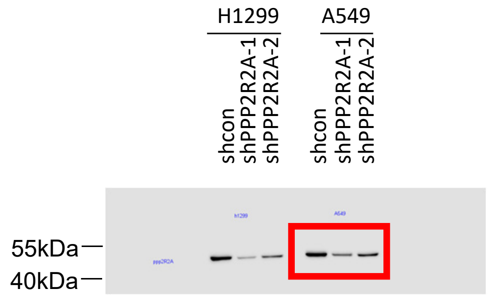

PP2A B55α

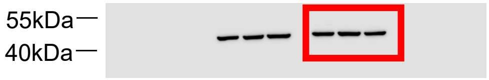

β-Actin

Full unedited gel Sup Fig 4A

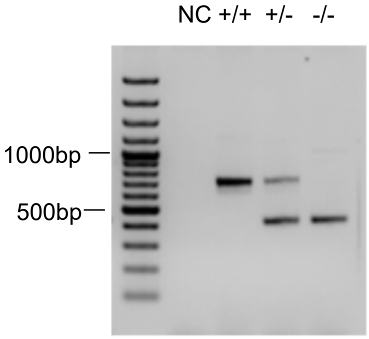

A549 PCR

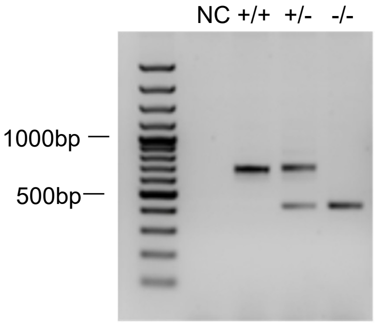

H1299 PCR

Full unedited blot for Sup Fig 4B A549

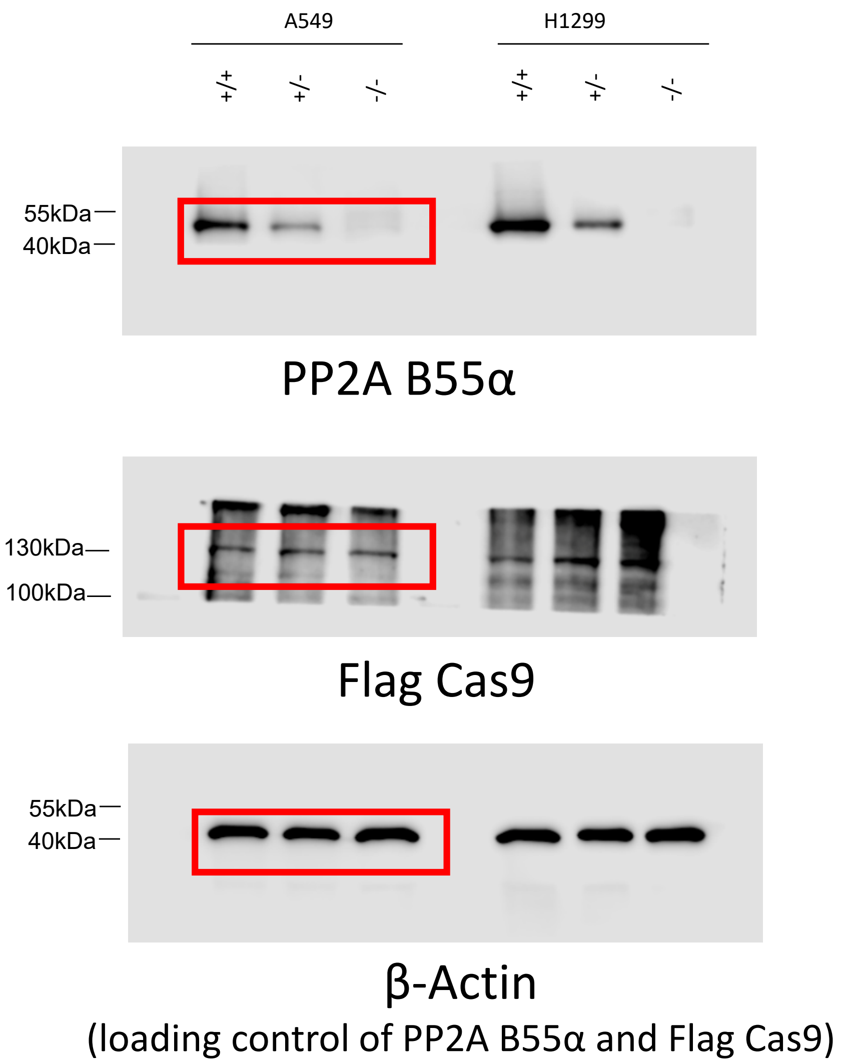

Full unedited blot for Sup Fig 4B A549

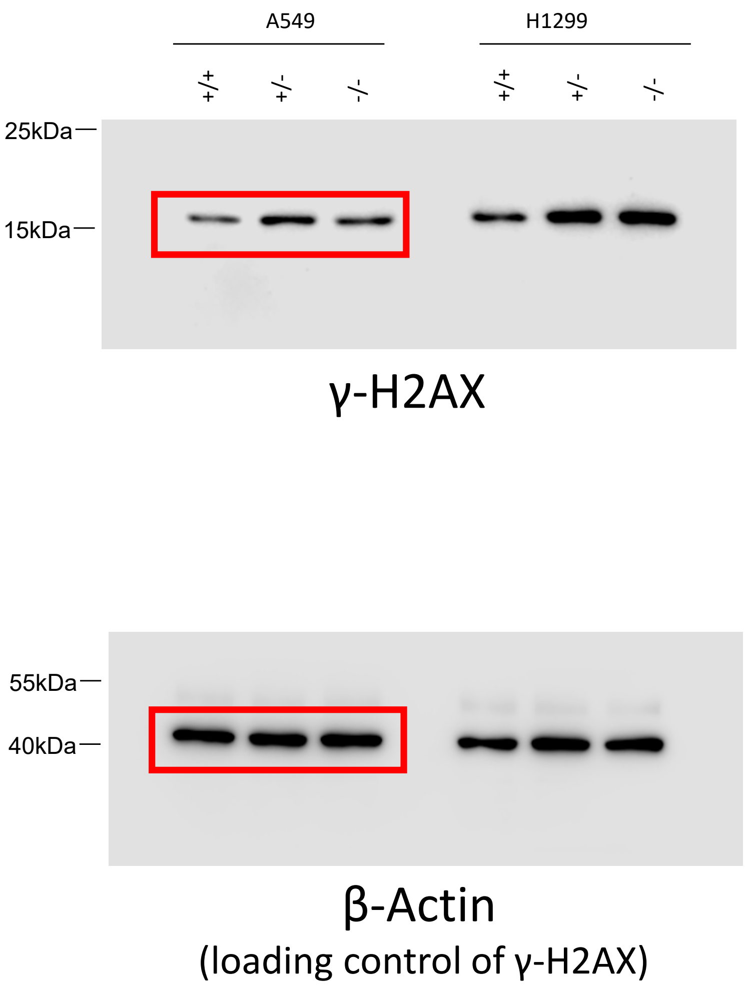

Full unedited blot for Sup Fig 4B H1299

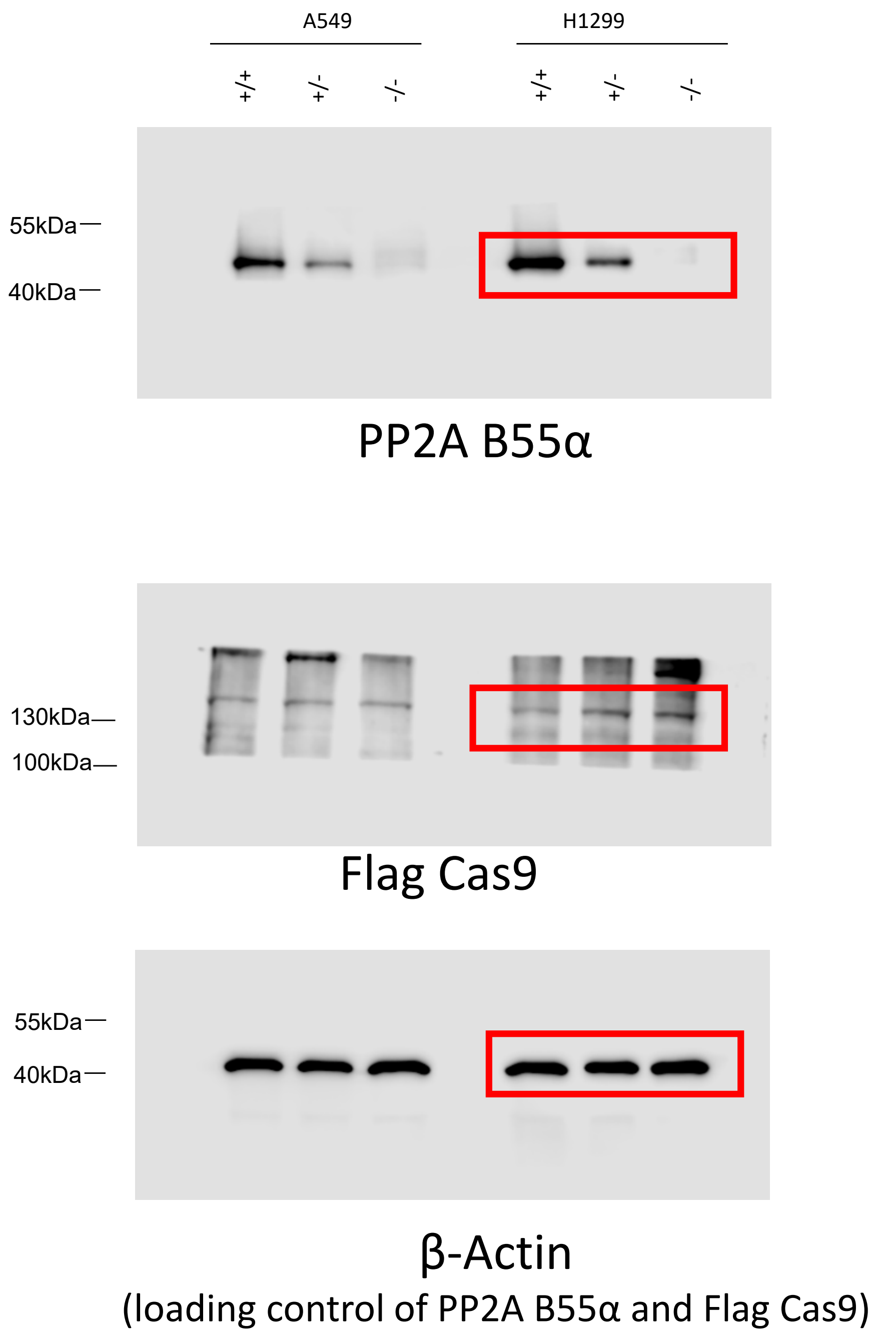

Full unedited blot for Sup Fig 4B H1299

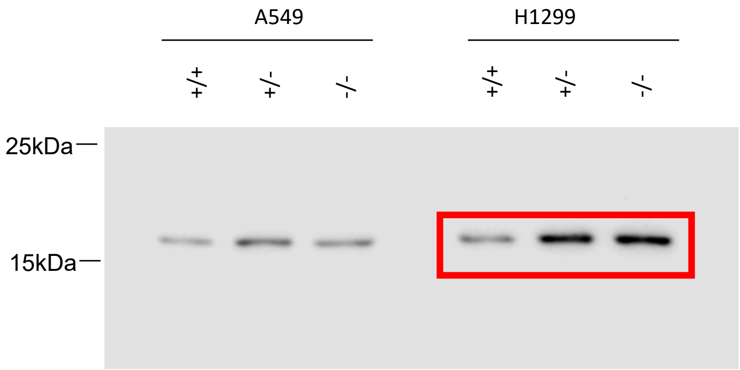

γ-H2AX

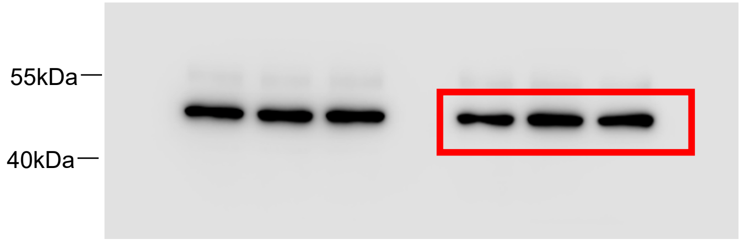

β-Actin
(loading control of γ-H2AX)

Full unedited blot for Sup Fig 5A CMT167

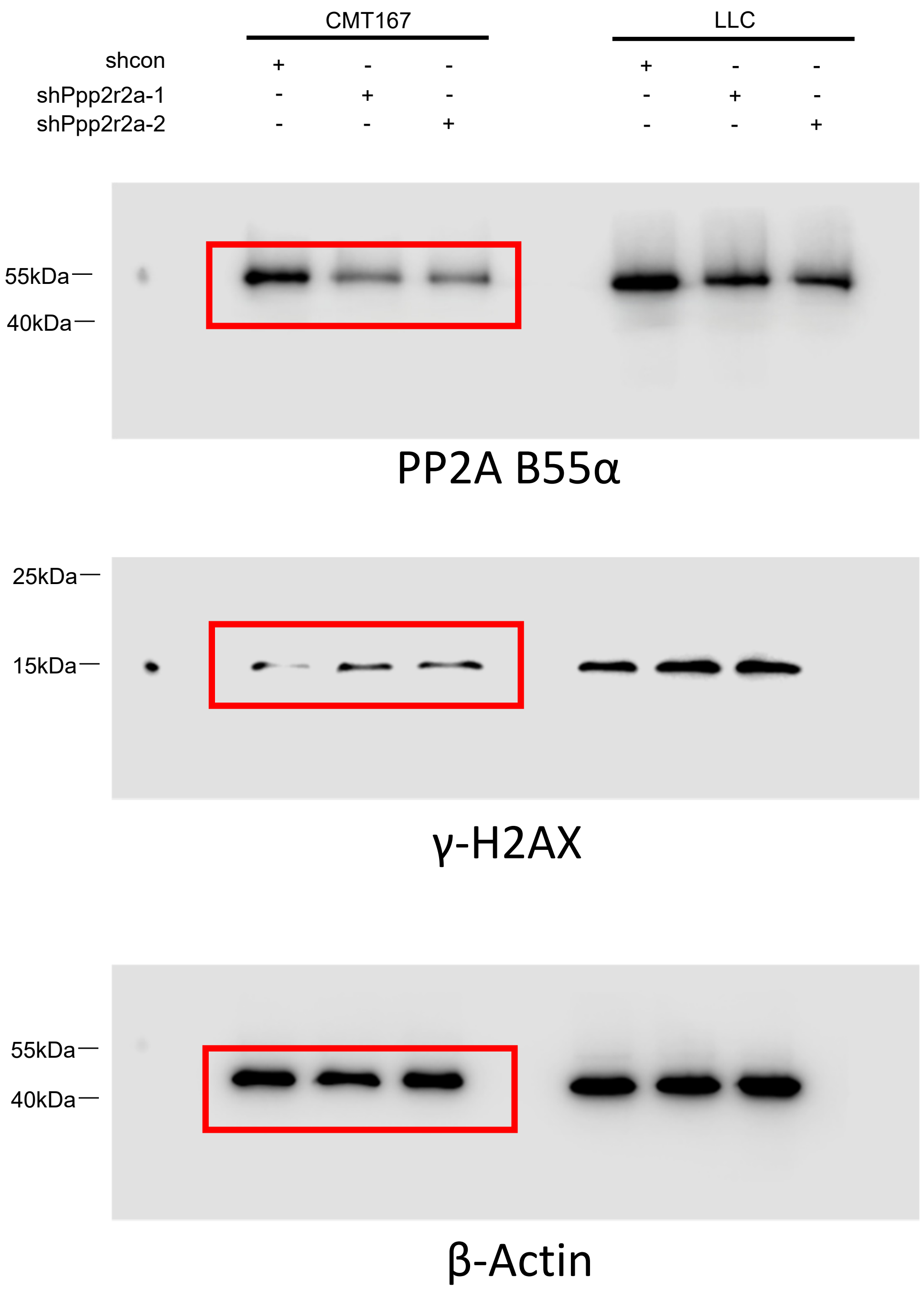

Full unedited blot for Sup Fig 5A LLC

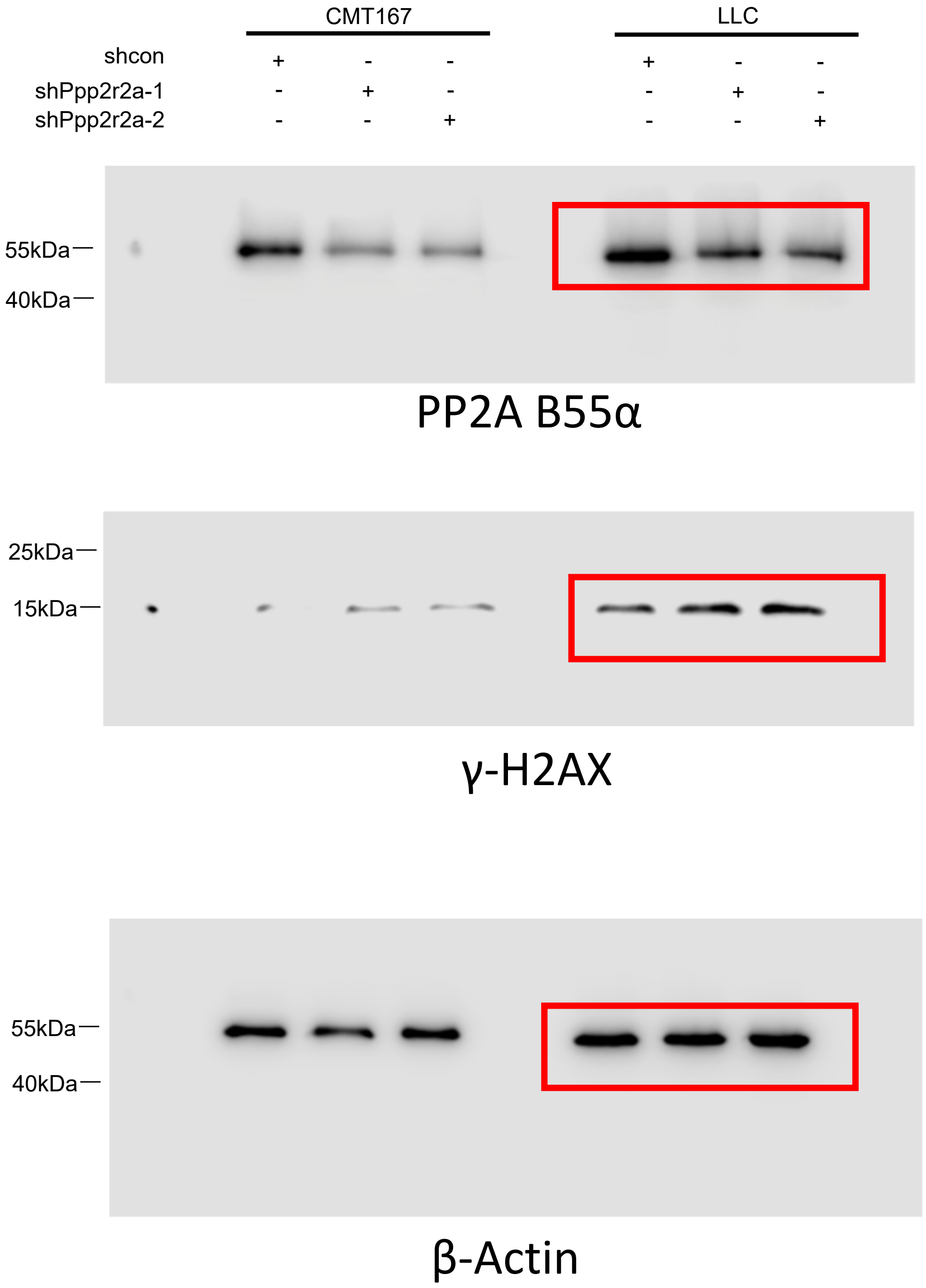

Full unedited blot for Sup Fig 5E CMT167

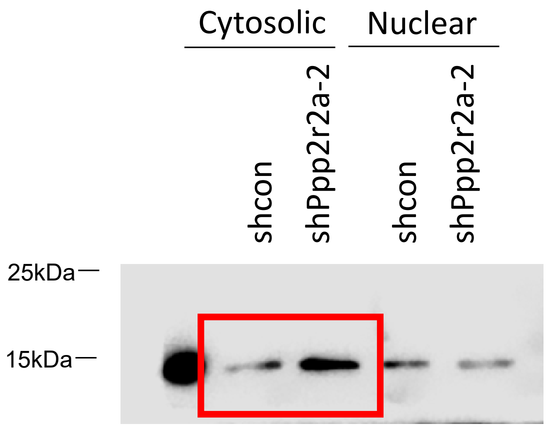

Histone H3 (Cytosolic fraction)

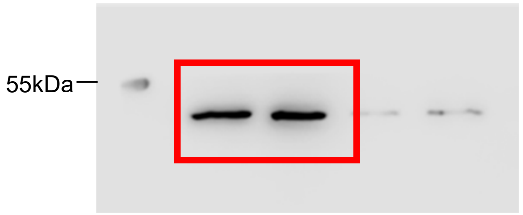

β-Actin (Cytosolic fraction)

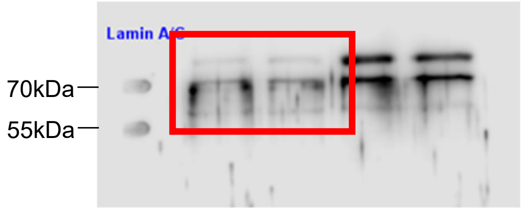

Lamin A/C (Cytosolic fraction)

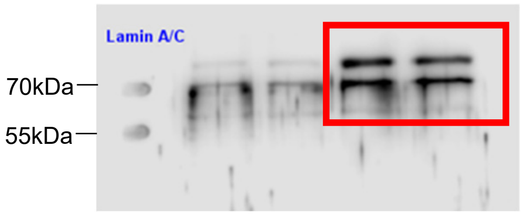

Lamin A/C (Nuclear fraction)

Full unedited blot for Sup Fig6A A549

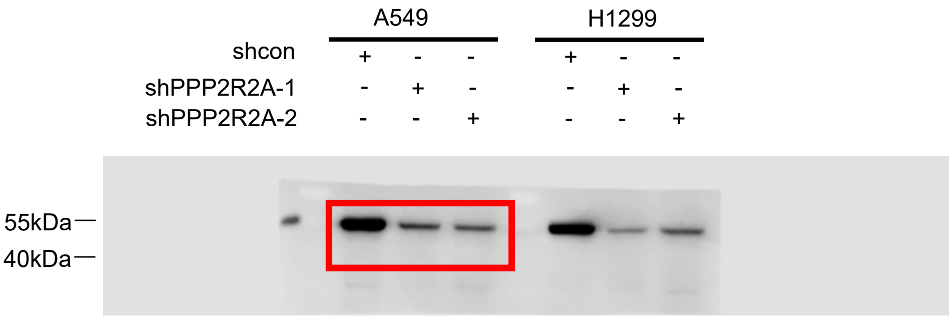

PP2A B55α

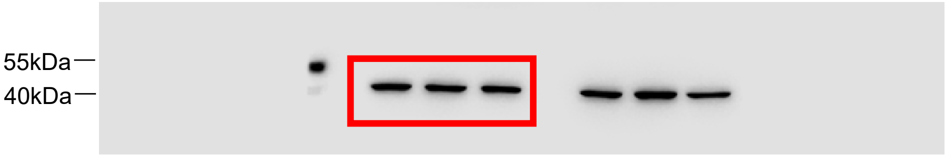

β-Actin
(loading control of PP2A B55α)

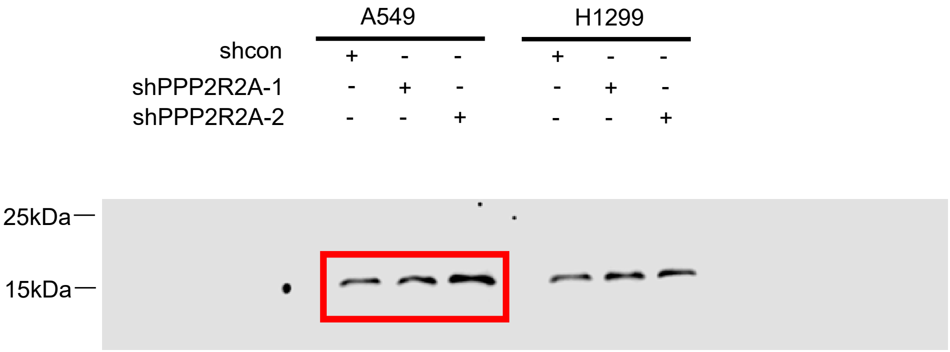

γ-H2AX

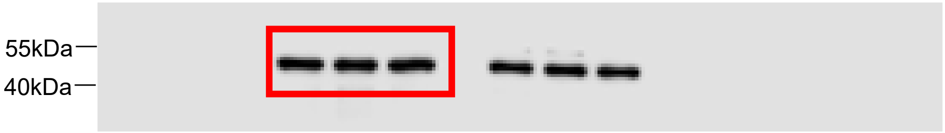

β-Actin (loading control of γ-H2AX)

Full unedited blot for Sup Fig6A H1299

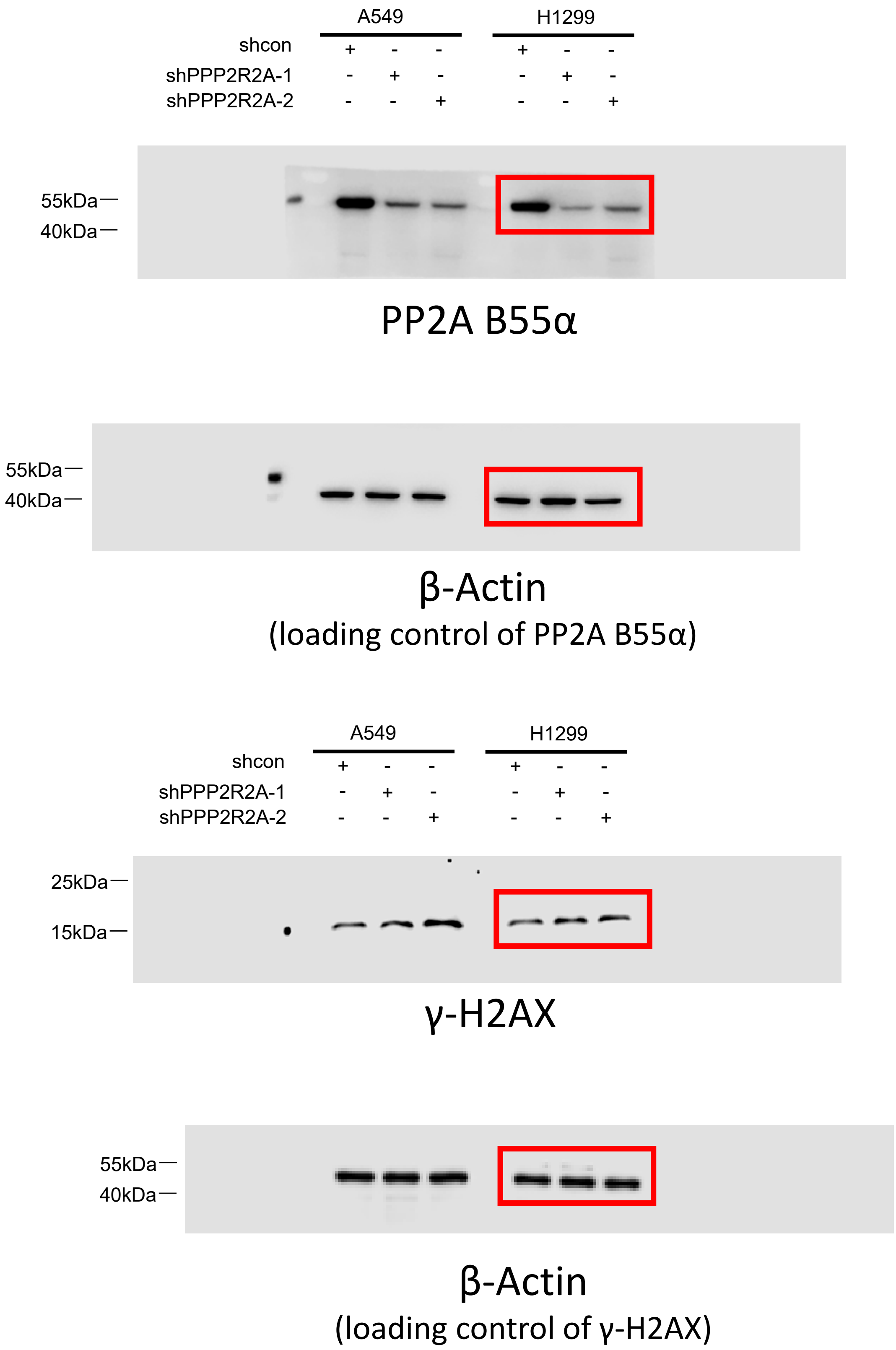

Full unedited blot for Sup Fig7

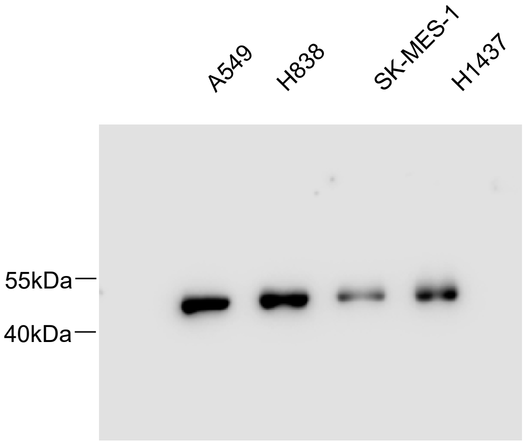

PP2A B55α

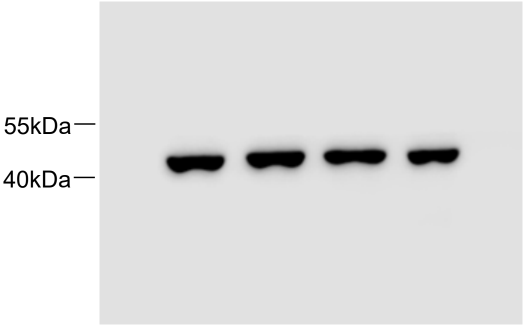

β-Actin

Full unedited blot for Sup Fig8A CMT167

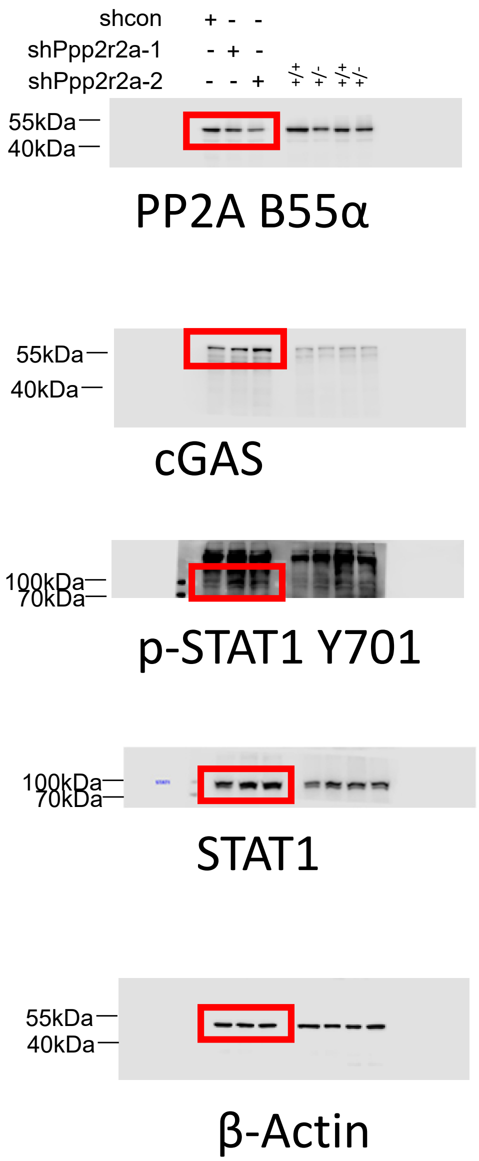

(loading control of PP2A B55α
, cGAS, pSTAT1 and STAT1)

Full unedited blot for Sup Fig8A CMT167

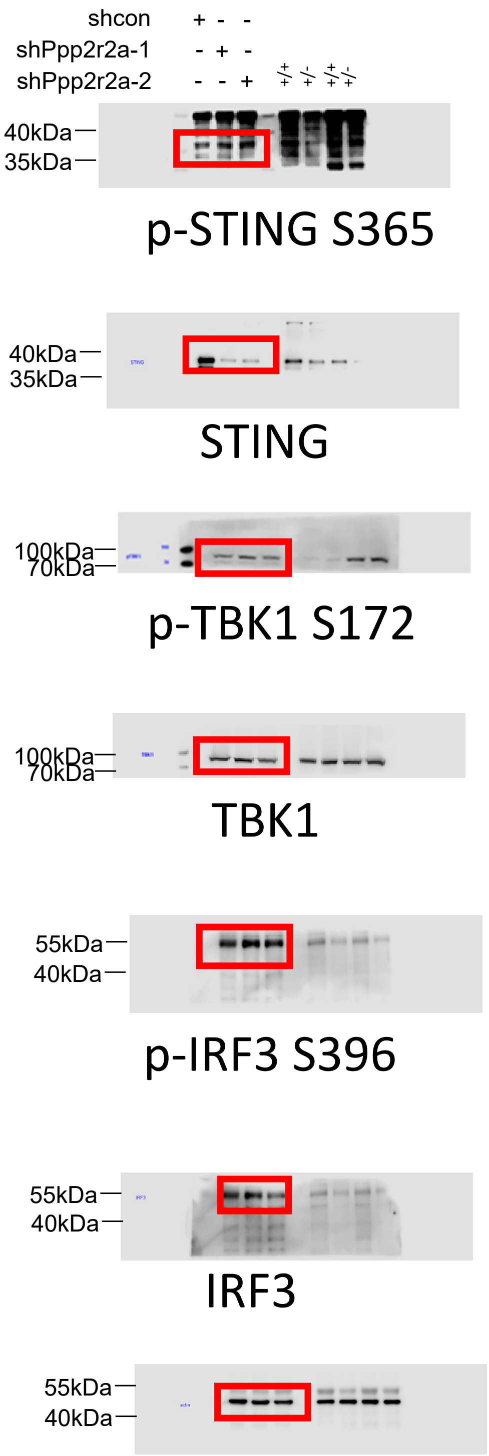

β-Actin (loading control of pSTING, STING, pTBK1, TBK1, pIRF3 and IRF3)

Full unedited blot for Sup Fig8A LLC

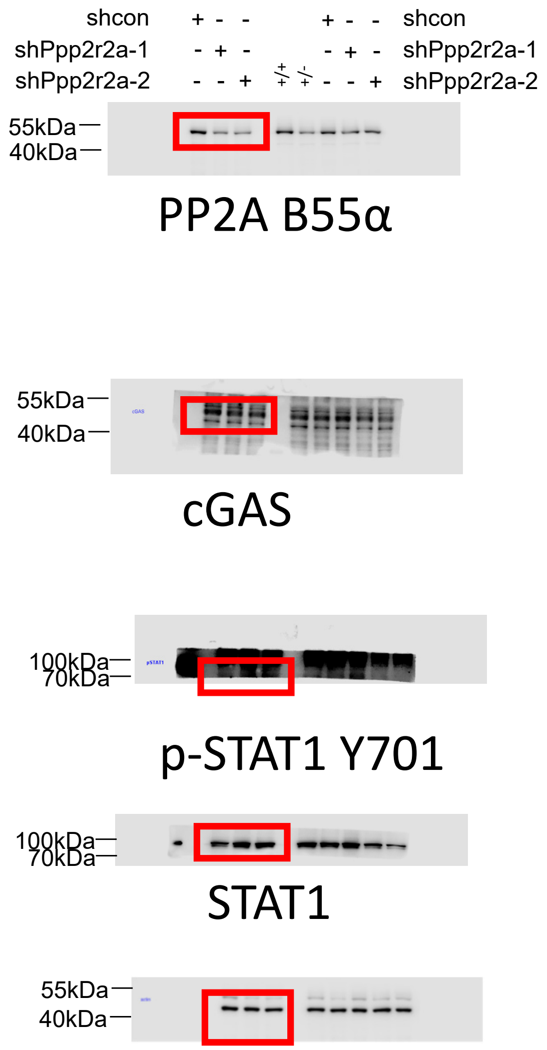

β-Actin (loading control of PP2A B55α , cGAS, pSTAT1 and STAT1)

Full unedited blot for Sup Fig8A LLC

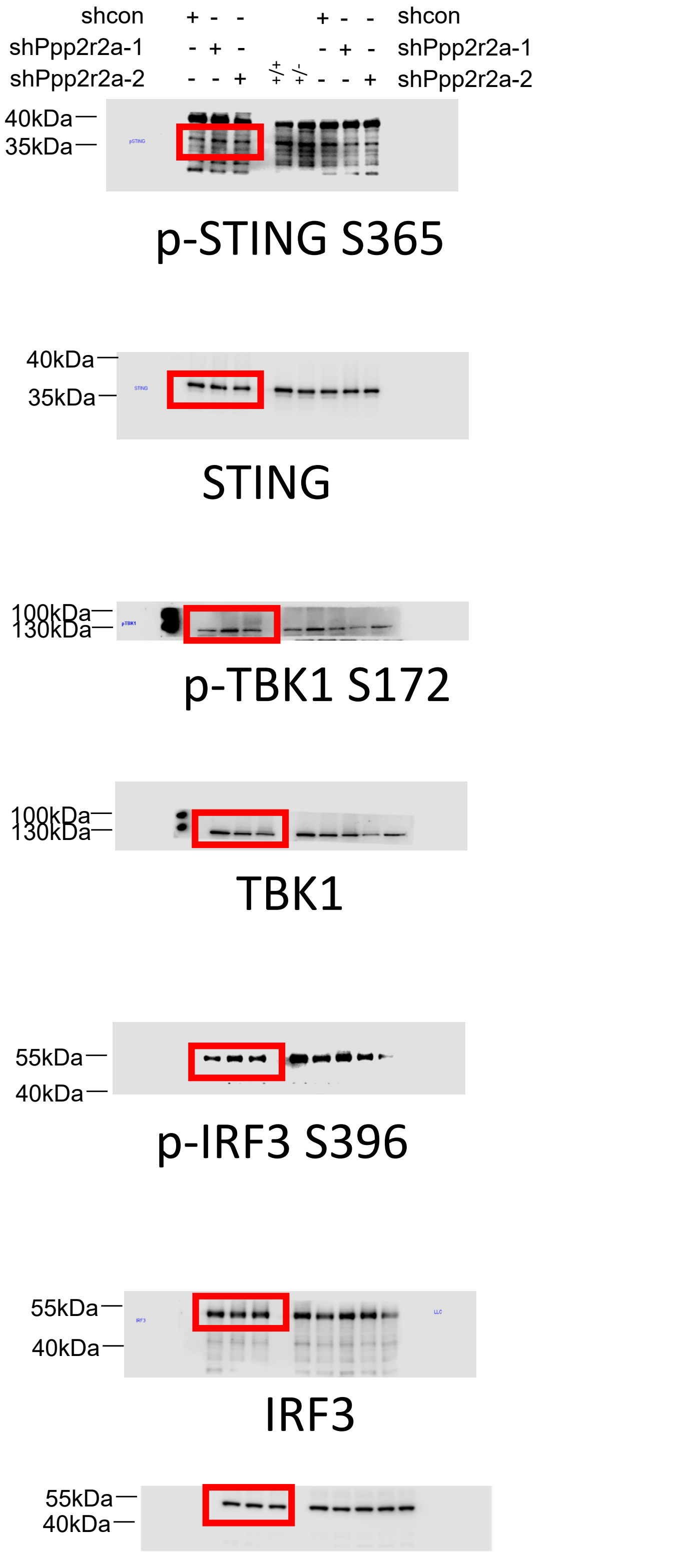

β-Actin (loading control of pSTING, STING, pTBK1, TBK1, pIRF3 and IRF3)

Full unedited blot for Sup Fig8D A549

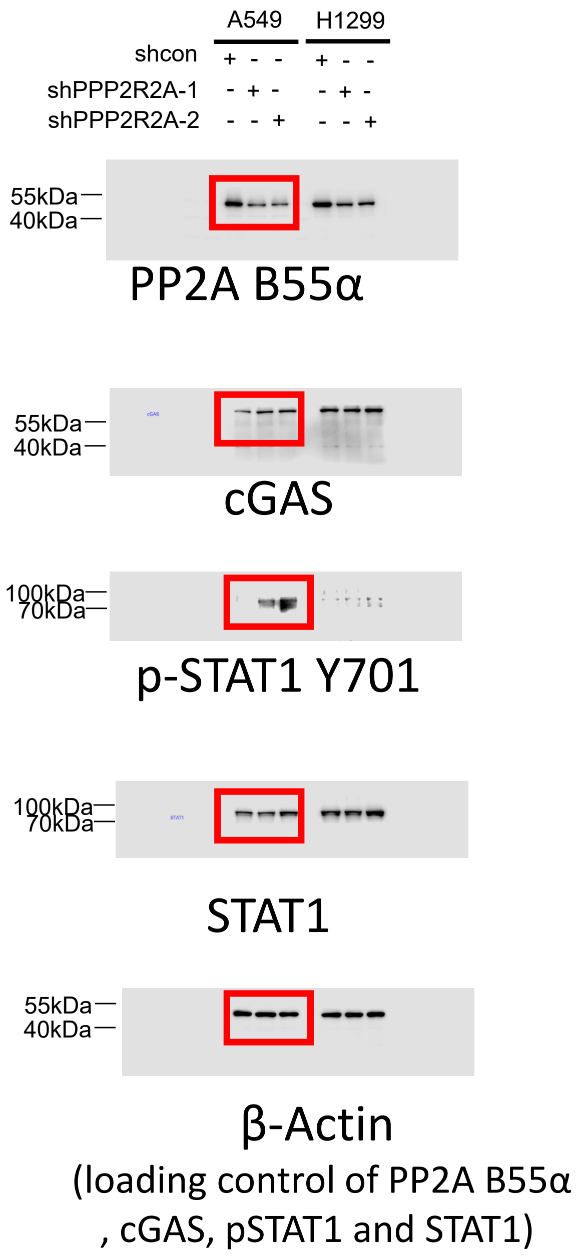

Full unedited blot for Sup Fig8D A549

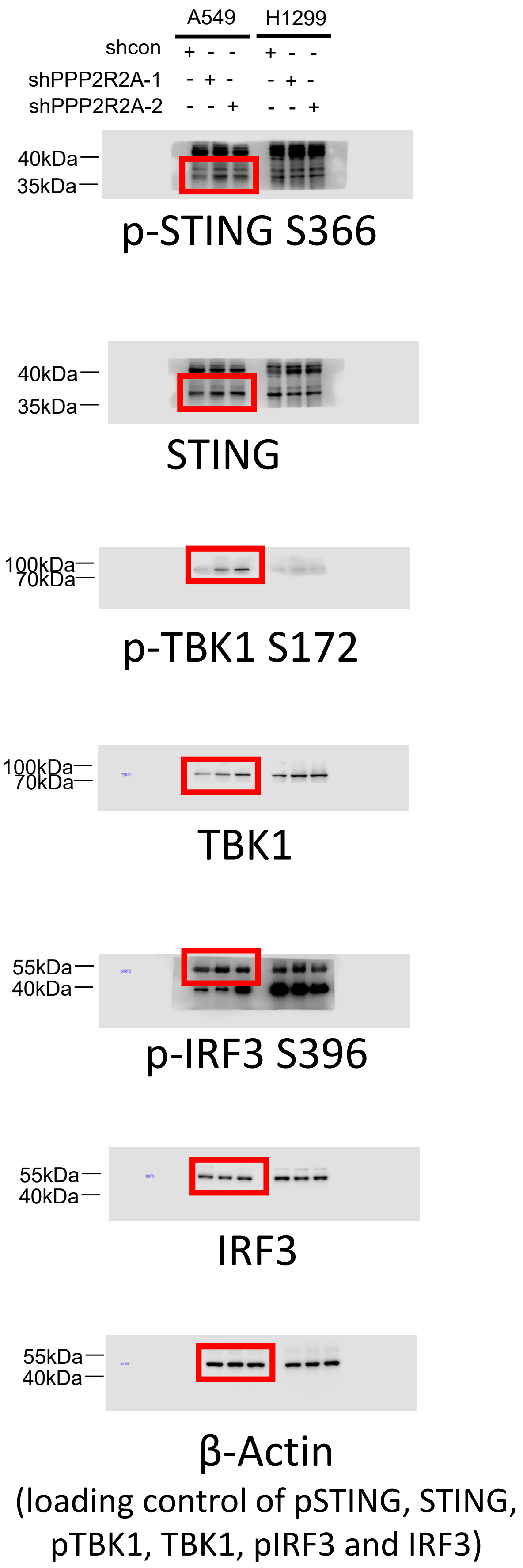

Full unedited blot for Sup Fig8D H1299

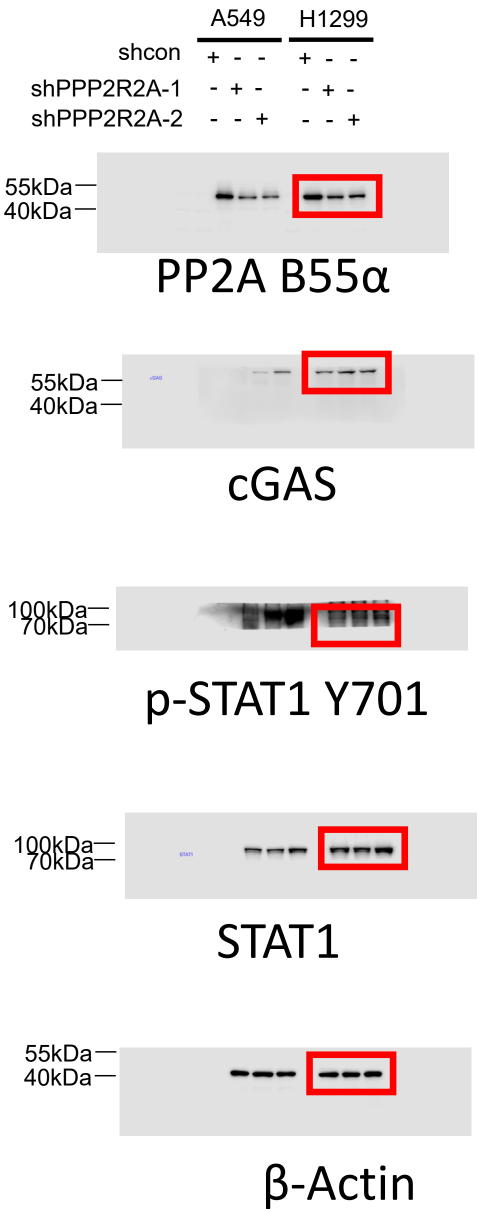

(loading control of PP2A B55α , cGAS, pSTAT1 and STAT1)

Full unedited blot for Sup Fig8D H1299

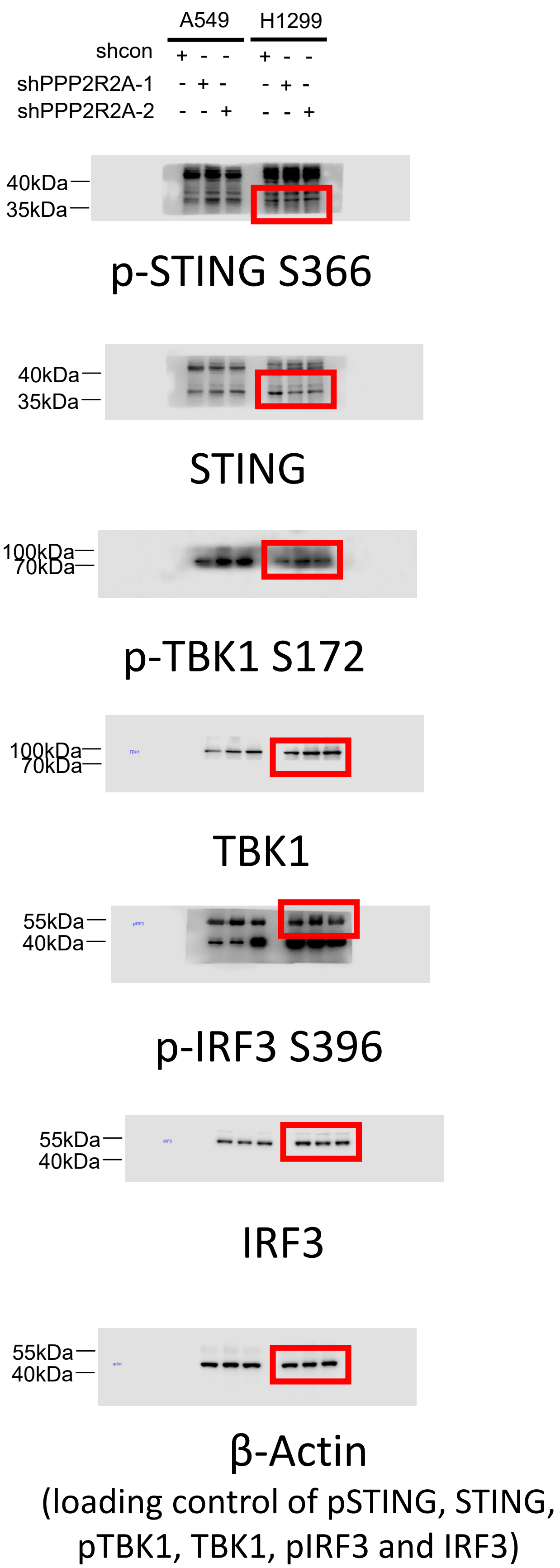

Full unedited blot for Sup Fig10A CMT167

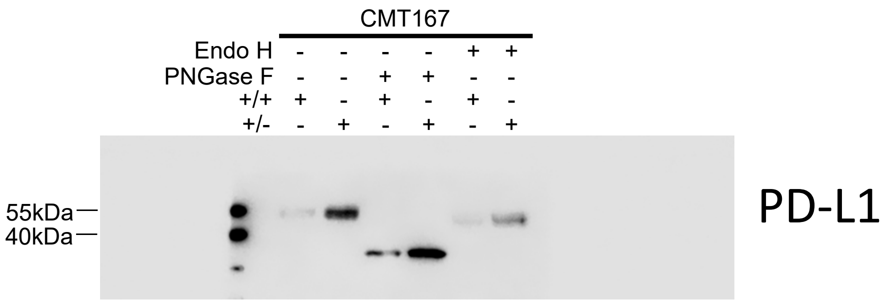

Full unedited blot for Sup Fig10B LLC

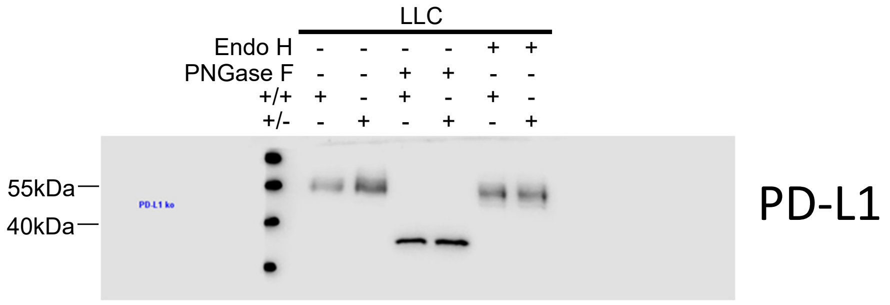

Full unedited blot for Sup Fig10C A549

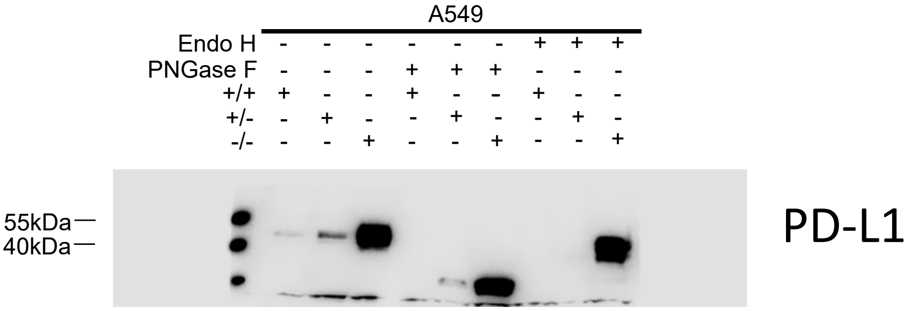

Full unedited blot for Sup Fig10D H1299

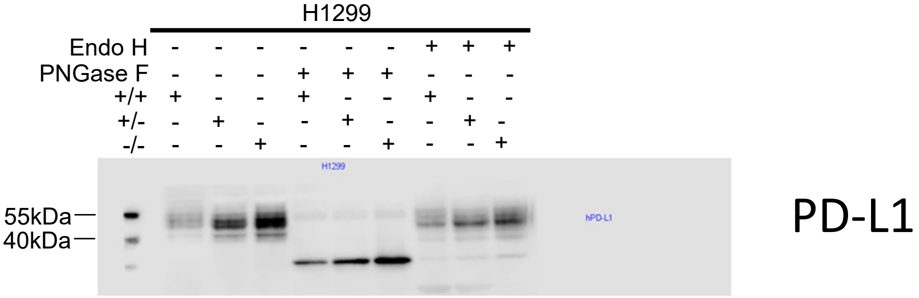

Full unedited blot for Sup Fig11A CMT167

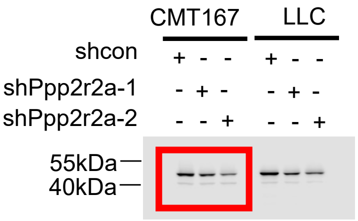

PP2A B55α

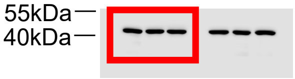

β-Actin (loading control of PP2A B55α)

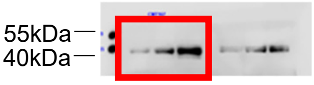

PD-L1

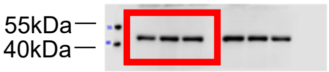

p-GSK-3β Ser9

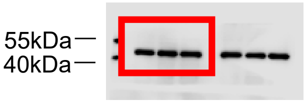

GSK-3β

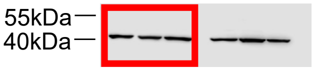

β-Actin (loading control of PD-L1, p-GSK-3β Ser9 and GSK-3β)

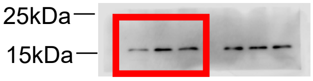

γ-H2AX

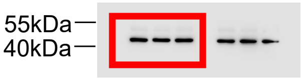

β-Actin (loading control of γ-H2AX)

Full unedited blot for Sup Fig11A LLC

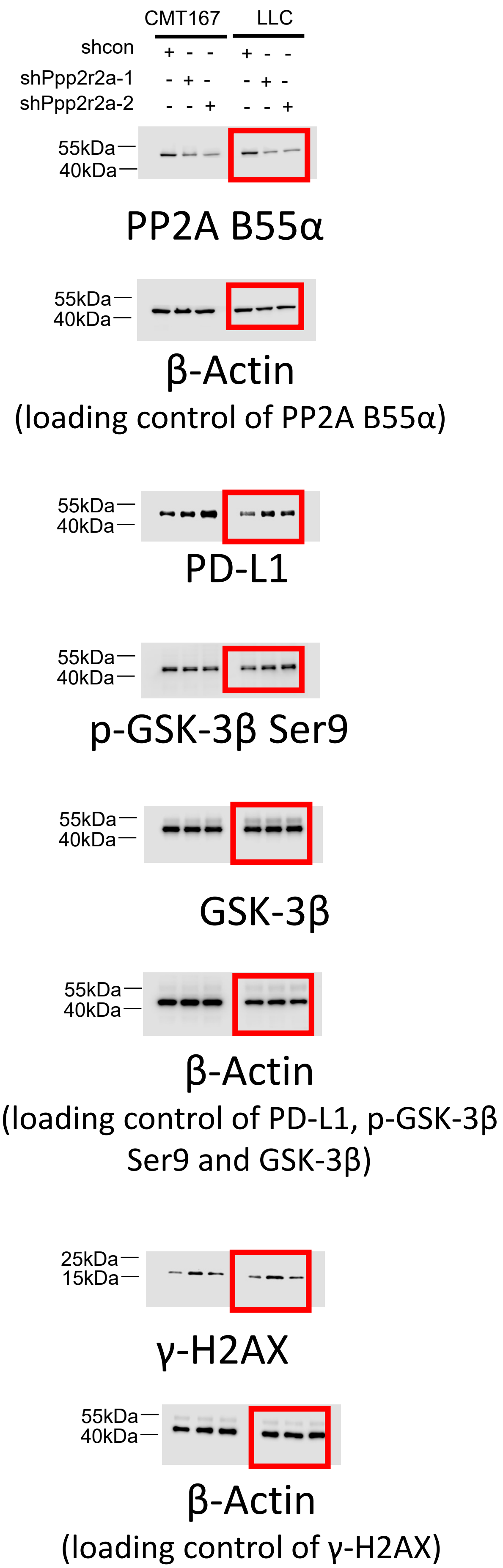

Full unedited blot for Sup Fig11D A549

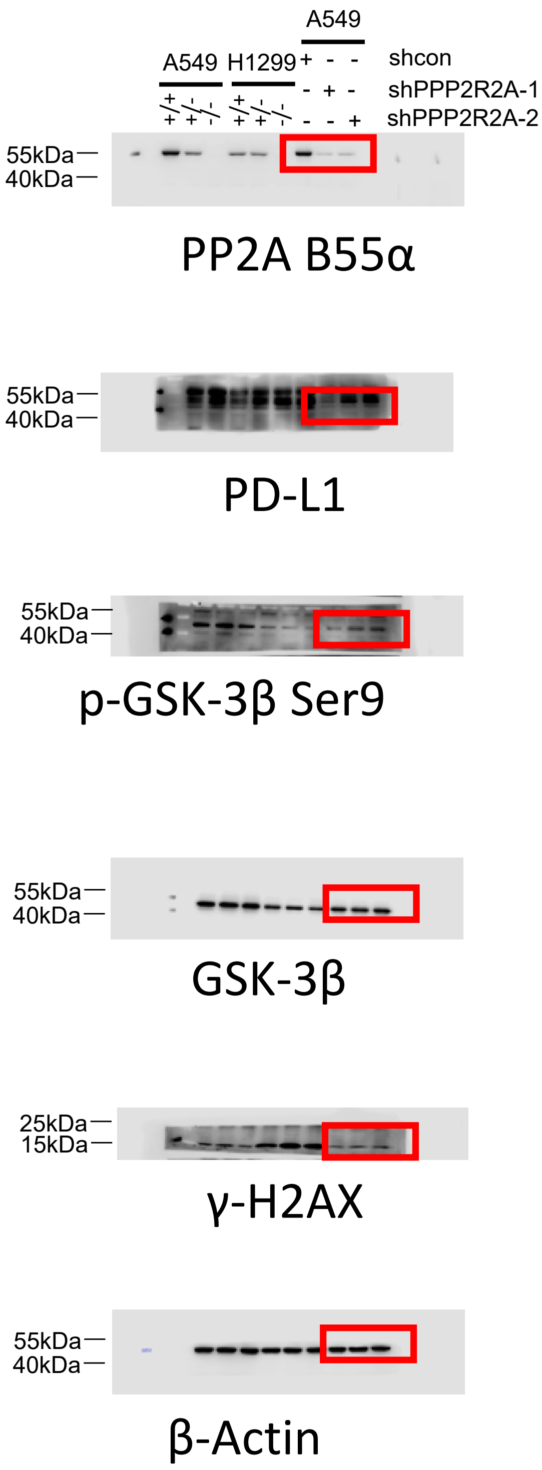

Full unedited blot for Sup Fig11D H1299

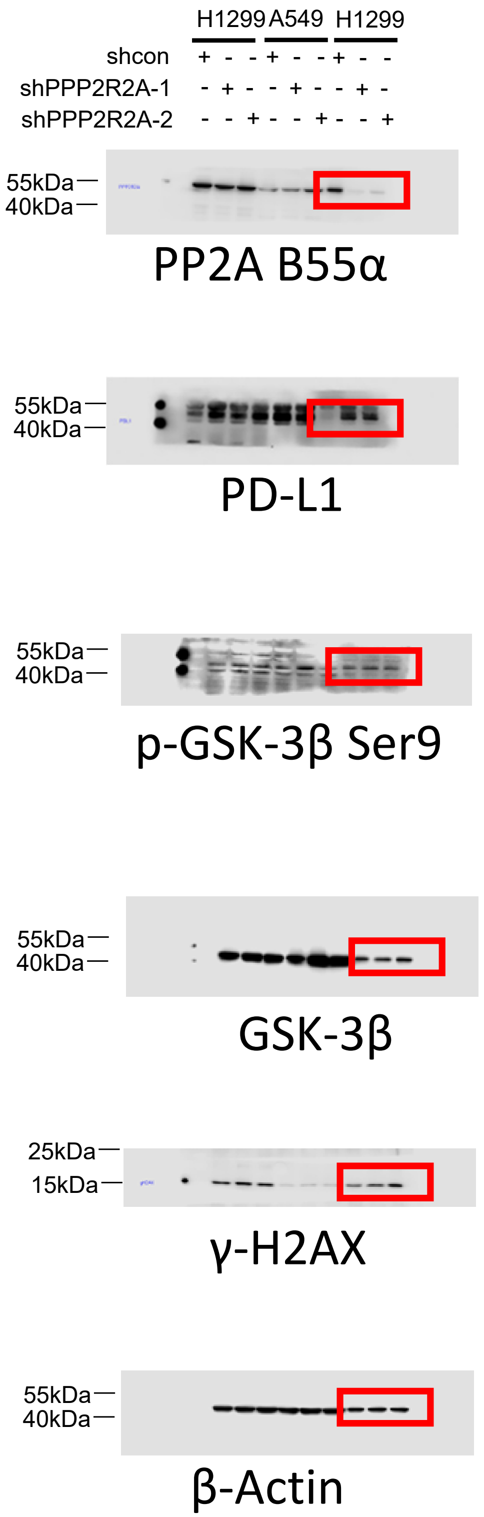

Full unedited blot for Sup Fig11G H1437

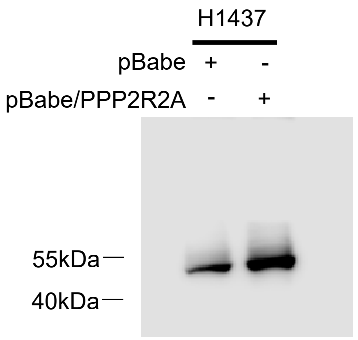

PP2A B55α

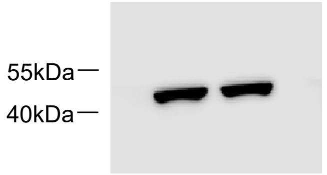

β-Actin
(loading control of PP2A B55α)

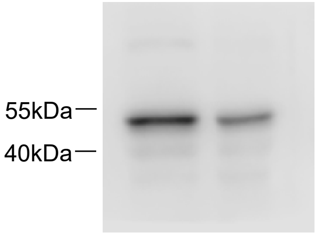

p-GSK-3β Ser9

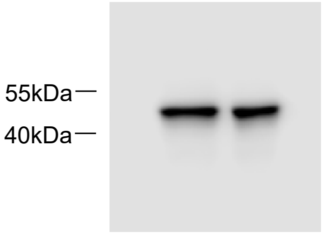

GSK-3β

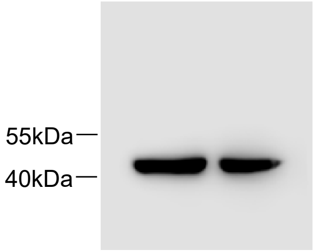

β-Actin
(loading control of p-GSK-3β Ser9 and GSK-3β)

Full unedited blot for Sup Fig12A A549

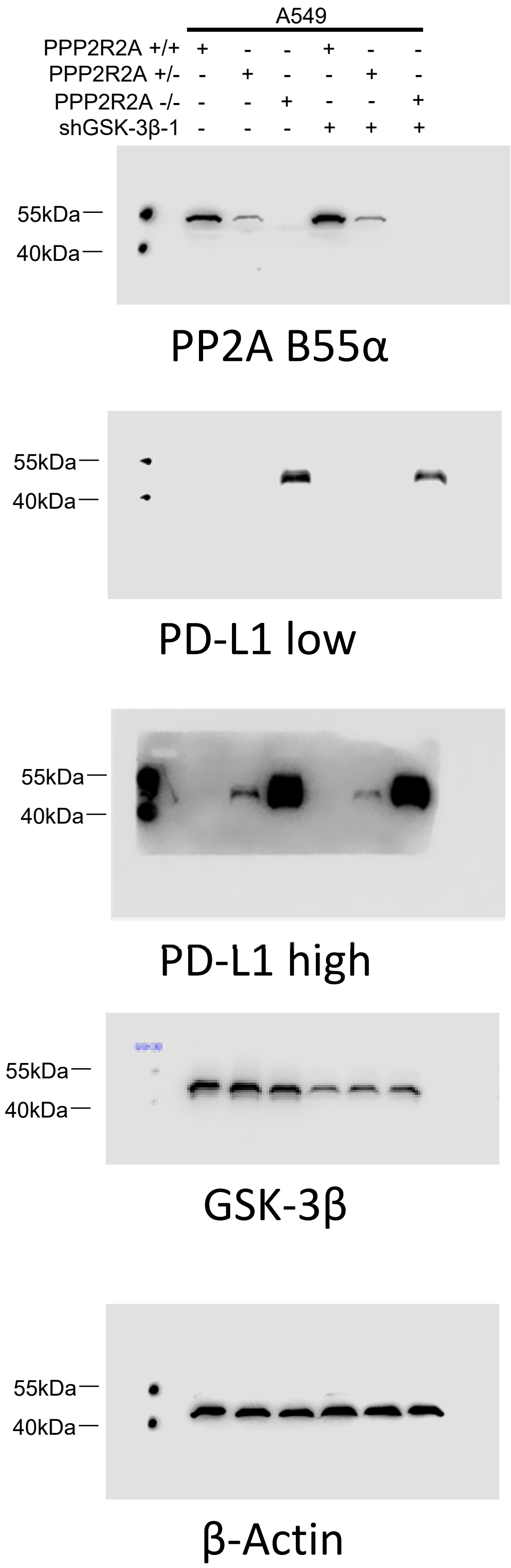

Full unedited blot for Sup Fig12B A549

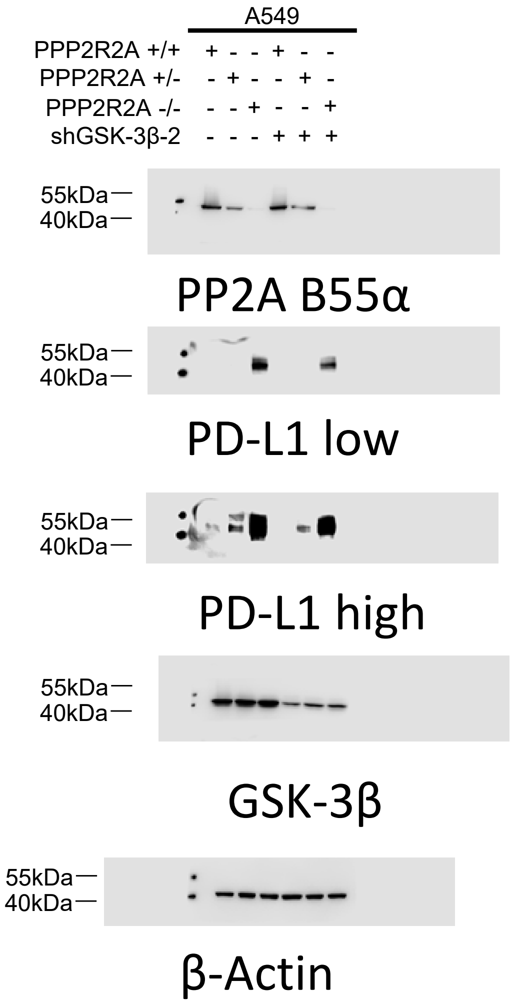

Full unedited blot for Sup Fig12C CMT167

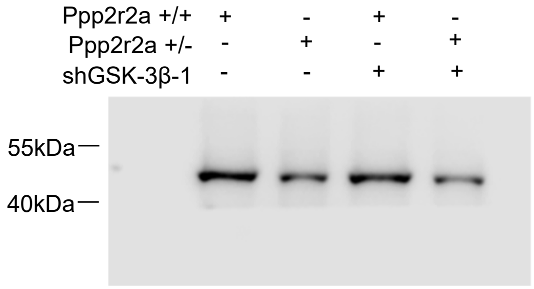

PP2A B55α

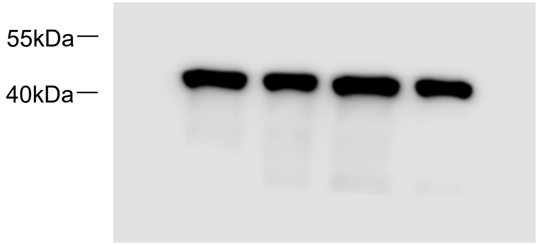

β-Actin (loading control of PP2A B55α)

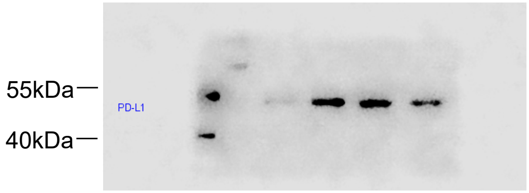

PD-L1

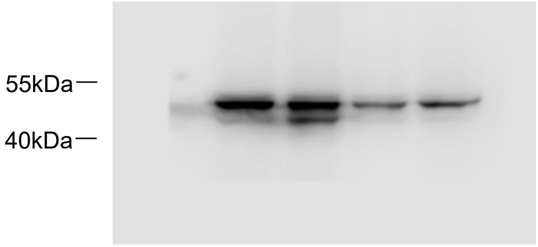

GSK-3β

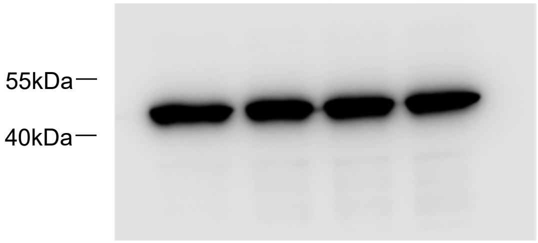

β-Actin (loading control of PD-L1 and GSK-3β)

Full unedited blot for Sup Fig12D CMT167

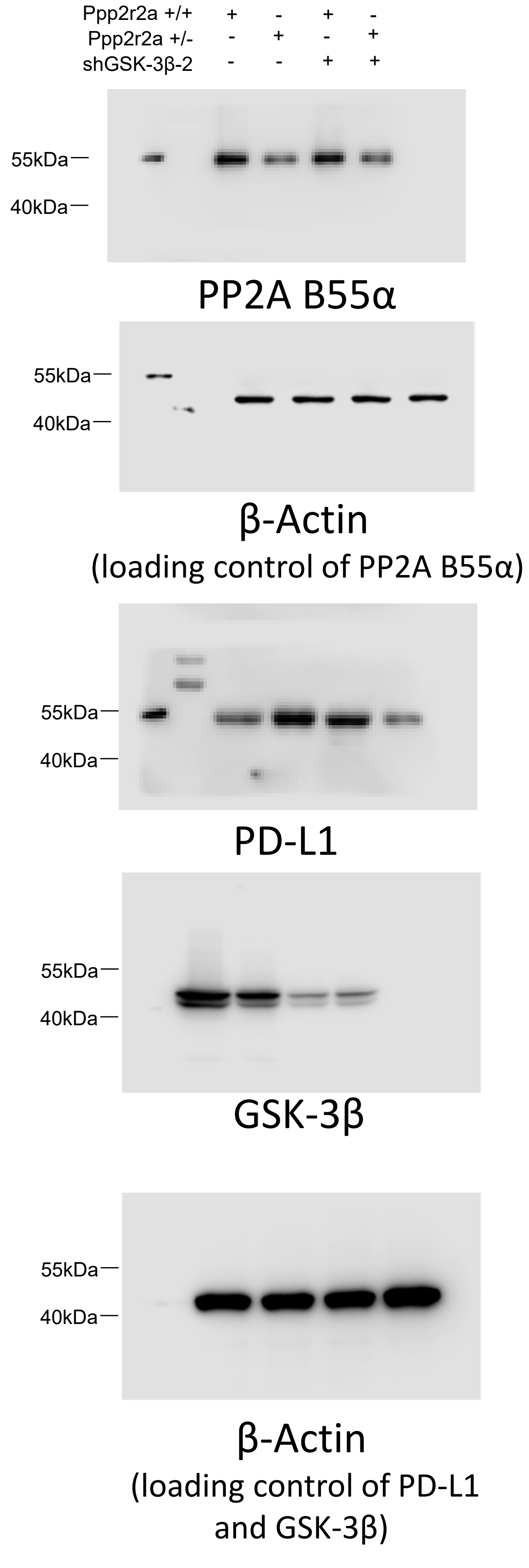

Sup Fig13A CMT167

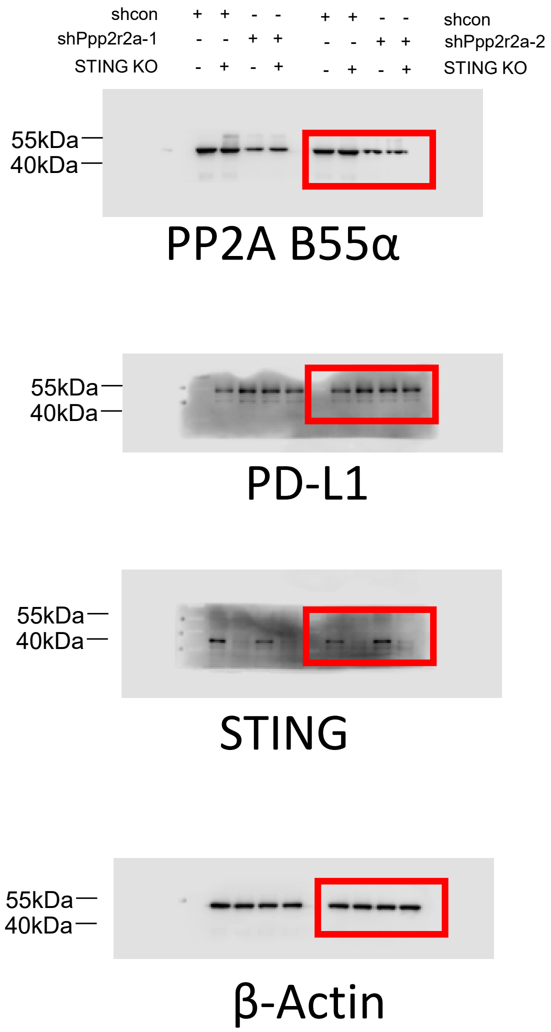

Sup Fig13C A549

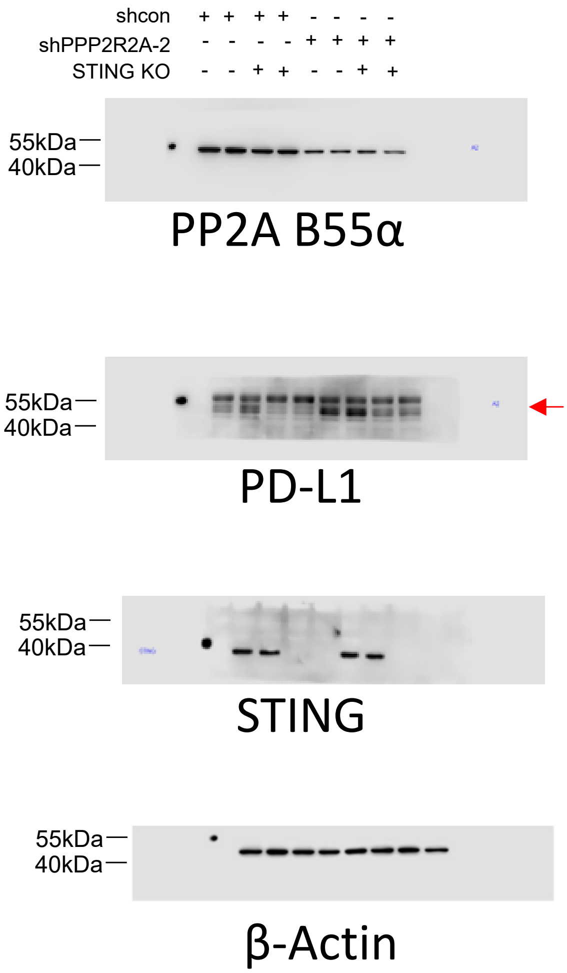

Supplement: Unedited blot and gel images [file jci-136-193354-s041.pdf]
